# The Fragment-Based Development of a Benzofuran Hit as a New Class of *Escherichia coli* DsbA Inhibitors

**DOI:** 10.3390/molecules24203756

**Published:** 2019-10-18

**Authors:** Luke F. Duncan, Geqing Wang, Olga V. Ilyichova, Martin J. Scanlon, Begoña Heras, Belinda M. Abbott

**Affiliations:** 1 Department of Chemistry and Physics, La Trobe Institute for Molecular Science, La Trobe University, Melbourne 3086, Australia; 2 Department of Biochemistry and Genetics, La Trobe Institute for Molecular Science, La Trobe University, Melbourne 3086, Australia; 3 Medicinal Chemistry, Monash Institute of Pharmaceutical Sciences, Monash University, 381 Royal Parade, Parkville 3052, Australia; 4 ARC Training Centre for Fragment Based Design, Monash Institute of Pharmaceutical Sciences, Monash University, 381 Royal Parade, Parkville 3052, Australia

**Keywords:** fragment-based drug discovery, *Escherichia coli*, antibiotic resistance, DsbA inhibition, benzofuran, disulfide bond, bacterial virulence

## Abstract

A fragment-based drug discovery approach was taken to target the thiol-disulfide oxidoreductase enzyme DsbA from *Escherichia coli* (*Ec*DsbA). This enzyme is critical for the correct folding of virulence factors in many pathogenic Gram-negative bacteria, and small molecule inhibitors can potentially be developed as anti-virulence compounds. Biophysical screening of a library of fragments identified several classes of fragments with affinity to *Ec*DsbA. One hit with high mM affinity, 2-(6-bromobenzofuran-3-yl)acetic acid (**6**), was chemically elaborated at several positions around the scaffold. X-ray crystal structures of the elaborated analogues showed binding in the hydrophobic binding groove adjacent to the catalytic disulfide bond of *Ec*DsbA. Binding affinity was calculated based on NMR studies and compounds **25** and **28** were identified as the highest affinity binders with dissociation constants (*K*_D_) of 326 ± 25 and 341 ± 57 µM respectively. This work suggests the potential to develop benzofuran fragments into a novel class of *Ec*DsbA inhibitors.

## 1. Introduction

The rise of multidrug-resistant bacteria has rendered many of our current antibiotics ineffective. With few antibacterial drugs in the development pipeline, antibiotic resistance has been identified as a significant global health threat, highlighting the urgent need for new classes of antibacterial drugs with novel mechanisms of action. Bacteria rely on the biosynthesis of virulence factors to establish an infection in a host and cause disease. Many of these virulence factors, such as secreted toxins and surface proteins, require folding into their native state by the thiol-disulfide oxidoreductase enzyme DsbA. It has been shown that DsbA null mutant bacteria show pleiotropic phenotypes with defects in secretion and motility [1]. The loss of functional DsbA in Gram-negative bacteria generally leads to attenuated virulence, increased sensitivity to antibiotics, and reduced fitness in animal models of infection [2,3,4,5,6]. Chemical inhibition of DsbA thus represents an attractive approach to attenuate bacterial virulence and could potentially lead to the development of novel antibacterial therapeutics [7].

**Figure 1:**
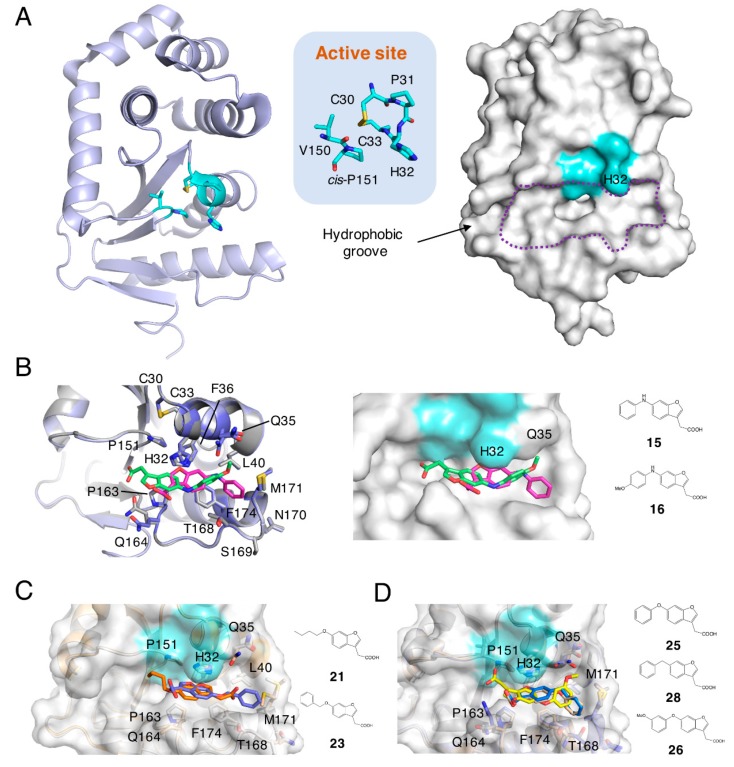
Structural characterisation of benzofuran analogues binding to DsbA. (**A**) Structure of apo *Escherichia coli* DsbA. Left panel: Crystal structure of *E. coli* DsbA (PDB ID: 1FVK) is presented as a cartoon. The active site is shown in cyan. The disulfide bond is shown in yellow sticks. Inlet: A close-up view of the active site Cis30-Pro31-His32-Cis33 motif and *cis*-Pro loop (Val150-*cis*-Pro151). Right panel: Surface representation of *E.coli* DsbA in the same orientation. The hydrophobic groove of DsbA is highlighted by a dashed line. The active site is shown in cyan. (**B**) Superposition of the co-crystal structures of the **15** (pink)*–E. coli* DsbA (*Ec*DsbA) (grey) complex and the **16** (green)-*Ec*DsbA (light blue) complex. Left panel: The residues within 4 Å of the ligands are shown as sticks and labelled individually. Right panel: Surface representation of the *Ec*DsbA complexes. The active site is shown in cyan. (**C**) Superposition of the co-crystal structures of **21** (orange)-*Ec*DsbA (light orange) and **23** (purple)-*Ec*DsbA (grey) complexes. The surface of **23**-complexed *Ec*DsbA is shown in white. The active site is shown in cyan. (**D**) Superposition of the co-crystal structures of **25** (salmon)-*Ec*DsbA (salmon), **28** (blue)-*Ec*DsbA (purple), and **26** (yellow)-*Ec*DsbA (grey) complexes. The surface of **26**-complexed *Ec*DsbA is shown in white. The active site is shown in cyan.

The highly oxidising protein DsbA catalyses disulfide bond formation between two cysteine residues of unfolded substrates in the periplasm of Gram-negative bacteria [3,8]. The structure of DsbA is comprised of a thioredoxin domain with an inserted helical domain [9,10,11]. Like many other thiol-disulfide oxidoreductase enzymes, the active site of DsbA is comprised of the characteristic CXXC motif (Cys30–Pro31–His32–Cys33 in *Escherichia coli* DsbA) and a highly conserved *cis*-proline in an adjacent loop which has been shown to play a key role in the binding of substrates (Figure 1A) [12]. Disulfide exchange between oxidised DsbA and its unfolded substrates leaves DsbA in its reduced inactive form which is in turn oxidised by its redox partner DsbB to reprime the catalytic cycle. Adjacent to the active site is a hydrophobic groove which serves as a site for both DsbB and unfolded substrates to form a complex (Figure 1A).

Previous work on the development of DsbA inhibitors has included small peptide mimetics of a natural substrate and more recently small molecule inhibitors. Martin and co-workers reported a heptapeptide inhibitor of DsbA of *Escherichia coli* (*Ec*DsbA) that was an analogue of the *Ec*DsbB periplasmic loop [13]. The heptapeptide bound to *Ec*DsbA (KD = 2.0 ± 0.3 µM) and inhibited its activity (IC_50_ = 5.1 ± 1.1 μM). The crystal structure showed that the peptide occupies the hydrophobic groove and forms a covalent disulfide bond with Cys30 of DsbA. Although peptides are difficult to be developed into therapeutics due to their limitations, i.e., poor stability, membrane permeability, and oral availability, this study demonstrates that the hydrophobic groove is a promising target site to block DsbA function.

The development of small molecule inhibitors against the DsbA-DsbB system has been actively pursued [14,15,16]. In addition to development of DsbB inhibitors, a fragment-based approach was taken by Adams and co-workers in which a library of 1132 fragments, sourced from the Maybridge Ro3 collection, was screened against oxidised *Ec*DsbA [17]. This approach identified several structural classes of fragments with weak affinity to *Ec*DsbA. A phenylthiazole fragment was synthetically elaborated into a higher affinity compound with mid-µM inhibition, providing the first example of small molecule inhibitors of *Ec*DsbA. Crystal structures of *Ec*DsbA in complex with the inhibitors indicated binding to the hydrophobic groove adjacent to the active site [17]. Herein, the chemical elaboration of a benzofuran fragment identified by the screening technique already described is explored as an inhibitor of *Ec*DsbA.

## 2. Results and Discussion

### 2.1. Chemical Synthesis and X-Ray Crystallography

**Scheme 1:**
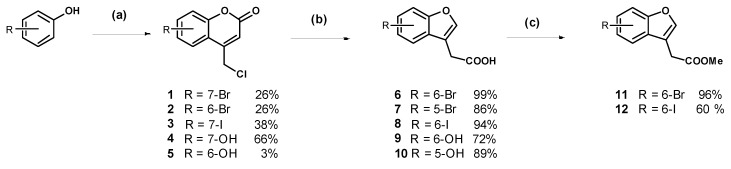
Synthesis of key benzofurans **6**–**12** for elaboration. Reagents and conditions: (a) Ethyl 4-chloroacetoacetate, H_2_SO_4_ or MsOH, 0 °C, 2 h, RT, and 24 h; (b) 2 M NaOH, 80 °C, and 16 h; (c) CH_3_OH, SOCl_2_, reflux, and 16 h.

We previously reported the fragment screening campaign against *Ec*DsbA and identified 37 fragment hits which are clustered into eight different chemical classes [17]. In this work, we focus on elaboration of benzofuran fragment hits identified in the screen using a structure-guided approach. Assessing the identified hit fragment (**6**), the 6-bromo substituent was identified as an obvious site for elaboration. This site was amenable to coupling reactions to extend the fragment from the benzene ring of the benzofuran moiety and introduce a range of functionalities to probe for favourable interactions with residues in the *Ec*DsbA binding site. Hydroxy analogues of the hit fragment were also synthesised to broaden the chemistry that could be applied for elaboration. The benzofuran precursors were prepared in two steps through Pechmann condensation between a substituted phenol and ethyl 4-chloroacetoacetate followed by Perkin rearrangement into the corresponding benzofuran by heating in sodium hydroxide (Scheme 1).

The withdrawing effect of the bromine substituents resulted in poor yields of 26% for coumarins **1** and **2**, while a less electron withdrawing halogen substituent resulted in a modestly improved yield for the 7-iodo analogue **3**. A higher yield was achieved for 7-hydroxycoumarin **4**; however, the same was not observed for 6-hydroxycoumarin **5**. Coumarins **1**–**5** were rearranged to their corresponding benzofurans in high to excellent yields giving bromo and hydroxy functionality at both the 5-position and bromo, iodo, and hydroxy functionality at the 6-position. Benzofurans **6**–**10** were then used as precursors for fragment elaboration. The carboxylic acid group was protected as the methyl ester for the reactions where acid sensitivity was of concern.

**Scheme 2:**
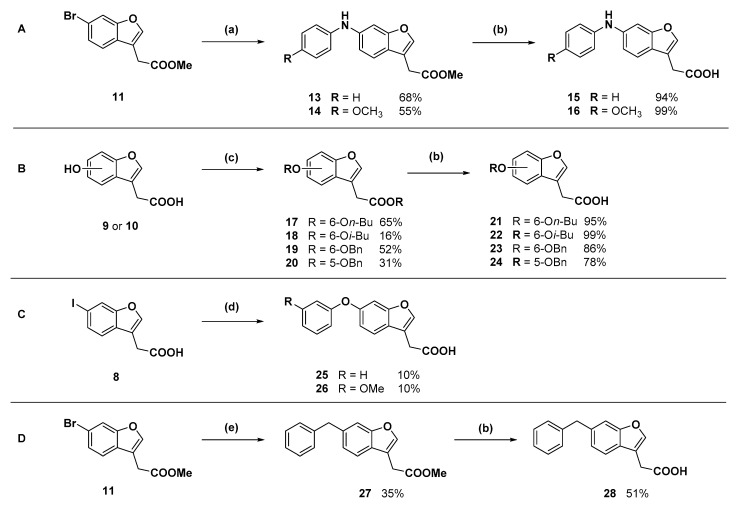
(**A**) Aryl amine formation at C-6 via Buchwald–Hartwig coupling and subsequent ester hydrolysis. (**B**) Alkyl ether formation at the 6-position via Williamson ether synthesis. (**C**) Aryl ether formation at C-6 via Ullmann-type coupling. (**D**) Benzyl formation at C-6 via Negishi coupling and subsequent hydrolysis. Reagents and conditions: (a) Aryl amine, K_2_CO_3_, Pd_2_(dba)_3_, XPhos, *t*-BuOH, reflux, 16 h; (b) 2 M NaOH, 80 °C, 16 h; (c) alkyl bromide, K_2_CO_3_, CH_3_CN, reflux, 16 h; (d) substituted phenol, CuI, picolinic acid, K_3_PO_4_, CH_3_SOCH_3_, 90 °C, 24 h. (**e**) (1) Zn, LiCl, BnBr, THF, 0 °C, 1.5 h; (2) **11**, Pd(OAc)_2_, XPhos, THF, 50 °C, 18 h.

The first series of analogues were synthesised via Buchwald–Hartwig coupling to introduce amine functionality at C-6 through the palladium catalysed coupling of amines with the 6-bromo starting material **11** (Scheme 2A). The methyl ester was chosen over the carboxylic acid **6** to avoid any interactions with the basic amine coupling partners and to avoid the complication of isolating a zwitterionic coupled product from a potentially complicated crude mixture. The aniline **13** and *p*-anisidine **14** analogues were isolated in fair yields (68% and 55% respectively) and subsequent hydrolysis of the methyl ester gave **15** and **16** as the carboxylic acids in excellent yields. The alkyl amines of *n*-butylamine and *i*-propylamine failed to couple under the same conditions.

The synthesised analogues were soaked into crystals of *Ec*DsbA and X-ray diffraction data was collected for each analogue in order to determine their site of binding. Crystal structures of aniline **15** and anisidine **16** revealed that both ligands were bound in the hydrophobic end of the binding groove adjacent to the catalytic disulfide bond (Figure 1B, the 2Fo-Fc electron density map and omit map for compounds are shown in Supplementary Figure S1). Elucidation of the crystal structure of *Ec*DsbA in complex with aniline **15** (Figure 1B) showed a π-stacking interaction between the benzofuran core and His32 of the active site CPHC motif. The carboxy moiety of **15** was oriented towards the left-hand side of the binding groove, where more polar residues are located. The phenyl ring of the aniline moiety occupied the hydrophobic end of the binding pocket with the nitrogen atom of the amine linker positioned in close proximity to Gln35. The side chain of Gln35 is highly flexible and adopts two conformations in the co-structure, suggesting that **15** does not stabilize the Gln35 side chain through the hydrogen bond. In general, the interaction of **15** with the hydrophobic groove residues His32, Phe36, Leu40, Pro163, Gln164, Thr168, Phe174, and Met171 strongly resembles the previously elaborated phenylthiazole series [17], suggesting a similar mode of binding for these two chemical series. The co-structure of **16** showed the *p*-methoxy group of the anisidine moiety resulted in a shift of the benzofuran core further down into the more hydrophilic region of the binding groove. Although anisidine **16** was occupying a larger region of the *Ec*DsbA binding groove, the shift appeared to have interrupted the π-stacking interactions the benzofuran was making with the His32 side chain previously (Figure 1B and Figure S1).

Alkyl ethers were next formed at the 6-position coupling alkyl bromides to the 6-hydroxybenzofuran **9** via Williamson ether synthesis (Scheme 2B). These conditions also esterified the carboxylic acid moiety which required subsequent base hydrolysis. This coupling resulted in the *n*-butyl **21**, *i*-butyl **22**, and benzyl **23** ethers which were soaked into crystals of *Ec*DsbA for X-ray diffraction data collection. Analysis of the electron density maps revealed that the **21** and **23** bound in the hydrophobic binding groove of *Ec*DsbA (Figure 1C, the 2Fo-Fc electron density map, and the omit maps for these compounds are shown in Supplementary Figure S1). Common among each of the ethers was a π-stacking interaction between the benzofuran moiety and His32. Interestingly, the binding poses of alkyl ether **21** contrasted with amines **15** and **16**, in that the alkyl ether moiety was facing into the more hydrophilic region of the binding groove with the carboxylic acid located in the hydrophobic end of the groove. The benzyl ether **23** displayed a similar orientation to that observed in previous aromatic derivatives with the benzyl ether orienting towards the hydrophobic end of the binding groove. The conformation of alkyl ether **21** was not considered ideal as the alkyl chain of the ether did not appear to be making any favourable interactions with the residues in the binding site. For this reason, aromatic groups at the 6-position were preferred to maintain the binding pose.

To test the effect of the site of elaboration, the 5-benzyl ether analogue was synthesised using 5-hydroxybenzofuran **10** (Scheme 2B). The reaction was refluxed in acetone in an attempt to achieve milder conditions to avoid any thermal decomposition that may have been occurring previously; however, the lower resulting yield suggested that more forcing conditions were required. Hydrolysis of the benzyl ester gave benzofuran **24**, which, after soaking into *Ec*DsbA crystals, did not show clear enough electron density to unambiguously determine the binding pose.

**Figure 2:**
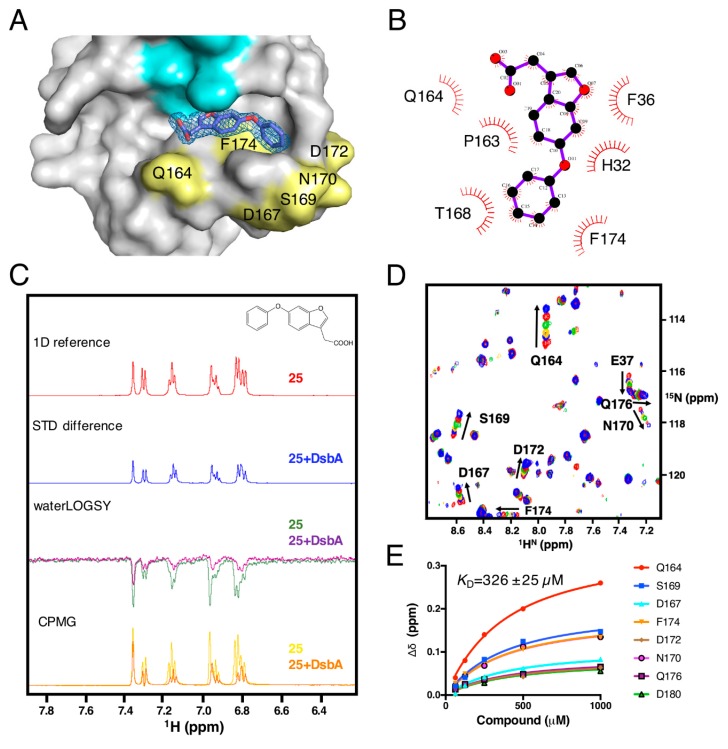
Biophysical characterisation of compound **25**. (**A**) Crystal structure of *Ec*DsbA in complex with **25**. Simulated annealing omit σ_A_-weighted mFo-DFc electron density map for **25** is contoured at 2.5σ and shown as blue mesh. The active site is shown in cyan. The residues whose chemical shifts are strongly perturbed by **25** at 1 mM (chemical shift perturbations > 0.04 ppm) are coloured in yellow and labelled. (**B**) LIGPLOT analysis of the interaction between DsbA and **25**. (**C**) Binding of **25** to *Ec*DsbA was assessed by ligand-detected NMR spectroscopy. Comparison of the 1D ^1^H NMR spectrum of **25** (green) and the saturation transfer difference (STD) spectrum of **25** + DsbA (blue) shows magnetization transfer from DsbA to the aromatic resonances of **25**. waterLOGSY NMR spectrum shows that the aromatic resonances of **25** become less negative in the presence of DsbA (purple) compared to **25** alone (green). The CPMG NMR spectrum (τ = 350 ms) of **25** shows attenuation of its aromatic resonances in the presence of DsbA (orange) compared to **25** alone (yellow). (**D**) A portion of the ^1^H-^15^N HSQC spectra of ^15^N-labelled *Ec*DsbA showing chemical shift perturbation (CSP) upon titration of increasing concentrations of **25** (0, 0.0625, 0.125, 0.25, 0.5, and 1 mM). The direction of the CSP is indicated by black arrows. (**E**) The equilibrium dissociation constant (*K*_D_) of **25** is determined by measurement of chemical shift perturbation (∆δ) in the ^1^H-^15^N-HSQC spectra of *Ec*DsbA as a function of compound concentration.

Synthesis of diaryl ether **25** was initially attempted using 6-bromobenzofuran **6** under Ullmann coupling conditions but only moderate conversion to the desired product was observed by HPLC after 48 h at 90 °C. Far superior conversion was observed using the more reactive iodobenzofuran **8**, resulting in the cross-coupled diaryl ethers **25** and **26** (Scheme 2C). Unfortunately, both products were very difficult to isolate from the crude reaction mixture, requiring normal phase chromatography and recrystallisation followed by reverse-phase high performance liquid chromatography. Both diaryl ethers **23** and **25**, were soaked into crystals of *Ec*DsbA and co-crystals were diffracted to 2.0 and 1.77 Å of resolution, respectively. Phenyl ether **25** bound to the *Ec*DsbA groove neighbouring the active site (Figure 1D and Figure 2A) showing a binding pose to the benzyl ether **23** (Figure 1C) and the direct anilino analogue **15** (Figure 1B).

The co-structure was more comparable with the direct anilino analogue **15**. The phenoxy group of **25** occupied the hydrophobic end of the binding groove with the oxygen atom of the ether linker at a distance of 4.1 Å from Gln35. The side chain of Gln35 is conformationally flexible in the crystal structure and therefore unlikely to form a hydrogen bond with **25** (Figure 1D). The carboxyl group of **25** is positioned more closely to the imidazole side chain of His32 (N–O distance = 3.7Å) compared to that of **15** (N–O distance = 4.5Å) and possibly formed a weak polar interaction. 

As the crystal structure of the *p*-anisidine analogue **16** showed that the installation of the *p*-methoxy group induced a shift of the benzofuran core away from His32 (relative to anilino **15**), the *m*-methoxy substituted phenyl ether **26** was synthesised to observe whether the shift could be avoided. The co-structure (Figure 1D) showed a horizontal rotation relative to the unsubstituted phenoxy **25** (comparable to **16**) but notably a less pronounced shift into the left-hand side of the binding groove, meaning the π-stacking interaction between the benzofuran core and His32 could be better retained. The *m*-methoxy group of **26** may potentially form a polar interaction with side chain of Gln35, although electron density for this compound was insufficient to unambiguously define the interaction (Figure S1).

To investigate the importance of a hydrogen bond donor/acceptor as a linker, benzyl analogue **28** was synthesised featuring a methylene linker. Benzylzinc bromide was prepared from benzyl bromide and zinc powder [18], then coupled to the 6-bromobenzofuran **11** via Negishi coupling to afford the product **27** (Scheme 2D). The methyl ester was hydrolysed to give the benzyl analogue **28** as the desired carboxylic acid. The co-structure of **28** bound to *Ec*DsbA was determined using X-ray crystallography which showed that the methylene linker had little to no effect on the binding conformation relative to the anilino **15** and phenoxy **25** analogues (Figure 1D). 

**Scheme 3:**
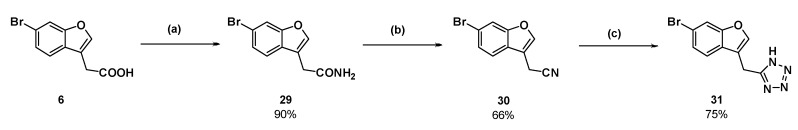
Synthesis of tetrazole 4.27 as a carboxy isostere. Reagents and conditions: (a) (1) PCl_5_, CH_2_Cl_2_, reflux, 1 h; (2) NH_3_, 5 m. (b) SOCl_2_, toluene, reflux, 1.5 h. (c) NaN_3_, Et_3_N·HCl, toluene, reflux, 3 days.

With a series of derivatives prepared by substitution at C-6, attention was turned to the carboxy moiety which until now had been maintained for aqueous solubility. A tetrazole isostere was synthesised in three steps via an amide and nitrile intermediate (Scheme 3). The acid chloride of benzofuran **6** was formed in situ then exposed to ammonia gas that formed the primary amide **29**. Refluxing of **29** in toluene with thionyl chloride dehydrated the amide to the nitrile **30** which then underwent a 2,3-addition with sodium azide to form the tetrazole **31**. Unfortunately, the tetrazole did not produce electron density upon analysis by X-ray crystallography.

**Scheme 4:**
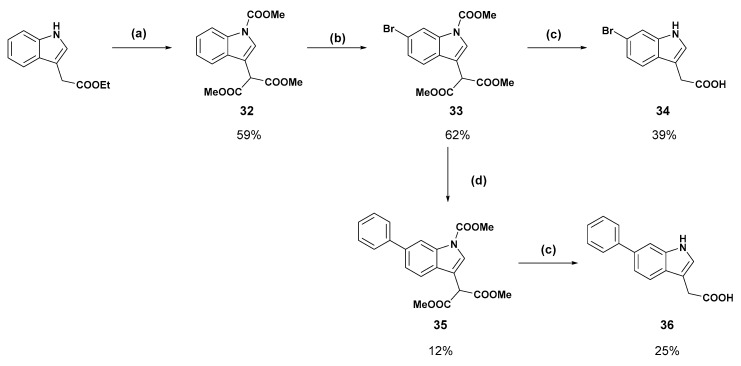
Synthesis of 6-bromo and 6-phenyl indole derivatives. Reagents and conditions: (a) NaH, (CH_3_)_2_CO_3_, THF, reflux, 16 h. (b) (1) Br_2_, CCl_4_, room temperature, 24 h; (2) Na_2_SO_3_, H_2_O, room temperature, 24 h. (c) KOH, CH_3_OH:H_2_O (9:1), reflux, 2 h. (**d**) Ph(BOH)_2_, CsF, Pd(dppf)Cl_2_, CH_3_CN:H_2_O (4:1), 90 °C, 24 h.

Indole analogues were prepared next as a bioisostere of the benzofuran, where the hydrogen bond acceptor of the benzofuran was replaced by a secondary amine (Scheme 4). The amino group of the indole not only had the potential to form additional interactions with residues in the binding groove but also offered a new moiety from which to probe a different region of the binding groove using *N*-alkylation. Indole synthesis was undertaken based on the method of Suárez-Castillo and co-workers who previously regioselectively brominated the 6-position of methyl-3-indole acetate after installing electron withdrawing methyl carbonyl groups at N-1 and the alpha carbon adjacent to C-3 [19]. Using commercially available ethyl 3-indole acetate as the starting material, methyl carbonyl groups were installed using dimethyl carbonate and sodium hydride (Scheme 4). These conditions transesterified the ethyl ester affording **32** as the dimethyl malonate and this group, and in conjunction with the *N*-carbamate group, directed substitution regioselectively to the 6-position in the subsequent bromination step to afford **33**. Decarbomethoxylation was achieved using potassium hydroxide in methanol:water (9:1) to avoid use of toxic sodium cyanide, as described by Suárez-Castillo and co-workers [19], resulting in the production of the 6-bromo indole analogue **34**, albeit at a lower yield. One derivative of indole **34** was synthesised by coupling phenylboronic acid to **33** via Suzuki–Miyaura coupling, which, following the decarbomethoxylation of **35**, afforded the 6-phenyl indole compound **36**. Unfortunately, neither **34** nor **36** appeared to bind in the *Ec*DsbA binding groove as shown by the lack of electron density corresponding to the compounds after X-ray diffraction analysis of the *Ec*DsbA soaked crystals.

**Scheme 5:**
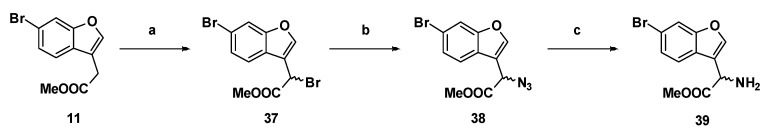
Amination of **11** via bromo and azido intermediates as an amide precursor. Reagents and conditions: (a) NBS, AIBN, CCl_4_, reflux, 16 h; (b) NaN_3_, DMF, −40 °C, 1 h; (c) H_2_, PtO_2_, MeOH.

The co-structures at this point had shown the benzofurans bind to the hydrophobic end of the binding groove and it was considered desirable to develop a benzofuran which could bind to the more polar region of the groove. The next series of analogues focused on elaboration from the α-carbon in an attempt to probe into this region. The α-carbon of **11** was brominated under Wohl–Ziegler conditions to afford bromide **37** (Scheme 5), which in turn was displaced using sodium azide to give the azido compound **38**. Low temperatures were necessary during this reaction to avoid the formation of an α-keto-ester by-product. The azide was then hydrogenated over platinum oxide to produce amine **39**.

A series of amides/sulfonamides were synthesised by coupling amine **39** to the relevant acyl- and sulfonyl chlorides (Table 1). Base hydrolysis of the resulting amides/sulfonamide methyl esters afforded alkyl amide **46**, aromatic amides **47**–**49**, and sulfonamides **50**–**51** in modest to high yields. This series was evaluated for binding and crystallography as racemic mixtures to gauge whether it was worthwhile separating each enantiomer. The impact of the amide on the binding pose of the benzofurans was evaluated by X-ray crystallography. After soaking the amide/sulfonamide series into crystals of *Ec*DsbA, only the (*S*)-enantiomer of sulfonamide **50** produced sufficient electron density in the binding groove. In comparison with the binding pose of the 6-aryl series, the crystal co-structure of **50** showed a 180° horizontal rotation of the benzofuran core and a shift towards the hydrophobic end of the binding groove, resulting in a close proximity of the sulfonamide moiety to His32 (Figure S2). It was not clear if this binding pose was typical for the amides/sulfonamide series, as **50** was the only analogue to produce a crystal structure.

### 2.2. Binding Analysis by NMR Spectroscopy

To validate the binding of benzofuran analogues, three ligand-detected NMR methods, saturation-transfer difference (STD) [20], Carr–Purcell–Meiboom–Gill (CPMG) [21], and water–ligand observed via gradient spectroscopy (waterLOGSY) [22], were employed to detect protein-ligand binding between the benzofurans and *Ec*DsbA (Figure 2C). These experiments were designed as a primary screening tool to select analogues to undergo further affinity evaluation and prioritise compounds for crystal soaking. It has previously been reported that compounds which passed more ligand-detected NMR experiments had a higher chance of obtaining the co-crystal structures [23]. In a good agreement with this finding, eight out of nine benzofurans that passed all three ligand-detected experiments have their co-crystal structures successfully solved; in contrast, five compounds that failed two ligand-detected experiments only have one co-structure solved (Table 2). 

Compounds that passed at least one ligand-detected NMR experiment were further validated by 2D ^1^H-^15^N heteronuclear single quantum coherence (HSQC) spectroscopy. In HSQC experiments, chemical shift perturbations (CSP) of backbone amide resonances of ^15^N-labelled *Ec*DsbA were measured upon addition of compound at 1 mM. If a compound caused CSP > 0.04 ppm for residues present in the hydrophobic groove of DsbA (the threshold was set based on previous experiments) [17], *K*_D_ could be determined by measuring the CSP of those residues induced by different concentrations of compounds (Figure 2D,E). As shown in Table 2, the binding of all compounds was validated by at least one ligand-detected NMR experiment. In accordance with the crystal structure, the majority of the residues perturbed by 1 mM of benzofuran analogues in single-point HSQCs are located around the hydrophobic groove which is an important binding site for DsbA substrates (Figure 2A). Although the co-structures showed π-stacking of the benzofuran analogues with His32, this residue is not assigned in the apo-HSQC spectrum of *Ec*DsbA, and therefore, CSP could not be measured for this residue. Eight compounds produced large enough CSP (>0.04 ppm) to enable HSQC titration and *K*_D_ determination.

*K*_D_ values of benzofuran analogues ranged from 326 to 2000 µM (Table 2). A comparison to fragment hit **6** was not possible as the weak affinity did not allow for accurate determination of its *K*_D_. The ligand efficiency (LE) ranged from 0.15 to 0.24 kcal mol^−1^ HAC^−1^. The strongest binding affinities were observed for 6-phenoxy **25** and 6-benzyl **28** with *K*_D_ values of 326 ± 25 µM and 341 ± 47 µM, respectively. Biophysical characterisation of **25** is illustrated in Figure 2. The similar binding affinities observed for **25** and **28** suggested that the oxygen atom of the ether linker does not contribute to the interaction, which is consistent with what we observed in the co-crystal structures (Figure 1D and Figure 2B). The addition of a *m*-methoxy **26** substituent on the 6-phenoxy group led to a 2-fold decrease in binding affinity, while extending the phenoxy group to a benzyloxy group led to a 2.5-fold decrease. This suggests that **26** may not engage any polar interactions with DsbA despite its close proximity to the polar residues His32 and Gln35. A dramatic decrease was observed for alkyl ether **21** which led to a six-fold decrease relative to **25**. Alkyl substituents at C-6 flipped the compound orientation within the hydrophobic groove and disrupt the polar interaction between the imidazole side chain of His32 and carboxyl group of the compound present in the **25** co-structure (Figure 1C). Similarly, the flipped orientation within the groove also makes **22** a weaker binder, although its binding to DsbA was detected by CPMG spectroscopy, its CSPs are too small to determine *K*_D_ in HSQC. Unfortunately, aniline **15**, which bound in a similar orientation to **25** and **28**, did not produce interpretable data from the HSQC titration due to poor solubility. Of the amine analogues, a *K*_D_ was calculated only for *p*-anisidine analogue **16** which showed a significantly weaker binding affinity. It was evident in the co-crystal structure of *p*-anisidine **16** that the *p*-methoxy group had caused the benzofuran scaffold to shift further along the hydrophobic groove, possibly disrupting the π-stacking interaction with His32 and polar interactions with Gln164 (Figure 1B). LE values showed 6-phenoxy **25** and 6-benzyl **28** were the most efficient binders. Adding functional groups (**16** and **26**) or extending the ring linker (**23**) increased the heavy atom count but did not contribute to binding affinity; therefore, leading to less efficient binders.

## 3. Conclusions

A fragment-based drug discovery process was employed to identify weak binders of *Ec*DsbA and improve the binding affinity through elaboration of the fragment scaffold. A series of analogues were synthesised derivatising the C-6 position to install ether, amino, and benzyl substituents. X-ray crystallography showed the analogues were binding in the hydrophobic groove of *Ec*DsbA, an important site for binding of the cognate redox partner and unfolded substrates. Ligand-detected NMR experiments confirmed the most promising binders, and after further evaluation by HSQC, phenoxy **25** and benzyl **28** analogues were identified as the strongest binders with a *K*_D_ of 326 ± 25 and 341 ± 47 µM respectively. The results presented in this work suggest further opportunity to develop appropriately substituted benzofuran analogues into high affinity DsbA inhibitors.

## 4. Materials and Methods

### 4.1. General

All commercial materials were used as received without further purification, unless otherwise specified. Purification of solvents and reagents, if required, was carried out by procedures described by Chai and Armarego [24]. Moisture sensitive reactions were performed under an atmosphere of nitrogen with all reactions carried out at room temperature, unless otherwise noted. Glassware was oven-dried and cooled under nitrogen prior to use. Analytical thin layer chromatography (TLC) was performed on Merck Kieselgel (Darmstadt, Germany) 60 F254 aluminium backed plates and visualised using a 254 nm UV lamp. Flash chromatography was performed on silica gel (Davisil^®^ LC60Å 40–63 micron, Columbia, SC, USA). Melting points were determined on a Reichert (Buffalo, NY, USA) ‘Thermopan’ microscope hot stage apparatus and values were corrected by a 12% increase after calibration against known reference samples. Low-resolution electrospray ionisation (ESI) mass spectra were recorded on a Bruker Daltronics Esquire 6000 Ion Trap mass spectrometer (Billerica, MA, USA) in methanol or acetonitrile (0.1% formic acid for positive mode) at 300 °C, with a 40 eV cone voltage, and a scan rate of 5500 *m*/*z*/*s*. High-resolution electrospray ionisation (ESI) mass spectrometry was carried out using an Agilent Technologies (Santa Clara, CA, USA) Accurate Mass Q-TOF LC-MS 6530 using Autosampler 1260 Infinity II in positive mode. The samples were analysed using a flow rate of 1 mL/min, a mass range of 100–1,000 *m*/*z* and a scan rate of 10,000 *m*/*z*/*s*. Analytical RP-HPLC was performed on a Shimadzu (Kyoto, Japan) LC-20AB Prominence Liquid Chromatography system fitted with a Phenomenex^®^ Jupiter C18 300 Å column (250 mm × 4.6 mm, 10 μm), using a buffered binary system; solvent A: 0.1% trifluoroacetic acid; solvent B: acetonitrile. Gradient elution was performed using a gradient of 90% solvent A to 90% solvent B over 20 min with a flow rate of 1 mL/min, monitored at 254 nm. Semi-preparative RP-HPLC was performed using a Phenomenex Jupiter (Torrance, CA, USA) C18 column with the same binary buffer system described for RP-HPLC over 60 min with a flow rate of 2 mL/min, unless otherwise stated. The purity of biological tested compounds was >95% in all cases, unless specified otherwise. Microwave assisted reactions were performed using a Milestone StartSYNTH system. NMR spectra were recorded on a Bruker AV-400 and AV-500 spectrometer at 400.13 and 500.02 MHz for ^13^C respectively, and for ^1^H nuclei at 100.62 and 125.74 MHz respectively (^13^C nuclei at 300 K). For ^1^H-NMR the residual CDCl_3_ peak (7.26 ppm), DMSO-*d*_6_ peak (2.50 ppm), CD_3_OD peak (3.31 ppm), or acetone-*d*_6_ peak (2.05 ppm) were used as internal standards. Similarly, ^13^C-NMR spectra were referenced to the residual solvent; the central peak of the CDCl_3_ ‘triplet’ (77.0 ppm), DMSO-*d*_6_ ‘heptet’ (40.0 ppm), CD_3_OD ‘heptet’ (47.6), or acetone-*d*_6_ ‘singlet’ (206.4 ppm). Chemical shifts were reported as δ values in parts per million (ppm). The following abbreviations have been used upon reporting spectral data: s, singlet; d, doublet; t, triplet; q, quartet; m, multiplet; and br, broad.

### 4.2. Chemical Syntheses

General Procedure A: Base mediated ester hydrolysis. Esters were hydrolysed according to the method of Theodorou and co-workers [25]. Esters were dissolved in a mixture of methanol:dichloromethane (1:9) and treated with a 2 M methanolic solution of sodium hydroxide (1–5 equiv) to give a final 0.1 M concentration of ester. The solution was stirred for 3–20 h at which time a cloudy suspension had formed. The suspension was concentrated under reduced pressure and the residue was dissolved in water. The solution was acidified with concentrated HCl to pH 1 then the carboxylic acids were extracted with dichloromethane or ethyl acetate (×3). The combined organic layers were dried over magnesium sulfate then concentrated under reduced pressure. Unless otherwise stated, no purification was required. 

General Procedure B: Preparation of amines via Buchwald–Hartwig coupling. *t*-Butyl alcohol (25 mL/mmol) was degassed by bubbling nitrogen for 20 min. The aryl bromide, amine (1.2 equiv) and potassium carbonate (2.3 equiv) were added and the reaction vessel was evacuated and refilled with nitrogen. This was repeated twice more before addition of XPhos (10 mol%) and Pd_2_(dba)_3_ (5 mol%). The reaction vessel was again purged thrice more before stirring at reflux under nitrogen for 16 h. The reaction mixture was partitioned between water and dichloromethane. The aqueous phase was extracted with dichloromethane (×3) then the combined organic phases were washed with brine (×3). The dichloromethane was removed under reduced pressure and the crude amine was purified by flash chromatography.

General Procedure C: Preparation of ethers/esters via Williamson ether synthesis. Compound 9, anhydrous potassium carbonate (3.3 equiv) and alkyl bromide (4.6 equiv) in dry acetone or acetonitrile were refluxed under an atmosphere of nitrogen for 16 h. The reaction was cooled to room temperature and concentrated to a residue which was partitioned between ethyl acetate and brine. The aqueous phase was washed twice with ethyl acetate and the combined organic phases were dried over magnesium sulfate. The solution was concentrated under reduced pressure to afford the product that was purified, if necessary, by flash chromatography.

General Procedure D: Preparation of ethers via Ullmann-type coupling. Compound 9 or 10, phenol (1.2 equiv), tri-potassium phosphate (3 equiv), copper(I) iodide (10 mol%), and picolinic acid (20 mol%) were added to an oven-dried Schlenk tube charged with a stir bar. Dry dimethyl sulfoxide was added via syringe under a positive pressure of nitrogen. The tube was purged with nitrogen (×3), then sealed and heated to 90 °C causing a colour change from green to brown. The reaction was stirred until analytical RP-HPLC indicated no starting material (24 h), and then, was partitioned between ethyl acetate and water. The aqueous phase was acidified with concentrated hydrochloric acid and was extracted with ethyl acetate (×3). The combined organic phases were dried over magnesium sulfate then concentrated under reduced pressure to give the crude compound. Purification was carried out by flash chromatography, semi-preparative RP-HPLC or recrystallization to afford the pure products. 

General Procedure F: Preparation of amides via coupling of amines to acid/sulfonyl chlorides. A solution of amine 39 and triethylamine (3 equiv) in dichloromethane was treated with acid chloride (1.1 equiv) or sulfonyl chloride (1.1 equiv). The solution was refluxed under nitrogen until complete consumption of starting material by TLC (5–16 h). The reaction was quenched with sodium hydrogen carbonate then extracted with dichloromethane (×3). The organic phase was washed with brine (×3), and then concentrated by rotary evaporation to yield the amide. Purification by flash chromatography was undertaken if required. 

General Procedure G: Preparation of amides via in-situ formation of acid/sulfonyl chloride and subsequent coupling to amine. A solution of carboxylic acid (1–1.2 equiv) or sulfonic acid (1–1.2 equiv) in dry dichloromethane (30 mL/mmol) was treated with phosphorous pentachloride (1.0–1.2 equiv) and refluxed for 30 min under nitrogen. After cooling to room temperature, a solution of amine 39 and triethylamine (3 equiv) in dry dichloromethane (6 mL/mmol) was added. The resulting solution was refluxed until complete consumption of the amine as determined by TLC (2–5 h). The reaction was quenched with sodium hydrogen carbonate then extracted with dichloromethane (×3). The organic phase was washed with brine (×3) then concentrated by rotary evaporation to yield the amide, which was purified by flash chromatography if required.

*7-Bromo-4-chloromethylcoumarin* (**1**): 3-Bromophenol (5.00 g, 28.9 mmol) was added to chilled 70% sulfuric acid (30 mL) and the stirring solution was cooled in an ice bath to less than 5 °C. Ethyl 4-chloroacetoacetate (3.90 mL) was added dropwise ensuring the internal temperature remained below 5 °C. The solution was stirred for two hours and was then warmed to room temperature and stirred for 22 h. The resulting suspension was poured onto ice water (500 mL) and the off-white precipitate was collected by vacuum filtration and recrystallised from ethanol:ethyl acetate (1:1) to afford the compound in question as white needles (2.04 g, 26%); mp 242–244 °C. δ_H_ (500 MHz, CDCl_3_) 7.56 (1H, d, *J* = 2.0 Hz, ArH), 7.54 (1H, d, *J* = 8.5 Hz, ArH), 7.47 (1H, dd, *J* = 7.0 Hz, 1.5, ArH), 6.58 (1H, s, CH), 4.64 (2H, s, CH_2_). δ_C_ (125 MHz, CDCl_3_) 159.4 (C_q_), 154.1 (C_q_), 149.0 (C_q_), 127.9, 126.3 (C_q_), 125.3, 120.7, 116.3, 116.1, 41.0 (CH_2_). LRMS (ESI): *m*/*z* 272.97 [M (^79^Br) + H]^+^, 274.96 [M (^81^Br) + H]^+^. HRMS (ESI): 294.9137 [M (^79^Br) + Na]^+^, 296.9117 [M (^81^Br) + H]^+^.

*6-Bromo-4-chloromethylcoumarin* (**2**)*:* 4-Bromophenol (4.00 g, 23.1 mmol) was dissolved in methanesulfonic acid (21 mL) and cooled in an ice bath to 2 °C. Ethyl 4-chloroacetoacetate (3.12 mL) was added dropwise ensuring the temperature of the solution did not exceed 5 °C. The dark red solution stirred at 0 °C for 2 h then at room temperature for one week. The reaction mixture was poured onto ice then extracted with dichloromethane. The organic phase was washed with 1 M sodium hydroxide (×2) to remove the unreacted phenol; then the organic phase was dried (MgSO_4_) and concentrated to a light yellow semi-solid. Trituration with diethyl ether afforded the pure product as a light orange solid (1.66 g, 26%); mp 164–166 °C. δ_H_ (400 MHz, CDCl_3_) 7.79 (1H, s, ArH), 7.66 (1H, d, *J* = 8.8 Hz, ArH), 7.27 (1H, d, *J* = 8.8 Hz, ArH), 6.61 (1H, s, CH), 4.64 (2H, s, CH_2_). δ_C_ (100 MHz, CDCl_3_) 159.4 (C_q_), 152.8 (C_q_), 148.4 (C_q_), 135.1, 126.8, 119.2, 118.9 (C_q_), 117.3 (C_q_), 116.9 (CH), 40.9 (CH_2_). LRMS (ESI): *m*/*z* 276.9 [M (^79^Br) + H]^+^, 278.9 [M (^81^Br) + H]^+^. HRMS (ESI): 296.9128 [M (^79^Br) + Na]^+^, 296.9099 [M (^81^Br) + Na]^+^.

*4-(Chloromethyl)-7-iodo-2H-chromen-2-one* (**3**): 3-Iodophenol (1.00 g, 4.55 mmol) was dissolved in methanesulfonic acid (5 mL) and cooled in an ice bath to 15 °C. Ethyl 4-chloroacetoacetate (619 µL, 4.55 mmol) was added drop-wise at a slow enough rate to keep the reaction mixture below 10 °C. The reaction stirred for one hour before being removed from the ice bath. After 40 h of stirring at room temperature the purple suspension was poured onto ice and the resulting precipitate was collected by vacuum filtration. The crude off-white solid was recrystallised from ethanol:ethyl acetate (1:1) to afford the compound in question as a white solid (554 mg, 38%); mp 215–217 °C. δ_H_ (400 MHz, CDCl_3_) 7.76 (1H, s, ArH), 7.67 (1H, d, *J* = 8.4 Hz, ArH), 7.37 (1H, d, *J* = 8.0 Hz, ArH), 6.59 (1H, s, CH), 4.63 (2H, s, CH_2_). δ_C_ (100 MHz, CDCl_3_) 159.3, 153.7, 149.1, 133.7, 126.6, 125.1, 116.8, 116.4, 97.9, 41.0. LRMS (ESI): *m*/*z* 320.90 [M + H]^+^.

*7-Hydroxy-4-chloromethylcoumarin* (**4**): To a chilled solution of resorcinol (5.00 g, 45.4 mmol) in 70% H_2_SO_4_ (50 mL) was added, dropwise, ethyl 4-chloroacetoacetate (6.15 mL), while ensuring the temperature did not exceed 5 °C. The chilled solution stirred for two hours, then 20 h at room temperature. The reaction mixture was poured onto ice and the precipitate was collected by vacuum filtration. The light pink semi-solid was triturated with diethyl ether then recrystallised from ethanol to give the compound in question as a white solid (5.18 g, 57%); mp. 178–180 °C (lit. 185 °C) [26]. The mother liquor was concentrated and purified by flash chromatography (20–40% ethyl acetate in hexanes) to afford the compound in question as a white solid (888 mg, 9%). δ_H_ (500 MHz, DMSO-*d*_6_) 10.63 (1H, bs, s, OH), 7.67 (1H, d, *J* = 8.5 Hz, ArH), 6.83 (1H, d, *J* = 8.5 Hz, ArH), 6.74 (1H, s, ArH), 6.41 (1H, s, ArH), 4.94 (1H, s, CH_2_). δ_C_ (100 MHz, DMSO-*d*_6_) 161.9, 160.6, 155.8, 151.5, 127.0, 113.6, 111.5, 109.8, 103.0, 41.8.

*6-Hydroxy-4-chloromethylcoumarin* (**5**): Hydroquinone (1.60 g, 14.7 mmol) was dissolved in 73% sulfuric acid (10 mL) by heating. The solution was cooled to 4 °C in an ice bath and ethyl 4-chloroacetoacetate (1.99 mL) was added dropwise ensuring the internal temperature remained below 5 °C. The dark red solution stirred in an ice bath for one hour then at room temperature for three days. The reaction was poured onto ice and the resulting light brown solid was collected by vacuum filtration. Recrystallisation from 1,4-dioxane afforded the compound in question as a light brown solid (100 mg, 3%); mp 220–222 °C (lit. 220–222 °C) [27]. δ_H_ (500 MHz, DMSO-*d*_6_) 9.83 (1H, br s, OH), 7.28 (1H, d, *J* = 9.0 Hz, ArH), 7.13 (1H, s, ArH), 7.07 (1H, d, *J* = 8.8 Hz, ArH), 6.64 (1H, s, CH), 5.00 (2H, s, CH_2_). δ_C_ (100 MHz, DMSO-*d*_6_) 160.3 (C_q_), 154.2 (C_q_), 150.8 (C_q_), 147.1 (C_q_), 120.7, 118.2, 118.1 (C_q_), 116.1, 110.1 (CH), 41.8 (CH_2_).

*2-(6-Bromobenzofufran-3-yl)acetic acid* (**6**): Coumarin **1** (1.77 g, 6.48 mmol) was suspended in 2 M NaOH (37 mL) and stirred at 80 °C for 18 h. The resulting suspension was cooled to room temperature and acidified with concentrated hydrochloric acid to pH 1. The white precipitate was collected by vacuum filtration and dried under reduced pressure (1.63 g, 99%); mp 154–156 °C (lit. 156–157 °C) [28]. δ_H_ (500 MHz, DMSO-*d*_6_) 7.90 (1H, s, ArH), 7.86 (1H, s, ArH), 7.56 (1H, d, *J* = 8.0 Hz, ArH), 7.42 (1H, d, *J* = 8.25 Hz, ArH), 3.68 (2H, s, CH_2_). δ_C_ (100 MHz, DMSO-*d*_6_) 172.2 (C_q_), 155.4 (C_q_), 144.8, 127.7 (C_q_), 126.2, 122.3, 117.3 (C_q_), 114.9, 114.7 (C_q_), 29.3 (CH_2_).

*2-(5-Bromobenzofuran-3-yl)acetic acid* (**7**): Coumarin **2** (252 mg, 0.920 mmol) was dissolved in 2 M NaOH (5.5 mL) and stirred at 80 °C for 16 h. TLC indicated complete consumption of the starting material. The solution was cooled to room temperature and acidified with concentrated hydrochloric acid. The product precipitated and was collected by vacuum filtration as a light grey solid (203 mg, 86%); mp 142–143 °C (lit. 142–143 °C) [28]. δ_H_ (400 MHz, DMSO-*d*_6_) 7.93 (1H, s, ArH), 7.81 (1H, s, ArH), 7.55 (1H, d, *J* = 8.8 Hz, ArH), 7.44 (1H, d, *J* = 8.6 Hz, ArH), 3.70 (2H, s, CH_2_). δ_C_ (100 MHz, CDCl_3_) 176.2 (C_q_), 154.0 (C_q_), 144.3, 129.4 (C_q_), 127.6, 122.4, 116.0 (C_q_), 113.1, 112.0 (C_q_), 29.2 (CH_2_). LRMS (ESI): *m*/*z* 253.5 [M (^79^Br) + H]^+^, 254.9 [M (^81^Br) + H]^+^. HRMS (ESI): 252.9521 [M (^79^Br) + H]^+^, 254.9509 [M (^81^Br) + H]^+^.

*2-(6-Iodobenzofuran-3-yl)acetic acid* (**8**): Coumarin **3** (480 mg, 1.50 mmol) was suspended in 2 M NaOH (10 mL) and stirred at 80 °C for 16 h. The resulting solution was cooled to room temperature and acidified with concentrated hydrochloric acid to pH 1. The white precipitate was collected by vacuum filtration and dried under reduced pressure (423 mg, 94%); mp 164–166 °C. δ_H_ (400 MHz, CDCl_3_) 7.86 (1H, s, ArH), 7.57–7.56 (2H, m, 2H), 7.31 (1H, d, *J* = 8.0 Hz, ArH), 3.73 (2H, s, CH_2_). δ_C_ (100 MHz, DMSO-*d*_6_) 172.2, 155.6, 144.3, 131,8, 128.0, 122.6, 120.5, 114.6, 89.0, 29.3. LRMS (ESI): *m*/*z* 300.80 [M − H]^−^. HRMS (ESI): 300.9397 [M − H]^−^.

*2-(6-Hydroxybenzofuran-3-yl)acetic acid* (**9**): A suspension of coumarin **4** (200 mg, 0.950 mmol) in 2 M sodium hydroxide (10 mL) was stirred at 80 °C for 16 h. The resulting brown solution was cooled to room temperature and acidified with concentrated hydrochloric acid to pH 1. The compound in question precipitated as a brown solid which was collected by vacuum filtration (183 mg, 66%); mp 141–143 °C (lit. 142–143 °C) [28]. δ_H_ (500 MHz, DMSO-*d*_6_) 9.49 (1H, br s, OH), 7.65 (1H, s, ArH), 7.33 (1H, d, *J* = 8.4 Hz, ArH), 6.87 (1H, s, ArH), 6.73 (1H, d, *J* = 8.4 Hz, ArH), 3.59 (2H, s, CH2). δ_C_ (125 MHz, DMSO-*d*_6_) 172.5 (C_q_), 156.2 (C_q_), 156.1 (C_q_), 142.0, 120.6, 120.3 (C_q_), 114.3 (C_q_), 112.4, 98.1, 29.6 (CH_2_). LRMS (ESI): *m*/*z* 191.0 [M − H]^−^.

*2-(5-Hydroxybenzofufran-3-yl)acetic acid* (**10**): Coumarin **5** (93.3 mg, 0.4458 mmol) was suspended in 2 M sodium hydroxide (3 mL) and the resulting brown solution was stirred at 80 °C for 48 h. The solution was cooled to room temperature and acidified with concentrated hydrochloric acid to pH 1, then extracted with ethyl acetate (×3). The combined organic phases were dried over magnesium sulfate, then concentrated under reduced pressure to a light brown oil which crystallised to an off-white solid (76.5 mg, 89%); mp 128–130 °C. δ_H_ (400 MHz, DMSO-*d*_6_) 9.14 (1H, br s, OH), 7.75 (1H, s, ArH), 7.31 (1H, d, *J* = 8.8 Hz, ArH), 6.86 (1H, s, ArH), 6.72 (1H, d, *J* = 8.8 Hz, ArH), 3.58 (2H, s, CH_2_). LRMS (ESI): *m*/*z* 191. 0 [M − H]^−^.

*Methyl 2-(6-bromobenzofuran-3-yl)acetate* (**11**): Thionyl chloride (1.31 mL, 18.1 mmol) was added dropwise to chilled methanol (6 mL) and the solution was stirred in an ice bath. After one hour, **6** (1.54 g, 4.02 mmol) was added and the suspension was refluxed for 16 h. Upon cooling to room temperature, white crystals precipitated. The methanol was removed under reduced pressure and the resulting crude solid was purified by flash chromatography (30–70% ethyl acetate in hexanes) to afford the product as an oil, which crystallised to an off-white solid under reduced pressure (1.57 g, 97%); mp 70–71 °C. δ_H_ (400 MHz, CDCl_3_) 7.66 (1H, s, ArH), 7.61 (1H, s, ArH), 7.43 (1H, d, *J* = 8.0 Hz, ArH), 7.38 (1H, d, *J* = 8.0 Hz, ArH), 3.73 (3H, s, CH_3_), 3.69 (2H, s, CH_2_). δ_C_ (100 MHz, CDCl_3_) 170.8, 155.5, 143.4, 126.7, 126.1, 120.7, 117.9, 115.0, 113.1, 52.2, 29.4. HRMS (ESI): 304.9408, 306.9388 [M + K]^+^.

*Methyl 2-(6-iodobenzofuran-3-yl)acetate* (**12**): Thionyl chloride (932 µL) was added dropwise to chilled methanol (10 mL) and the solution was stirred for 50 min under nitrogen. The acid **8** (1.29 g, 4.28 mmol) was added and the suspension was heated to reflux for four hours. Upon cooling to room temperature, a precipitate formed. The suspension was chilled in an ice bath and water was added, causing more solid to precipitate. The precipitate was collected by vacuum filtration to give the compound in question as an off-white solid (945 mg, 70%); mp 80–82 °C. δ_H_ (400 MHz, CDCl_3_) 7.85 (1H, s, ArH), 7.57–7.54 (2H, m, ArH), 7.31 (1H, d, *J* = 8.2 Hz, ArH), 3.73 (3H, s, OCH_3_), 3.68 (2H, s, CH_2_). δ_C_ (100 MHz, CDCl_3_) 170.8 (C_q_), 155.7 (C_q_), 143.2, 131.7, 127.3 (C_q_), 121.1, 120.8, 113.1 (C_q_), 88.1 (C_q_), 52.2 (OCH_3_), 29.4 (CH_2_). LRMS (ESI): *m*/*z* 339.0 [M + Na]^+^. HRMS (ESI): 296.9860 [M − H_2_O − H]^−^.

*Methyl 2-(6-(phenylamino)benzofuran-3-yl)acetate* (**13**): Compound **11** (250 mg, 0.929 mmol) was coupled to aniline (102.0 µL) according to General Procedure B. The crude orange oil was purified by flash chromatography (5–10% ethyl acetate in hexanes) to afford the compound in question as an orange oil (178 mg, 68%). δ_H_ (400 MHz, CDCl_3_) 7.52 (1H, s, ArH), 7.42 (1H, d, *J* = 8.4 Hz, ArH), 7.30–7.24 (3H, m, ArH), 7.08 (2H, d, *J* = 8.5 Hz, ArH), 6.99 (1H, d, *J* = 8.4 Hz, ArH), 6.93 (1H, t, *J* = 7.4 Hz, ArH), 3.74 (3H, s, OCH_3_), 3.68 (2H, s, CH_2_). δ_C_ (125 MHz, CDCl_3_) 171.2 (C_q_), 156.3 (C_q_), 143.5 (C_q_), 141.9, 140.9 (C_q_), 129.4, 121.8 (C_q_), 120.9, 120.0, 117.5, 115.3, 113.0 (C_q_), 100.9, 52.1 (CH_3_), 29.6 (CH_2_). LRMS (ESI): *m*/*z* 282.0 [M + H]^+^.

*Methyl 2-(6-((4-methoxyphenyl)amino)benzofuran-3-yl)acetate* (**14**): Compound **11** (100 mg, 0.372 mmol) was coupled to *p*-anisidine (54.9 mg) according to General Procedure B. The crude dark orange oil was purified by flash chromatography (dichloromethane) to afford the compound in question as a yellow oil (57.5 mg, 50%). δ_H_ (400 MHz, CDCl_3_) 7.47 (1H, s, ArH), 7.37 (1H, d, *J* = 8.4 Hz, ArH), 7.09 (2H, d, *J* = 8.9 Hz, ArH), 7.04 (1H, s, ArH), 6.89–6.83 (3H, m, ArH), 5.61 (1H, br s, NH), 3.81 (3H, s, OCH_3_), 3.73 (3H, s, OCH_3_), 3.66 (2H, s, CH_2_). δ_C_ (100 MHz, CDCl_3_) 171.2 (C_q_), 156.6 (C_q_), 155.2 (C_q_), 143.2 (C_q_), 141.3, 136.0 (C_q_), 121.9, 120.5 (C_q_), 119.9, 114.7, 113.3, 112.9 (C_q_), 98.2, 55.5 (OCH_3_), 52.1 (OCH_3_), 29.6 (CH_2_). LRMS (ESI): *m*/*z* 312.1 [M + H]^+^.

*2-(6-(Phenylamino)benzofuran-3-yl)acetic acid* (**15**): Ester **13** (178 mg, 0.634 mmol) was hydrolysed using 2 M sodium hydroxide according to General Procedure A to afford the compound in question as a brown solid (160 mg, 94%); mp 127–130 °C. δ_H_ (400 MHz, CDCl_3_) 7.53 (1H, s, ArH), 7.43 (1H, d, *J* = 8.4 Hz, ArH), 7.30–7.25 (3H, m, ArH), 7.09 (2H, d, *J* = 8.4 Hz, ArH), 6.99 (1H, d, *J* = 8.4 Hz, ArH), 6.95 (1H, t, *J* = 7.4 Hz, ArH), 3.72 (2H, s, CH2). δ_C_ (125 MHz, CDCl_3_) 176.5 (C_q_), 156.3 (C_q_), 143.4 (C_q_), 142.1, 141.0 (C_q_), 129.4, 121.6 (C_q_), 121.0, 120.0, 117.6, 115.3, 112.4 (C_q_), 100.8, 29.5 (CH_2_). LRMS (ESI): *m*/*z* 265.96 [M − H]^−^. HRMS (ESI): 306.1621 [M + K]^+^.

*2-(6-((4-Methoxyphenyl)amino)benzofuran-3-yl)acetic acid* (**16**): Ester **14** (50.0 mg, 0.161 mmol) was hydrolysed using 2 M methanolic sodium hydroxide (5 equiv, 402 µL) according to General Procedure A to afford the compound in question as a light brown solid (47.9 mg, 100%); mp 120–122 °C. δ_H_ (400 MHz, CDCl_3_) 7.49 (1H, s, ArH), 7.38 (1H, d, *J* = 8.4 Hz, ArH), 7.13 (3H, m, ArH), 6.93 (1H, d, *J* = 8.4 Hz, ArH), 6.89–6.83 (3H, m, ArH), 3.81 (3H, s, OCH_3_), 3.70 (2H, s, CH_2_). δ_C_ (100 MHz, CDCl_3_) 176.2 (C_q_), 156.6 (C_q_), 155.4 (C_q_), 143.4 (C_q_), 141.6, 135.9 (C_q_), 122.1, 120.4 (C_q_), 120.0, 114.8, 113.4, 112.3 (C_q_), 98.3, 55.6 (OCH_3_), 29.5 (CH_2_). LRMS (ESI): *m*/*z* 296.1 [M − H]^−^. HRMS (ESI): 296.0959 [M − H]^−^.

*Butyl 2-(6-butoxybenzofuran-3-yl)acetate* (**17**): Compound **9** (200 mg. 1.041 mmol) was reacted with *n*-butyl bromide according to General Procedure C using acetonitrile as solvent, to give the compound in question as a yellow oil (205 mg, 65%) which did not require purification. δ_H_ (500 MHz, CDCl_3_) 7.52 (1H, s, ArH), 7.41 (1H, d, *J* = 9.0 Hz, ArH), 6.99 (1H, s, ArH), 6.88 (1H, d, *J* = 8.5 Hz, ArH), 4.13 (2H, t, *J* = 6.8 Hz, OCH_2_), 4.00 (2H, t, *J* = 6.5 Hz), OCH_2_), 3.65 (2H, s, CH_2_), 1.79 (2H, m, CH_2_), 1.61 (2H, m, CH_2_), 1.53 (2H, m, CH_2_), 1.35 (2H, m, CH_2_), 0.97 (3H, t, *J* = 7.5 Hz, CH_3_), 0.91 (3H, t, *J* = 7.3 Hz, CH_3_). δ_C_ (100 MHz, CDCl_3_) 170.8 (C_q_), 157.7 (C_q_), 156.2 (C_q_), 141.8, 120.9 (C_q_), 119.7, 113.1 (C_q_), 112.3, 96.8, 68.3 (OCH_2_), 64.9 (OCH_2_), 31.3 (CH_2_), 30.6 (CH_2_), 29.9 (CH_2_), 19.3 (CH_2_), 19.1 (CH_2_), 13.8 (CH_3_), 13.6 (CH_3_). LRMS (ESI): *m*/*z* 305.1 [M + H]^+^.

*Isobutyl 2-(6-isobutoxybenzofuran-3-yl)acetate* (**18**): Compound **9** (200 mg, 1.04 mmol) was reacted with *i*-butyl bromide according to General Procedure C using acetonitrile as solvent, to give the compound in question as a yellow oil (50.0 mg, 16%) which did not require further purification. δ_H_ (400 MHz, CDCl_3_) 7.52 (1H, s, ArH), 7.41 (1H, d, *J* = 8.4 Hz, ArH), 6.99 (1H, s, ArH), 6.89 (1H, d, *J* = 8.4 Hz, ArH), 3.91 (2H, d, *J* = 6.8 Hz, CH_2_), 3.75 (2H, d, *J* = 6.4 Hz, OCH_2_), 3.67 (2H, s, CH_2_), 2.11 (1H, sept, *J* = 6.8 Hz, CH), 1.92 (1H, sept, *J* = 6.8 Hz, CH), 1.04 (6H, d, *J* = 6.4 Hz, 2CH_3_), 0.90 (6H, d, *J* = 6.8 Hz, 2CH_3_). δ_C_ (100 MHz, CDCl_3_) 170.8 (C_q_), 157.8 (C_q_), 156.2 (C_q_), 141.8, 120.9 (C_q_), 119.7, 113.1 (C_q_), 112.3, 96.8, 75.1 (OCH_2_), 71.2 (OCH_2_), 29.9 (CH_2_), 28.3 (CH), 29.7 (CH), 19.3 (CH_3_), 19.0 (CH_3_). LRMS (ESI): *m*/*z* 305.1 [M + H]^+^.

*Benzyl 2-(6-(benzyloxyl)benzofuran-3-yl)acetate* (**19**): Compound **9** (315 mg, 1.64 mmol) was reacted with benzyl bromide according to General Procedure C using acetone as solvent. Purification by flash chromatography (1–10% ethyl acetate in hexanes) afforded the compound in question as a white solid (315 mg, 52%); mp 77–79 °C (lit. 77–78.5 °C) [29]. δ_H_ (400 MHz, CDCl_3_) 7.53 (1H, s, ArH), 7.46–745 (2H, m, ArH), 7.41–7.33 (9H, m, ArH), 7.07, (1H, s, ArH), 6.94 (1H, d, *J* = 8.4 Hz, ArH), 5.16 (2H, s, OCH_2_), 5.11 (2H, s, OCH_2_), 3.71 (2H, s, CH_2_). δ_C_ (100 MHz, CDCl_3_) 170.5, 157.2, 156.1, 142.0, 136.9, 135.6, 128.6, 128.5, 128.3, 128.3, 128.0, 127.5, 121.2, 119.9, 112.8, 112.5, 97.3, 70.6, 66.8, 29.8. LRMS (ESI): *m*/*z* 373.1 [M + H]^+^.

*Benzyl 2-(5-benzyloxy)benzofuran-3-yl)acetate* (**20**): Compound **10** (75.0 mg, 0.390 mmol) was reacted with benzyl bromide (21.3 µL) according to General Procedure C using acetone as solvent. Purification by flash chromatography (1–30% ethyl acetate in hexanes) gave the compound in question as a white solid (44.7 mg, 31%); mp 70–72 °C. δ_H_ (400 MHz, CDCl_3_) 7.60 (1H, s, ArH), 7.46–7.32 (11H, m, ArH), 7.06 (1H, d, *J* = 2.4 Hz, ArH), 7.00 (1H, dd, *J* = 9.0 Hz, 2.6, ArH), 5.16 (2H, s, OCH_2_), 5.01 (2H, s, OCH_2_), 3.72 (2H, s, CH_2_). δ_C_ (100 MHz, CDCl_3_) 170.4 (C_q_), 155.1 (C_q_), 150.3 (C_q_), 143.7, 137.1 (C_q_), 135.6 (C_q_), 128.6, 128.5, 128.4, 128.3, 128.1 (C_q_), 127.9, 127.6, 114.1, 113.0 (C_q_), 112.0, 103.5, 70.8 (CH_2_), 66.9 (CH_2_), 29.9 (CH_2_). LRMS (ESI): *m*/*z* 373.1 [M + H]^+^.

*2-(6-Butoxybenzofuran-3-yl)acetic acid* (**21**): Ester **17** (205 mg, 0.673 mmol) was hydrolysed using 2 M methanolic sodium hydroxide (5 equiv, 1.68 mL) according to General Procedure A to give the compound in question as a white solid (143 mg, 86%); mp 108–109 °C. δ_H_ (500 MHz, CDCl_3_) 7.53 (1H, s, ArH), 7.40 (1H, d, *J* = 8.8 Hz, ArH), 7.00 (1H, s, ArH), 6.89 (1H, d, *J* = 8.4 Hz, ArH), 3.99 (2H, t, *J* = 6.4 Hz, OCH_2_), 3.71 (2H, s, CH_2_), 1.79 (2H, p, *J* = 6.8 Hz, CH_2_), 1.51 (2H, sextet, *J* = 7.6 Hz, CH_2_), 0.99 (3H, t, *J* = 7.4 Hz, CH_3_). δ_C_ (100 MHz, CDCl_3_) 176 (C_q_), 157.8 (C_q_), 156.3 (C_q_), 142.0, 120.6 (C_q_), 119.7, 112.4, 112.3 (C_q_), 96.8, 68.3 (OCH_2_), 31.3 (CH_2_), 29.4 (CH_2_), 19.3 (CH_2_), 13.8 (CH_3_). LRMS (ESI): *m*/*z* 247.2 [M − H]^−^.

*2-(6-Isobutoxybenzofuran-3-yl)acetic acid* (**22**): Ester **18** (50.0 mg, 0.164 mmol) was hydrolysed using 2 M methanolic sodium hydroxide (5 equiv, 411 µL) according to General Procedure A. The crude compound required purification by RP-HPLC. The fractions containing the product were lyophilised to give the compound in question as a white solid (6 mg, 15%); mp 130–132 °C. δ_H_ (400 MHz, CDCl_3_) 7.53 (1H, s, ArH), 7.40 (1H, s, *J* = 8.4 Hz, ArH), 6.99 (1H, s, ArH), 6.90 (1H, d, *J* = 8.6 Hz, ArH), 3.75 (2H, d, *J* = 6.4 Hz, OCH_2_), 3.71 (2H, s, CH_2_), 2.11 (1H, sept, *J* = 6.4 Hz, CH), 1.05 (6H, d, *J* = 6.8 Hz, CH_3_). δ_C_ (100 MHz, CDCl_3_) 157.9 (C_q_), 156.3 (C_q_), 142.0, 120.6 (C_q_), 119.6, 112.4, 112.3 (C_q_), 96.9, 75.1 (OCH_2_), 29.7 (CH_2_), 19.3 (CH_3_). One quaternary carbon not observed. LRMS (ESI): *m*/*z* not found.

*2-(6-((Benzyloxy)benzofuran-3-yl)acetic acid* (**23**): Ester **19** (248 mg, 0.665 mmol) was hydrolysed using 2 M sodium hydroxide (5 equiv, 1.66 mL) according to General Procedure A. The compound in question was afforded after recrystallisation from chloroform as a crystalline white solid (124 mg, 61%); mp 153–155 °C. δ_H_ (500 MHz, CDCl_3_) 7.54 (1H, s, ArH), 7.46–7.32 (7H, m, ArH), 7.08 (1H, s, ArH), 6.98 (1H, d, *J* = 8.5 Hz, ArH), 5.11 (2H, s, OCH_2_), 3.71 (2H, s, CH_2_). δ_C_ (100 MHz, CDCl_3_) 176.3, 157.4, 156.1, 142.2, 136.9, 128.6, 128.0, 127.5, 121.1, 119.8, 112.6, 112.3, 97.5, 70.6, 29.5. LRMS (ESI): *m*/*z* 281.0 [M − H]^−^. HRMS (ESI): 281.0848 [M − H]^−^.

*2-(5-(Benzyloxy)benzofuran-3-yl)acetic acid* (**24**): Ester **20** (28.7 mg, 0.0771 mmol) was hydrolysed using 2 M methanolic sodium hydroxide (5 equiv, 193 µL) according to General Method B to give the compound in question as a white solid (16.9 mg, 78%); mp 147–149 °C. δ_H_ (400 MHz, CDCl_3_) 7.61 (1H, s, ArH), 7.47–7.30 (6H, m, ArH), 7.10 (1H, s, ArH), 7.00 (1H, dd, *J* = 8.8 Hz, 2.0, ArH), 5.10 (2H, s, OCH_2_), 3.71 (2H, s, CH_2_). δ_C_ (100 MHz, CDCl_3_) 176.2 (C_q_), 155.2 (C_q_), 150.3 (C_q_), 143.9, 137.1 (C_q_), 128.6, 127.9, 127.6, 114.1, 112.4 (C_q_), 112.1, 103.6, 70.9 (OCH_2_), 29.4 (CH_2_). One quaternary carbon not observed. LRMS (ESI): *m*/*z* 281.0 [M − H]^−^. HRMS (ESI): 281.0848 [M − H]^−^.

*2-(6-Phenoxybenzofuran-3-yl)acetic acid* (**25**): Compound **8** (302 mg, 1.00 mmol) was coupled to phenol according to General Procedure D. The crude compound was purified by flash chromatography (2.5% methanol in dichloromethane) to give a red solid (61.3 mg) that was recrystallised from acetonitrile to give the compound in question as a white solid (7.1 mg, 3%). The mother liquor was concentrated and purified by semi-preparative RP-HPLC. The fractions containing the product were lyophilised to give the compound in question as a white solid (19.7 mg, 7%); mp 107–109 °C. δ_H_ (400 MHz, CDCl_3_) 7.60 (1H, s, ArH). 7.50 (1H, d, *J* = 8.4 Hz, ArH), 7.36–7.32 (2H, m, ArH), 7.19–7.09 (2H, m, ArH), 7.03–7.00 (3H, m, ArH), 7.74 (2H, s, CH_2_). δ_C_ (100 MHz, CDCl_3_) 176.2 (C_q_), 157.6 (C_q_), 155.8 (C_q_), 155.3 (C_q_), 143.2 (C_q_), 129.8, 123.2, 120.1, 118.6, 115.3, 112.4 (C_q_), 102.6, 29.7 (CH_2_). One carbon signal obscured. LRMS (ESI): *m*/*z* 267.2 [M − H]^−^. HRMS (ESI): 267.0689 [M − H]^−^.

*2-(6-(3-Methoxyphenoxy)benzofuran-3-yl)acetic acid* (**26**): Compound **8** (302 mg, 1.00 mmol) was coupled to 3-methoxyphenol (149 mg) according to General Procedure D. The crude brown oil was purified by flash chromatography (2.5% methanol in dichloromethane) and subsequent recrystallisation from aqueous ethanol afforded the compound in question as an off-white solid (30.0 mg, 10%); mp. 91–93 °C. δ_H_ (400 MHz, CDCl_3_) 7.62 (1H, s, ArH), 7.50 (1H, d, *J* = 8.5 Hz, ArH), 7.22 (1H, t, *J* = 8.5 Hz, ArH), 7.15 (1H, s, ArH), 7.02 (1H, d, *J* = 8.5 Hz, ArH), 7.66 (1H, d, *J* = 8.5 Hz, ArH), 6.61–6.58 (2H, m, ArH), 3.78 (3H, s, OCH_3_), 3.75 (2H, s, CH_2_). LRMS (ESI): *m*/*z* 299.11 [M + H]^+^. HRMS (ESI): 297.0800 [M − H]^−^.

*Methyl 2-(6-benzylbenzofuran-3-yl)acetate* (**27**): A three-necked flask charged with zinc powder (535 mg), lithium chloride (433 mg), and a stir bar was flame dried under reduced pressure. The flask was cooled to room temperature and filled with nitrogen. Dry tetrahydrofuran (5 mL) was added via cannula and the suspension was cooled to 0 °C. Benzyl bromide (486 µL) was added dropwise via syringe and the suspension stirred for 1.5 h. The suspension was then transferred via cannula to an evacuated, flame dried flask containing compound **11** (250 mg, 0.929 mmol), Pd(OAc)_2_ (17.9 mg), and XPhos (35.9 mg). The flask was filled with nitrogen then heated to 50 °C. The resulting black reaction mixture was stirred for 18 h, cooled to room temperature, and then treated with 1 M HCl and allowed to stir for 1 min. The organic phase was collected and the aqueous phase was extracted with ethyl acetate (×3). The combined organic phases were dried over magnesium sulfate then concentrated under reduced pressure to a dark yellow oil. The crude oil was purified by flash chromatography (2.5% ethyl acetate in hexanes) to give the compound in question as a yellow oil (60.5 mg, 35%). δ_H_ (400 MHz, CDCl_3_) 7.57 (1H, s, ArH), 7.46 (1H, d, *J* = 8.1 Hz, ArH), 7.31–7.29 (2H, m, ArH), 7.22–718 (3H, m, ArH), 7.12 (1H, d, *J* = 8.1 Hz, ArH), 4.09 (2H, s, CH_2_), 3.72 (3H, s, OCH_3_), 3.68 (2H, s, CH_2_). δ_C_ (100 MHz, CDCl_3_) 171.1 (C_q_), 155.6 (C_q_), 142.6, 141.1 (C_q_), 138.1 (C_q_), 128.9, 128.4, 126.1, 125.7 (C_q_), 123.9, 119.4, 112.9 (C_q_), 111.7, 52.1 (OCH_3_), 42.0 (CH_2_), 29.5 (CH_2_). LRMS (ESI): *m*/*z* 303.1 [M + Na]^+^.

*2-(6-Benzylbenzofuran-3-yl)acetic acid* (**28**): Ester **27** (60.5 mg, 0.216 mmol) was hydrolysed using 2 M methanolic sodium hydroxide (5 equiv, 540 µL) according to General Procedure A to give the compound in question as a while solid (29.2 mg, 51%); mp 124–127 °C. δ_H_ (400 MHz, CDCl_3_) 7.58 (1H, s, ArH), 7.47 (1H, s, ArH), 7.32–7.28 (3H, m, ArH), 7.23–7.21 (3H, m, ArH), 7.13 (1H, d, *J* = 8.0 Hz, ArH), 4.10 (2H, s, CH_2_), 3.73 (2H, s, CH_2_). δ_C_ (100 MHz, CDCl_3_) 176.9, 155.6 (C_q_), 142.8, 141.1 (C_q_), 138.3 (C_q_), 128.9, 128.5, 126.1, 125.5 (C_q_), 124.0, 199.4, 112.2 (C_q_), 111.8, 42.0 (CH_2_), 29.5 (CH_2_). LRMS (ESI): *m*/*z* 265.0 [M − H]^−^. HRMS (ESI): 265.0895 [M − H]^−^.

*2-(6-Bromobenzofuran-3-yl)acetamide* (**29**): Compound **6** (150 mg, 0.588 mmol) was dissolved in dichloromethane (6 mL) in an atmosphere of nitrogen. Phosphorous pentachloride (122 mg, 0.588 mmol) was added and the solution was heated to reflux for 30 min. The reaction cooled to room temperature and ammonia gas (produced by heating an aqueous solution of ammonium hydroxide to 50 °C) was bubbled through the pale-yellow solution. Within 10 s, a white solid precipitated out of solution. After several minutes, the ammonia gas was removed from the reaction then the dichloromethane was removed under reduced pressure. The white residue was partitioned between ethyl acetate and water. The aqueous phase was washed with ethyl acetate (×2) then the combined organic phases were dried over magnesium sulfate and concentrated under reduced pressure to afford the amide as a white solid (135 mg, 90%); mp 140–143 °C. δ_H_ (400 MHz, CDCl_3_) 7.85–7.84 (2H, m, ArH), 7.57 (1H, d, *J* = 8.4 Hz, ArH), 7.53 (1H, br s, NH), 7.42 (1H, d, *J* = 8.4 Hz, ArH), 6.98 (1H, br s, NH), 3.47 (2H, s, CH_2_). δ_C_ (100 MHz, DMSO-*d*_6_) 171.6 (C_q_), 155.4 (C_q_), 144.5, 127.8 (C_q_), 126.1, 122.3, 117.3 (C_q_), 115.7 (C_q_), 115.0, 30.6 (CH_2_). LRMS (ESI): *m*/*z* 253.9 [M + H]^+^. HRMS (ESI): 275.9617 [M + Na]^+^.

*2-(6-Bromobenzofuran-3-yl)acetonitrile* (**30**): Amide **29** (110 mg, 0.431 mmol) was suspended in dry toluene (4.5 mL) in an atmosphere of nitrogen. Distilled thionyl chloride (47.0 µL) was added and the reaction mixture was heated to reflux. The starting material dissolved upon heating. After 1.5 h, TLC showed a new product had formed and only a small amount of starting material remained. The reaction was cooled to room temperature, and then the toluene and excess thionyl chloride were removed under reduced pressure. The resulting dark orange oil was dissolved in ethyl acetate and washed with a saturated solution of sodium hydrogen carbonate and brine (×2). The organic phase was dried (MgSO_4_) and concentrated to a dark orange oil under reduced pressure. The crude oil was purified by flash chromatography (10% ethyl acetate in hexanes) to afford the nitrile as a dark pink solid (66.9 mg, 66%); mp 88–90 °C. δ_H_ (400 MHz, CDCl_3_) 7.70 (1H, s, ArH), 7.65 (1H, s, ArH), 7.46 (1H, d, *J* = 8.4 Hz, ArH), 7.43 (1H, d, *J* = 8.4 Hz, ArH), 3.75 (2H, s, CH_2_). δ_C_ (100 MHz, CDCl_3_) 155.7(C_q_), 143.2, 126.7, 125.1 (C_q_), 119.9, 118.8 (C_q_), 116.3 (C_q_), 115.4, 110.2 (C_q_), 13.0 (CH_2_). LRMS (ESI): *m*/*z* not found. I.R (KBr) 2254 cm^−1^ (-C≡N).

*5-((6-Bromobenzofuran-3-yl)methyl)-1H-tetrazole* (**31**): Nitrile **30** (57 mg, 0.241 mmol), sodium azide (47.1 mg), and triethylammonium chloride (99.7 mg) were suspended in dry toluene (1 mL) in an atmosphere of nitrogen. The suspension was stirred at reflux for 3 days. The reaction was cooled to room temperature and water was added. The aqueous phase was collected and acidified with concentrated hydrochloric acid. Upon acidification, the compound in question precipitated as an off-white solid which was collected by vacuum filtration (50.2 mg, 75%); mp 184–185 °C. δ_H_ (400 MHz, DMSO-*d*6) 7.98 (1H, s, ArH), 7.90 (1H, s, ArH), 7.67 (1H, d, *J* = 8.4 Hz, ArH), 7.42 (1H, d, *J* = 8.0 Hz, ArH), 4.39 (2H, s, CH2). δ_C_ (100 MHz, DMSO-*d*_6_) 155.5 (C_q_), 144.9, 126.9 (C_q_), 126.4, 121.8, 117.7 (C_q_), 115.3 (C_q_), 115.2, 18.3 (CH_2_). One quaternary carbon was not observed. LRMS (ESI): *m*/*z* 279.0 [M (^79^Br) + H]^+^, 280.9 [M (^81^Br) + H]^+^. HRMS (ESI): 276.9758 [M (^79^Br) − H]^−^, 278.9738 [M (^81^Br) − H]^−^.

*Dimethyl 2-(1-(methoxycarbonyl)-1H-indole-3-yl)malonate* (**32**): Based on the method of Suarez–Castillo [19]. A solution of ethyl-3-indole acetate (1.5 g, 7.38 mmol) in dry tetrahydrofuran (10 mL) was added to a suspension of sodium hydride (1.18 g as 60% dispersion in mineral oil, 29.5 mmol) in dry tetrahydrofuran (40 mL) under an atmosphere of nitrogen. The suspension was stirred until the formation of gas was no longer observed (5–10 min). To the resulting green suspension was added dimethyl carbonate (12.0 mL, 142 mmol) and the reaction mixture was stirred at reflux for 16 h, after which time the colour became maroon. The reaction was cooled to room temperature and the tetrahydrofuran was removed under reduced pressure. The resulting residue was partitioned between a saturated solution of sodium hydrogen carbonate and diethyl ether. The aqueous phase was washed with diethyl ether (×2) and the combined organic phases were washed with brine (×3), dried over magnesium sulfate, and concentrated to a red oil by rotary evaporation. A small portion of ether was added and heated to boil. Upon cooling, a solid precipitated which was recrystallised from diethyl ether and the mother liquor was purified by flash chromatography (dichloromethane) to afford the titled product as an off-white solid (1.33 g, 59%); mp 108–110 °C (lit. 108–110 °C) [30]. δ_H_ (500 MHz, CDCl_3_) 8.20 (1H, d, *J* = 7.5 Hz, ArH), 7.82 (1H, s, ArH), 7.57 (1H, d, *J* = 7.5 Hz, ArH), 7.36 (1H, t, *J* = 7.5 Hz, ArH), 7.28 (1H, t, *J* = 7.5, ArH), 4.90 (1H, s, CH), 4.04 (3H, s, OCH_3_), 3.78 (6H, s, (OCH_3_)_2_). δ_C_ (100 MHz, CDCl_3_) 168.0 (C_q_), 151.2 (C_q_), 135.3 (C_q_), 129.1 (C_q_), 125.1, 125.0, 123.2, 119.2, 115.3, 112.8 (C_q_), 53.9 (OCH_3_), 53.0 (OCH_3_), 49.0 (CH).

*Dimethyl 2-(6-bromo-1-(methoxycarbonyl)-1H-indole-3-yl)malonate* (**33**): According to the method of Suarez–Castillo [19]. Malonate **32** (1.25 g, 4.09 mmol) was dissolved in carbon tetrachloride (50.0 mL), treated with bromine (1.68 mL), and stirred at room temperature for 24 h. A saturated solution of sodium sulfite (50 mL) was added and stirred vigorously for 24 h. The aqueous phase was extracted with dichloromethane (×2) and the combined organic phases were washed with brine (×3), dried over magnesium sulfate, and concentrated under reduced pressure to an orange oil. Diethyl ether (15 mL) was added and the solution was cooled until a precipitate formed which was collected and recrystallised from diethyl ether to afford the titled product as an off-white solid (826 mg, 52%). The mother liquor was purified by flash chromatography (5–20% ethyl acetate in hexanes) to afford an off-white solid after trituration with diethyl ether (157 mg, 10%); mp 110–112 °C (lit. 110–112 °C) [19]. δ_H_ (400 MHz, CDCl_3_) 8.38 (1H, br s, ArH), 7.79 (1H, s, ArH), 7.45 (1H, d, *J* = 8.4 Hz, ArH), 7.40 (1H, d, *J* = 8.4 Hz, ArH), 4.85 (1H, s, CH), 4.04 (3H, s, NCOOCH_3_), 3.78 (6H, s, (COOCH_3_)_2_). δ_C_ (100 MHz, CDCl_3_) 167.8 (C_q_), 150.8 (C_q_), 135.9 (C_q_), 127.9, 126.5, 125.6 (C_q_), 120.6, 118.8 (C_q_), 118.4, 112.7 (C_q_), 54.1 (CH_3_), 53.1 (CH_3_), 49.0 (CH).

*2-(6-Bromo-1H-indol-3-yl)acetic acid* (**34**): Malonate **33** (200 mg, 0.640 mmol) was dissolved in methanol:water (9:1) and treated with potassium hydroxide (180 mg, 3.21 mmol) and refluxed for 18 h. The reaction was cooled to room temperature and concentrated under reduced pressure. The resulting residue was dissolved in water and the aqueous solution was washed with ethyl acetate. The aqueous phase was then acidified with 1 M hydrochloric acid to pH 2 and the cloudy suspension was extracted with ethyl acetate (×3). The combined organic layers were dried over magnesium sulfate, then concentrated under reduced pressure to a light yellow solid. Recrystallisation from chloroform afforded the titled product as a white solid (64.0 mg, 39%); mp 178–180 °C δ_H_ (400 MHz, DMSO-*d*_6_) 11.04 (1H, br s, NH/COOH), 7.52 (1H, s, ArH), 7.44 (1H, d, *J* = 8.4 Hz, ArH), 7.25 (1H, s, ArH), 7.10 (1H, d, *J* = 8.4 Hz, ArH), 3.62 (2H, s, CH2). δ_C_ (100 MHz, DMSO-*d*_6_) 173.4, 137.4, 126.8, 125.6, 121.8, 120.9, 114.4, 114.3, 108.6, 31.3. LRMS (ESI): *m*/*z* 252.3 [M (^79^Br) − H)^−^, 253.9 [M (^81^Br) − H]^−^.

*Dimethyl 2-(1-(methoxycarbonyl)-6-phenyl-1H-indol-3-yl)malonate* (**35**): Bromide **33** (250 mg, 0.651 mmol), phenylboronic acid (476 mg), caesium fluoride (297 mg), and Pd(dppf)Cl_2_ (24.0 mg) were dissolved in degassed acetonitrile:water (4:1) in an atmosphere of nitrogen. The suspension was heated to 90 °C and stirred for 18 h. The reaction was partitioned between ethyl acetate and brine and the organic layer was separated, washed with brine (×2), and dried over magnesium sulfate. The ethyl acetate was removed under reduced pressure then the crude brown solid was purified by flash chromatography (20% ethyl acetate in hexane, then 2.5–5% methanol in dichloromethane). The fractions containing the product were concentrated under reduced pressure and triturated with diethyl ether to afford the compound in question as a white solid (30.0 mg, 12%); mp 124–126 °C. *δ*_H_ (400 MHz, CDCl_3_) 8.47 (1H, bs s, ArH), 7.85 (1H, s, ArH), 7.68 (2H, d, *J* = 7.2 Hz, ArH), 7.65 (1H, d, *J* = 8.3 Hz, ArH) 7.55 (1H, d, *J* = 8.2 Hz, ArH), 7.46 (2H, t, *J* = 7.6 Hz, ArH), 7.36 (1H, t, *J* = 7.4 Hz, ArH), 4.94 (1H, s, CH), 4.05 (3H, s, OCH_3_), 3.81 (6H, s, OCH_3_) *δ*_C_ (100 MHz, CDCl_3_) 168.0 (C_q_), 151.1 (C_q_), 141.5 (C_q_), 138.6 (C_q_), 135.9 (C_q_), 128.7, 128.2 (C_q_), 127.5, 127.1, 125.5, 122.8, 119.5, 113.9, 112.8 (C_q_), 53.9 (CH_3_), 53.0 (OCH_3_), 49.1 (CH).

*2-(6-Phenyl-1H-indol-3-yl)acetic acid* (**36**): Malonate **35** (30 mg, 78.7 µmol) was dissolved in methanol:water (9:1) and treated with potassium hydroxide (22.0 mg). The reaction refluxed for 16 h before additional potassium hydroxide (8.80 mg) was added. After one hour at reflux the reaction was cooled to room temperature and concentrated under reduced pressure. The resulting residue was dissolved in water and the aqueous solution was washed with ethyl acetate. The aqueous phase was then acidified with 1 M hydrochloric acid to pH 2 and the cloudy suspension was extracted with ethyl acetate (×3). The combined organic layers were dried over magnesium sulfate, then concentrated under reduced pressure to a light yellow solid. Purification by flash chromatography (10% methanol in dichloromethane) and subsequent recrystallisation from acetonitrile afforded the compound in question as a white solid (5.0 mg, 25%); mp 214–216 °C. *δ*_H_ (500 MHz, Acetone-*d*_6_) 7.69 (4H, m, ArH), 7.43 (2H, t, *J* = 7.8, ArH), 7.36 (1H, d, 8.3, ArH), 7.34 (1H, s, ArH), 7.30 (1H, t, *J* = 7.4 Hz, ArH), 3.78 (2H, s, CH_2_). *δ*_C_ (100 MHz, Acetone-*d*6) 172.4 (C_q_), 142.5 (C_q_), 134.7 (C_q_), 127.7, 127.0, 126.4, 124.6, 124.4 (C_q_), 119.1, 118.5, 109.6, 108.3 (C_q_), 30.6 (CH_2_). LRMS (ESI): *m*/*z* 252.1 [M + H]^+^. HRMS (ESI): *m*/*z* 250.0895 [M − H]^−^.

*Methyl 2-bromo-2-(6-bromobenzofuran-3-yl)acetate* (**37**): Compound **11** (500 mg, 1.86 mmol), *N*-bromosuccinimide (363.8 mg, 2.04 mmol) and azobisisobutyronitrile (61.0 mg, 0.370 mmol) were suspended in carbon tetrachloride (25 mL) and the mixture was refluxed for two hours. The reaction mixture was cooled to room temperature, filtered through a bed of Celite^®^, and the carbon tetrachloride was removed by distillation. The crude compound was purified by flash chromatography (0.5% ethyl acetate in hexanes) to afford the compound in question as an off-white solid (523 mg, 81%); mp 73–75 °C. δ_H_ (400 MHz, CDCl_3_) 7.88 (1H, s, ArH), 7.69 (1H, s, ArH), 7.66 (1H, d, *J* =8.4 Hz, ArH), 7.45 (1H, d, *J* = 8.4 Hz, ArH), 5.57 (1H, s, CH), 3.84 (3H, s, OCH_3_). δ_C_ (100 MHz, CDCl_3_) 168.0, 155.9, 144.5, 126.7, 124.6, 121.9, 118.7, 116.2, 115.3, 53.5, 36.5, 29.6. LRMS (ESI): *m*/*z* 368.52 [M (^79^Br, ^79^Br) + Na]^+^, 370.93 [M (^79^Br, ^81^Br) + Na]^+^, 372.92 [M (^81^Br, ^81^Br) + Na]^+^. HRMS (ESI): *m*/*z* 344.9786 [M (^79^Br, ^79^Br) − H]^−^.

*Methyl-2-azido-2-(6-bromobenzofuran-3-yl)acetate*, (**38**): Bromide **37** (760 mg, 2.18 mmol) was dissolved in dry *N*,*N*-dimethylformamide (20 mL) in an atmosphere of nitrogen. The stirring solution was chilled to −40 °C in a bath of acetonitrile/dry ice. Sodium azide (426 mg, 6.55 mmol) was added and the colourless solution gradually became yellow. The reaction was monitored by TLC, and after 50 min the solution was removed from the bath. Ethyl acetate and 1 M hydrochloric acid were added and the aqueous phase was extracted with ethyl acetate (×2). The combined organic phases were washed with brine (×5), dried over magnesium sulfate, then concentrated under reduced pressure to yield the product as a yellow oil (594 mg, 88%). δ_H_ (400 MHz, CDCl_3_) 7.72 (1H, s, ArH), 7.70 (1H, d, 1.6, ArH), 7.55 (1H, d, *J* = 8.4 Hz, ArH), 7.43 (1H, dd, *J* = 8.4 Hz, 1.6, ArH), 5.16 (1H, s, CH), 3.83 (3H, s, OCH_3_). δ_C_ (100 MHz, CDCl_3_) 168.7 (C_q_), 155.8 (C_q_), 144.2, 126.9 (C_q_), 124.4 (C_q_), 121.2, 118.8 (C_q_), 115.4, 114.4 (C_q_), 57.2 (CH), 53.2 (OCH_3_). LRMS (ESI): *m*/*z* not found.

*Methyl-2-amino-2-(6-bromobenzofuran-3-yl)acetate* (**39**): Azide **38** (114 mg, 0.369 mmol) was dissolved in dry methanol in an atmosphere of nitrogen. Adams catalyst (2.5% *w*/*w*) was added and the flask was purged three times with hydrogen. The reaction stirred at room temperature under hydrogen for 5 h, at which time TLC indicated negligible starting material. The reaction was filtered through Celite^®^, then concentrated to afford the amine as a yellow oil (97.1 mg, 0.342 mmol, 92%). δ_H_ (500 MHz, CDCl_3_) 7.66 (1H, d, *J* 1.5, ArH), 7.63 (1H, s, ArH), 7.56 (1H, d, *J* = 8.0 Hz, ArH), 7.38 (1H, dd, *J* = 8.3 Hz, 1.5, ArH), 4.84 (1H, s, CH), 3.74 (1H, s, CH_3_). δ_C_ (100 MHz, CDCl_3_) 173.4 (C_q_), 155.9 (C_q_), 143.0, 126.3, 125.0 (C_q_), 121.3, 119.8 (C_q_), 118.1 (C_q_), 115.2, 52.6 (OCH_3_), 50.5 (CH). LRMS (ESI): *m*/*z* 283.9 [M (^79^Br) + H]^+^, 285.9 [M (^81^Br) + H]^+^.

*Methyl 2-Acetamido-2-(6-bromobenzofuran-3-yl)acetate* (**40**): Amine **39** (100 mg, 0.326 mmol) was coupled to acetyl chloride (25.6 µL) according to General Procedure F to form the compound in question as an off-white solid (62.0 mg, 58%); mp 166–169 °C. δ_H_ (400 MHz, CDCl_3_) 7.65 (1H, s, ArH), 7.62 (1H, s, ArH), 7.49 (1H, d, *J* = 8.4 Hz, ArH), 7.37 (1H, d, *J* = 8.4 Hz, ArH), 6.58 (1H, d, *J* = 7.2 Hz, NH), 5.84 (1H, d, *J* = 7.5 Hz, CH), 3.76 (3H, s, OCH_3_), 2.02 (3H, s, CH_3_). δ_C_ (100 MHz, CDCl_3_) 170.6, 169.6, 155.7, 143.9, 126.6, 124.6, 120.9, 118.4, 116.5, 115.2, 53.0, 47.7, 22.9. LRMS (ESI): *m*/*z* 325.9 [M (^79^Br) + H]^+^, 327.9 [M (^81^Br) + H]^+^.

*Methyl 2-benzamido-2-(6-bromobenzofuran-3-yl) acetate* (**41**): Benzoic acid was coupled to amine **39** (100 mg, 0.626 mmol) according to general procedure G. The crude amide was purified by flash chromatography (dichloromethane) to give the compound in question as a white solid (69.3 mg, 55%); mp 161–162 °C. δ_H_ (500 MHz, CDCl_3_) 7.80–7.78 (2H, m, ArH), 7.69 (1H, s, ArH), 7.66 (1H, s, ArH), 7.56 (1H, d, *J* = 8.4 Hz, ArH), 7.51 (1H, t, *J* = 7.4 Hz, ArH), 7.43–7.39 (2H, m, ArH), 7.38 (1H, d, *J* = 8.4 Hz, ArH), 7.16 (1H, d, *J* = 7.1 Hz, NH), 6.04 (1H, d, *J* = 7.2 Hz, CH), 3.80 (3H, s, OCH_3_). δ_C_ (100 MHz, CDCl_3_) 170.7, 166.8, 155.8, 144.1, 133.2, 132.1, 128.6, 127.1, 126.6, 124.7, 120.9, 118.4, 116.5, 115.3, 53.1, 48.2. LRMS (ESI): *m*/*z* 388.0 [M (^79^Br) + H]^+^, 390.1 [M (^81^Br) + H]^+^.

*Methyl 2-(6-bromobenzofuran-3-yl)-2-(2-(4-methoxyphenyl)acetamido)acetate* (**42**): Amine **39** (46.3 mg, 0.163 mmol) was coupled to 4-methoxyphenylacetic acid (27.1 mg) according to General Procedure G to give the compound in question as a light yellow solid (37.0 mg, 52%); mp 114–116 °C. δ_H_ (400 MHz, CDCl_3_) 7.64 (1H, s, ArH), 7.53 (1H, s, ArH), 7.31 (2H, s, ArH), 7.15 (2H, d, *J* = 8.4 Hz, ArH), 6.87 (2H, d, *J* = 8.4 Hz, ArH), 6.39 (1H, br d, *J* = 7.2 Hz, NH), 5.80 (1H, d, *J* = 7.6 Hz, CH), 3.80 (3H, s, OCH_3_), 3.73 (1H, s, OCH_3_), 3.55 (2H, s, CH_2_). δ_C_ (100 MHz, CDCl_3_) 170.9 (C_q_), 170.4 (C_q_), 159.0 (C_q_), 155.7 (C_q_), 143.8, 130.4, 126.5, 126.0 (C_q_), 124.5 (C_q_), 120.9, 118.3 (C_q_), 116.3 (C_q_), 115.2, 114.5, 55.3 (OCH_3_), 53.0 (CH), 48.0 (OCH_3_), 42.5 (CH_2_). LRMS (ESI): *m*/*z* 432.0 [M (^79^Br) + H]^+^, 434.1 [M (^81^Br) + H]^+^.

*Methyl 2-(6-bromobenzofuran-3-yl)-2-(2-(2-fluorophenyl)acetamido)acetate* (**43**): Amine 39 (87.0 mg, 0.284 mmol) was coupled to 2-fluorophenylacetic acid (43.8 mg) according to General Procedure G to give the compound in question as a light yellow solid (105 mg, 71%); mp 154–156 °C. δ_H_ (400 MHz, CDCl_3_) 7.64 (1H, s, ArH), 7.57 (1H, s, ArH), 7.37 (1H, d, *J* = 8.4 Hz, Ar*H*), 7.33 (1H, d, *J* = 8.4 Hz, Ar*H*), 7.31–7.26 (2H, m, ArH), 7.14–7.05 (2H, m, ArH), 5.58 (1H, d, *J* = 7.2 Hz, NH), 5.81 (1H, d, *J* = 7.2 Hz, CH), 3.74 (3H, s, OCH_3_), 3.64 (2H, s, CH_2_). δ_C_ (100 MHz, CDCl_3_) 170.3, 169.4, 162.1, 159.6, 155.7, 143.9, 131.6, 131.5, 129.6, 129.5, 126.5, 124.6, 124.6, 124.5, 121.5, 121.3, 120.9, 118.4, 116.2, 115.7, 115.5, 115.2, 53.0, 48.1, 36.6. LRMS (ESI): *m*/*z* 420.2 [M (^79^Br) + H]^+^, 422.0 [M (^81^Br) + H]^+^.

*Methyl 2-(6-bromobenzofuran-3-yl)-2-(methylsulfonamido)acetate* (**44**): Amine **39** (150 mg, 0.528 mmol) was coupled to mesyl chloride (45 µL) according to General Procedure F. The crude yellow oil was purified by flash chromatography (dichloromethane) to afford the compound in question as a light-yellow oil (115 mg, 60%). δ_H_ (400 MHz, CDCl_3_) 7.69 (1H, s, ArH), 7.68 (1H, s, ArH), 7.52 (1H, d, *J* = 8.4 Hz, ArH), 7.39 (1H, d, *J* = 8.4 Hz, ArH), 5.74 (1H, d, *J* = 6.7 Hz, NH), 5.46 (1H, d, *J* = 6.7 Hz, CH), 3.77 (3H, s, OCH_3_), 2.84 (3H, s, CH_3_). δ_C_ (100 MHz, CDCl_3_) 170.0 (C_q_), 155.8 (C_q_), 144.2, 126.9, 124.0 (C_q_), 121.0, 118.7 (C_q_), 116.0 (C_q_), 115.4, 53.4 (CH), 51.4 (OCH_3_), 42.1 (CH_3_). LRMS (ESI): *m*/*z* 361.9 [M (^79^Br) + H]^+^, 363.9 [M (^81^Br) + H]^+^.

*Methyl 2-(6-bromobenzofuran-3-yl)-2-(phenylsulfonamido)acetate* (**45**): Amine **39** (75 mg, 245 µmol) was coupled to benzenesulfonyl chloride according to General Procedure F. The crude yellow solid was purified by flash chromatography (15% ethyl acetate in hexanes) to afford the compound in question as a pale yellow solid (16.0 mg, 15%); mp 133–135 °C. δ_H_ (400 MHz, CDCl_3_) 7.74–7.72 (2H, m, ArH), 7.57 (1H, s, ArH), 4.48–7.45 (2H, m, ArH), 7.38–7.33 (3H, m, ArH), 7.30 (1H, d, *J* = 8.4 Hz, ArH), 5.84 (1H, d, *J* = 7.6 Hz, NH), 5.30 (1H, d, *J* = 7.6 Hz, CH), 3.59 (3H, s, OCH_3_). δ_C_ (100 MHz, CDCl_3_) 169.5, 155.64, 144.0, 139.5, 132.8, 128.8, 127.0, 126.6, 124.1, 121.0, 118.4, 115.5, 115.1, 53.2, 51.4. LRMS (ESI): *m*/*z* 446.4 [M (^79^Br) + H]^+^, 448.2 [M (^81^Br) + H]^+^.

*2-Acetamido-2-(6-bromobenzofuran-3-yl)acetic acid* (**46**): Ester **40** (61.0 mg, 0.187 mmol) was hydrolysed using 2 M methanolic sodium hydroxide (5 equiv, 468 µL) according to General Procedure A to give the compound in question as a light yellow solid (57.0 mg, 98%); mp 194–196 °C. δ_H_ (500 MHz, Acetone-*d*_6_) 7.96 (1H, s, ArH), 7.78 (1H, s, ArH), 7.70 (1H, d, *J* = 8.4 Hz, ArH), 7.45 (1H, d, *J* = 8.4 Hz, ArH), 5.83–5.81 (1H, m, CH), 1.99 (3H, s, CH_3_). δ_C_ (100 MHz, Acetone-*d*_6_) 171.4 (C_q_), 170.5 (C_q_), 156.7 (C_q_), 145.6, 127.1, 126.4 (C_q_), 123.0, 118.6 (C_q_), 118.3 (C_q_), 115.7, 48.6 (CH), 22.6 (CH_3_). LRMS (ESI): *m*/*z* 309.94 [M (^79^Br) + H]^+^, 311.82 [M (^81^Br) + H]^+^.

*2-Benzamido-2-(6-bromobenzofuran-3-yl)-2-acetic acid* (**47**): Ester **41** (69.3 mg, 0.179 mmol) was hydrolysed using 2 M methanolic sodium hydroxide (5 equiv, 448 µL) according to General Procedure A to give the compound in question as a white solid (64.2 mg, 96%); mp 209–212 °C. δ_H_ (500 MHz, DMSO-*d*_6_) 9.18 (1H, d, *J* = 7.1 Hz, NH), 8.13 (1H, s, ArH), 7.93–7.89 (3H, m, ArH), 7.70 (1H, d, *J* = 8.4 Hz, ArH), 7.54 (1H, t, *J* = 8.4 Hz, ArH), 7.50–7.44 (3H, m, ArH), 5.83 (1H, d, *J* = 7.2 Hz, CH). δ_C_ (100 MHz, DMSO-*d*_6_) 171.0 (C_q_), 166.5 (C_q_), 155.0 (C_q_), 145.1, 133.59 (C_q_), 131.6, 128.3, 127.7, 126.1 (C_q_), 125.8, 122.3, 117.2 (C_q_), 116.8 (C_q_), 114.7, 48.2 (CH). LRMS (ESI): *m*/*z* 747.5 [2 M]^+^. HRMS (ESI): *m*/*z* 371.9920 [M (^79^Br) − H]^−^, 373,9904 [M (^81^Br) − H]^−^.

*2-(6-Bromobenzofuran-3-yl)-2-(2-(4-methoxyphenyl)acetamido)acetic acid* (**48**): Ester **42** (15 mg, 34.7 µmol) was hydrolysed using 2 M methanolic sodium hydroxide (5 equiv, 87.0 µL) according to General Procedure A to afford the compound in question as an off-white solid (8.4 mg, 58%); mp 159–161 °C. δ_H_ (400 MHz, CD_3_OD) 7.80 (1H, s, ArH), 7.71 (1H, s, ArH), 7.48 (1H, d, *J* = 8.4 Hz, ArH), 7.35 (1H, d, *J* = 8.4 Hz, ArH), 7.19 (2H, d, 8.6, ArH), 6.84 (2H, d, *J* = 8.6 Hz, ArH), 5.70 (1H, s, CH), 3.77 (3H, s, OCH_3_), 3.52 (2H, s, CH_2_). LRMS (ESI): *m*/*z* 415.89 [M (^79^Br) + H]^+^, 417.85 [M (^81^Br) + H]^+^. HRMS (ESI): *m*/*z* 416.0196 [M (^79^Br) − H]^−^, 418.0181 [M (^81^Br) − H]^−^.

*2-(6-Bromobenzofuran-3-yl)-2-(2-(2-fluorophenyl)acetamido)acetic acid* (**49**): Ester **43** (43.5 mg, 0.104 mmol) was hydrolysed using 2 M methanolic sodium hydroxide (5 equiv, 259 µL) according to General Procedure A to afford the compound in question as a light yellow solid (20.0 mg, 50%); mp 209–211 °C. δ_H_ (500 MHz, DMSO-*d*_6_) 13.09 (1H, br s, COOH), 8.97 (1H, d, *J* = 7.0 Hz, NH), 8.07 (1H, s, ArH), 7.93 (1H, s, ArH), 7.64 (1H, d, *J* = 8.5 Hz, ArH), 7.48 (1H, d, *J* = 8.5 Hz, ArH), 7.34–7.25 (2H, m, ArH), 7.15–7.10 (2H, m, ArH), 5.59 (1H, d, *J* = 7.0 Hz, CH), 3.60 (2H, s, CH_2_). δ_C_ (125 MHz, DMSO-*d*_6_) 171.4, 169.7, 161.1 (d, *J* = 243 Hz), 155.6, 145.3, 132.2 (d, *J* = 3.75 Hz), 129.13, 129.07, 126.6, 125.8, 124.6 (d, *J* = 3.73 Hz), 122.7, 117.8, 117.4, 115.4 (d, *J* = 21 Hz), 115.2, 48.3, 35.1. LRMS (ESI): *m*/*z* 404.4 [M − H]^−^. HRMS (ESI): *m*/*z* 403.9985 [M (^79^Br) − H]^−^, 405.997 [M (^81^Br) − H]^−^.

*2-(6-Bromobenzofuran-3-yl)-2-(methylsulfonamido)acetic acid* (**50**): Ester **44** (115 mg, 318 µmol) was hydrolysed using 2 M methanolic sodium hydroxide (5 equiv, 794 µL) according to General Method A to afford the compound in question as a light yellow solid (75 mg, 68%); mp 140–142 °C. δ_H_ (400 MHz, DMSO-*d*_6_) 8.16 (1H, d, *J* = 8.3 Hz, ArH), 8.06 (1H, s, ArH), 7.91 (1H, s, ArH), 7.73 (1H, d, *J* = 8.4 Hz, ArH), 7.48 (1H, d, *J* = 8.4 Hz, ArH), 5.34 (1H, d, *J* = 8.3 Hz, CH), 2.92 (2H, s, CH_2_). δ_C_ (100 MHz, DMSO-*d*_6_) 171.3 (C_q_), 155.6 (C_q_), 145.3, 126.5, 125.5 (C_q_), 122.8, 117.8 (C_q_), 117.7 (C_q_), 155.2, 51.4 (CH), 41.6 (CH_3_). LRMS (ESI): *m*/*z* 345.95 [M (^79^Br) + H]^+^, 347.84 [M (^81^Br) + H]^+^. HRMS (ESI): *m*/*z* 345.9425 [M (^79^Br) − H]^−^, 347.9403 [M (^81^Br) − H]^−^.

*2-(6-Bromobenzofuran-3-yl)-2-(phenylsulfonamido)acetic acid* (**51**): Ester **45** (15.0 mg, 35.4 µmol) was hydrolysed using 2 M methanolic sodium hydroxide (5 equiv, 88.5 µL) according to General Method A to afford the compound in question as an off-white solid (13.3 mg, 92%); mp 179–182 °C. δ_H_ (500 MHz, Acetone-*d*_6_) 7.83 (1H, s, ArH), 7.82–7.80 (2H, m, ArH), 7.69 (1H, s, ArH), 7.62 (1H, d, *J* = 8.4 Hz, ArH), 7.52 (1H, t, *J* = 7.4 Hz, ArH), 7.43 (2H, t, *J* = 7.7 Hz, ArH), 7.38 (1H, d, *J* = 8.4 Hz, ArH), 5.37 (1H, s, CH). δ_C_ (125 MHz, Acetone-*d*_6_) 170.5, 156.6, 145.8, 142.0, 133.3, 129.7, 127.8, 127.0, 125.8, 123.0, 118.6, 117.8, 115.6, 52.2. HRMS (ESI): *m*/*z* 407.9707 [M (^79^Br) − H]^−^, 409.9614 [M (^81^Br) − H]^−^.

### 4.3. Expression and Purification of Unlabelled and ^15^N-Labelled EcDsbA

Unlabelled and ^15^N-labelled *Ec*DsbA were prepared as previously described [17]. Briefly, both unlabelled and ^15^N-labelled *Ec*DsbA were expressed by autoinduction using appropriate media. After harvesting the cells, periplasmic proteins were extracted by resuspending cell pellet with lysis buffer (20 mM Tris-HCl, pH 8.0, 25 mM NaCl, 4 mg/mL colistin sulfate) and incubating for 18–24 h with gentle stirring at 4 °C. After centrifugation, *Ec*DsbA was purified from the supernatant using a three-step purification method, including hydrophobic interaction chromatography, anion exchange chromatography, and size exclusion chromatography. DsbA was oxidised with copper-phenanthroline and buffer exchanged to the final storage buffer (20 mM HEPES, pH 7.0).

### 4.4. Protein Crystallisation and Structure Determination

*Ec*DsbA was crystallised as previously described [17]. Briefly, 1 µL of 30 mg/mL *Ec*DsbA was mixed with an equal volume of crystallisation buffer (11–13% PEG8000, 5–7.5% glycerol, 1 mM CuCl_2_, 100 mM, sodium cacodylate pH 6.1) and equilibrated against 500 µL of reservoir buffer at 20 °C using hanging drop vapour diffusion. For compound soaking, crystals were transferred into 2 µL drops of 24% PEG8000, 22% glycerol, and 100 mM sodium cacodylate (pH 6.1) containing a compound of interest at the final concentration of 10 mM (5% DMSO) and were incubated for 3–6 h. Crystals were mounted on loops and flash-cooled in liquid nitrogen.

Datasets were collected at the Australian synchrotron on MX1 and MX2 beamlines using the Blue-Ice software (Irvine, CA, USA) [31]. MX1 beamline was equipped with an ADSC Quantum 210r detector and MX2 with an ADSC Quantum 315r detector. All datasets were indexed and integrated with iMOSFLM [32] or XDS [33]—and scaled using AIMLESS [34,35] or SCALA [36]. Phasing was performed by molecular replacement with Phaser [37] using the previously solved structure of *Ec*DsbA as a search model (PDB code 1FVK) [38]. The final structure was obtained after several rounds of manual model building using Coot [39] and refinement in phenix.refine [40]. Data collection and refinement statistics are summarised in Table S1. Structures, factors and coordinates have been deposited in the Protein Data Bank (PDB; http://www.pdb.org) under the accession codes of 6PMF, 6POQ, 6POH, 6PML, 6PVY, 6POI, and 6PVZ.

### 4.5. NMR Spectroscopy

For ligand-detected NMR spectroscopy, two samples (compound alone and compound + protein) were prepared for each compound. Unlabelled *Ec*DsbA was prepared at 10 µM in a buffer of 50 mM sodium phosphate, pH 6.8, 25 mM NaCl, 100 µM 4,4-dimethyl 4-silapentane-1-sulfonic acid (DSS), and 10% ^2^H_2_O. Compound was added to the protein sample to achieve final concentration of 500 µM (2% ^2^H_6_-DMSO). ^1^H 1D spectrum was acquired for each compound at 500 µM as a reference spectrum. All ligand-detected NMR experiments (STD, CPMG, and waterLOGSY) were acquired at 298 K on a Bruker Avance III 600 MHz NMR spectrometer equipped with a 5 mm TXI CryoProbe (Bruker, Billerica, MA, USA). STD spectra were acquired with 3 s of saturation at 1 ppm (on-resonance) and 33.3 ppm (off-resonance). WaterLOGSY spectra were acquired for compound alone and compound + protein with a 0.52 s acquisition time, 3 s relaxation delay, and 3 s Nuclear Overhauser effect (NOE) mixing time. CPMG spectra for compound alone and compound + protein were acquired with a constant spin echo delay of 1 ms and spin-lock period of 350 ms. The data was analysed using Topspin3.5 (Bruker) and DSS was used to reference the spectra.

For the 2D ^1^H-^15^N-HSQC titration experiment, 100 µM of ^15^N-labelled *Ec*DsbA in a buffer of 50 mM HEPES, pH 6.8, 50 mM NaCl, 2% ^2^H_6_-DMSO, and 10% ^2^H_2_O, was titrated with increasing concentrations of compounds (0, 0.625, 0.125, 0.25, 0.5, and 1 mM). Data were acquired on a Bruker 600 MHz spectrometer equipped with a 5 mm TXI CryoProbe and a Bruker 700 MHz spectrometer equipped with a 5 mm TXI CryoProbe. Data was processed by Topspin3.5 (Bruker) and analysed by Sparky (T. D. Goddard and D. G. Kneller, SPARKY 3, University of California, San Francisco (UCSF)). Weighted chemical shift perturbations (CSP) that were observed upon addition of compounds to *Ec*DsbA were calculated using an Equation (1) in [41]:

(1) $$\mathrm{CSP}=\sqrt{\Delta \delta_{H}{}^{2}+{\left(0.2\times \Delta \delta_{N}\right)}^{2}}$$

where Δ*δ_H_* and Δ*δ_N_* denote the change in chemical shift of amide proton and nitrogen resonances upon addition of the compound. Equilibrium dissociation constants (*K*_D_) were determined by fitting the plot of CSP against compound concentrations using one site with ligand depletion model (Equation (2)):

(2) $$\mathrm{CSP}=\frac{CSP_{max}\times \left[\left(K_{D}+P_{t}+L_{t}\right)-\sqrt{\left({\left(K_{D}+P_{t}+L_{t}\right)}^{2}-4\times P_{t}\times L_{t}\right)}\right]}{2\times P_{t}}$$

where CSP is the measured CSP at a given concentration, *CSP_max_* is the CSP observed at the saturating concentration of compound, and *P_t_* and *L_t_* are the total protein and compound concentrations. The ligand efficiency (LE) is calculated using the Equation (3) in [42]:

(3) $$\mathrm{LE}=-\frac{\Delta G}{HAC}=-\frac{RTln\left(K_{D}\right)}{HAC}$$

where *R* is gas constant (1.9858775 cal K^−1^ mol^−1^), *T* is temperature (298 K), *K*_D_ is equilibrium dissociation constant, and HAC is the number of heavy (non-hydrogen) atoms.

## Figures and Tables

**Table 1:**
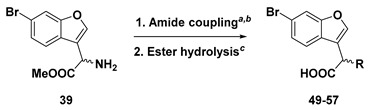
Amide/sulfonamide formation and subsequent ester hydrolysis.

Entry	R	Amide Coupling Product (Yield)	Hydrolysis ^c^ (Yield)
1	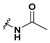	**40**^a^ (58%)	**46** (98%)
2	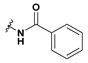	**41**^b^ (55%)	**47** (96%)
3	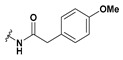	**42**^b^ (52%)	**48** (58%)
4	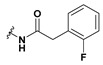	**43**^b^ (88%)	**49** (50%)
5	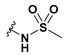	**44**^a^ (60%)	**50** (68%)
6	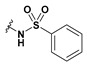	**45**^a^ (15%)	**51** (92%)

^a^ Reagents and conditions: amine, acid/sulfonyl chloride, Et_3_N, Et_2_O. ^b^ Reagents and conditions: (1) acid, PCl_5_, DCM, reflux, 30 min; (2) amine, Et_3_N, DCM, 2–5 h. ^c^ Reagents and conditions: 2 M methanolic NaOH, DCM:MeOH (9:1).

**Table 2:** Summary of NMR binding data.

ID	Structure	Crystal Structure in *Ec*DsbA	wLOGSY	CPMG	STD	Dose Response HSQC *K*_D_ (µM)	Ligand Efficiency (kcal mol^−1^ HAC^−1^)
**2**	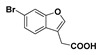	No	Pass	Fail	Pass	-	-
**15**	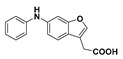	Yes	Pass	Pass	Pass	-	-
**16**	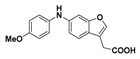	Yes	Pass	Pass	Fail	2700 ± 920	0.16
**21**	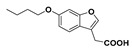	Yes	Pass	Pass	Pass	1970 ± 640	0.20
**22**	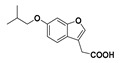	Yes	Fail	Pass	Fail	-	-
**23**	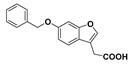	Yes	Pass	Pass	Pass	820 ± 140	0.20
**25**	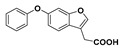	Yes	Pass	Pass	Pass	326 ± 25	0.24
**26**	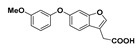	No	Pass	Pass	Pass	639 ± 87	0.20
**28**	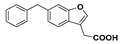	Yes	Pass	Pass	Pass	341 ± 47	0.24
**31**	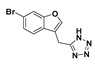	No	Pass	Pass	Pass	-	-
**36**	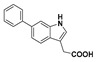	No	Pass	Pass	Pass	-	-
**46**	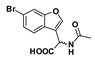	No	Pass	Fail	Fail	-	-
**47**	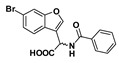	No	Pass	Fail	Fail	-	-
**48**	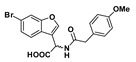	No	Pass	Fail	Fail	1350 ± 420	0.15
**49**	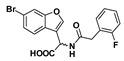	No	Pass	Fail	Fail	>>1000	-
**50**	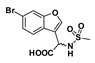	Yes	Pass	Pass	Pass	-	-

## Supplementary Materials

The following are available online: Figure S1: Electron density maps for compounds. Figure S2: Co-crystal structure of **50**. Table S1: Data collection and refinement statistics. NMR Figures: 1H and 13C-NMR characterisation of all compounds presented in the main text.

## References

[1] Heras B., Shouldice S.R., Totsika M., Scanlon M.J., Schembri M.A., Martin J.L. (2009). DSB proteins and bacterial pathogenicity. Nat. Rev. Genet..

[2] Totsika M., Heras B., Wurpel D.J., Schembri M.A. (2009). Characterization of two homologous disulfide bond systems involved in virulence factor biogenesis in uropathogenic Escherichia coli CFT073. J. Bacteriol..

[3] Bardwell J.C., McGovern K., Beckwith J. (1991). Identification of a protein required for disulfide bond formation in vivo. Cell.

[4] Yu J. (1998). Inactivation of DsbA, but not DsbC and DsbD, affects the intracellular survival and virulence of Shigella flexneri. Infect. Immun..

[5] Burall L.S., Harro J.M., Li X., Lockatell C., Himpsl S.D., Hebel J.R., Johnson D.E., Mobley H.L.T. (2004). Proteus mirabilis genes that contribute to pathogenesis of urinary tract infection: Identification of 25 signature-tagged mutants attenuated at least 100-fold. Infect. Immun..

[6] Ireland P.M., McMahon R.M., Marshall L.E., Halili M., Furlong E., Tay S., Martin J.L., Sarkar-Tyson M. (2014). Disarming Burkholderia pseudomallei: Structural and functional characterization of a disulfide oxidoreductase (DsbA) required for virulence in vivo. Antioxidants Redox Signal..

[7] Heras B., Scanlon M.J., Martin J.L. (2015). Targeting virulence not viability in the search for future antibacterials. Br. J. Clin. Pharmacol..

[8] Akiyama Y., Kamitani S., Kusukawa N., Ito K. (1992). In vitro catalysis of oxidative folding of disulfide-bonded proteins by the Escherichia coli DsbA (ppfA) gene product. J. Boil. Chem..

[9] Martin J.L. (1995). Thioredoxin—A fold for all reasons. Structure.

[10] Martin J.L., Bardwell J.C.A., Kuriyan J. (1993). Crystal structure of the DsbA protein required for disulphide bond formation in vivo. Nature.

[11] Shouldice S.R., Heras B., Walden P.M., Totsika M., Schembri M.A., Martin J.L. (2011). Structure and function of DsbA, a key bacterial oxidative folding catalyst. Antioxidants Redox Signal..

[12] Kadokura H., Tian H., Zander T., Bardwell J.C.A., Beckwith J. (2004). Snapshots of DsbA in action: Detection of proteins in the process of oxidative folding. Science.

[13] Duprez W., Premkumar L., Halili M.A., Lindahl F., Reid R.C., Fairlie D.P., Martin J.L. (2015). Peptide inhibitors of the Escherichia coli DsbA oxidative machinery essential for bacterial virulence. J. Med. Chem..

[14] Landeta C., Blazyk J.L., Hatahet F., Meehan B.M., Eser M., Myrick A., Bronstain L., Minami S., Arnold H., Ke N. (2015). Compounds targeting disulfide bond forming enzyme DsbB of Gram-negative bacteria. Nat. Methods.

[15] Halili M.A., Bachu P., Lindahl F., Bechara C., Mohanty B., Reid R.C., Scanlon M.J., Robinson C.V., Fairlie D.P., Martin J.L. (2015). Small molecule inhibitors of disulfide bond formation by the bacterial DsbA–DsbB dual enzyme system. ACS Chem. Boil..

[16] Früh V., Zhou Y., Chen D., Loch C., Ab E., Grinkova Y.N., Verheij H., Sligar S.G., Bushweller J.H., Siegal G. (2010). Application of fragment-based drug discovery to membrane proteins: identification of ligands of the integral membrane enzyme DsbB. Chem. Boil..

[17] Adams L.A., Sharma P., Mohanty B., Ilyichova O.V., Mulcair M.D., Williams M.L., Gleeson E.C., Totsika M., Doak B.C., Caria S. (2015). Application of fragment-based screening to the design of inhibitors of Escherichia coli DsbA. Angew. Chem. Int. Ed..

[18] Coutant E.P., Janin Y.L. (2015). A study of Negishi cross-coupling reactions with benzylzinc halides to prepare original 3-ethoxypyrazoles. Synthesis.

[19] Suárez-Castillo O.R., Meléndez-Rodríguez M., Beiza-Granados L., Cano-Escudero I.C., Morales-Ríos M.S., Joseph-Nathan P. (2011). C-6 regioselective bromination of methyl indolyl-3-acetate. Nat. Prod. Commun..

[20] Mayer M., Meyer B. (1999). Characterization of ligand binding by saturation transfer difference NMR spectroscopy. Angew. Chem. Int. Ed..

[21] Carr H.Y., Purcell E.M. (1954). Effects of diffusion on free precession in nuclear magnetic resonance experiments. Phys. Rev..

[22] Dalvit C., Fogliatto G., Stewart A., Veronesi M., Stockman B. (2001). WaterLOGSY as a method for primary NMR screening: practical aspects and range of applicability. J. Biomol. NMR.

[23] Hubbard R.E., Murray J.B., Kuo L.C. (2011). Chapter twenty—Experiences in fragment-based lead discovery. Methods in Enzymology.

[24] Armarego W.L.F., Chai C.L.L., Armarego W.L.F., Chai C.L.L. (2003). Chapter 4—Purification of organic chemicals. Purification of Laboratory Chemicals.

[25] Theodorou V., Skobridis K., Tzakos A.G., Ragoussis V. (2007). A simple method for the alkaline hydrolysis of esters. Tetrahedron Lett..

[26] Akita S., Umezawa N., Higuchi T. (2005). On-bead fluorescence assay for serine/threonine kinases. Org. Lett..

[27] Frasinyuk M.S., Bondarenko S.P., Khilya V.P., Frasinyuk M., Bondarenko S. (2009). Synthesis and properties of 4-chloromethyl-6-hydroxy-coumarins and 4-(2-benzofuryl)-6-hydroxycoumarins. Chem. Heterocycl. Compd..

[28] Basanagouda M., Narayanachar, Majati I.B., Mulimani S.S., Sunnal S.B., Nadiger R.V., Ghanti A.S., Gudageri S.F., Naik R., Nayak A. (2015). Efficient and convenient method for synthesis of benzofuran-3-acetic acids and naphthafuran-acetic acids. Synth. Commun..

[29] Wood P.M., Woo L.W.L., Labrosse J.-R., Thomas M.P., Mahon M.F., Chander S.K., Purohit A., Reed M.J., Potter B.V.L. (2010). Bicyclic derivatives of the potent dual aromatase-steroid sulfatase inhibitor 2-bromo-4-{[(4-cyanophenyl)(4*H*-1,2,4-triazol-4-yl)amino]methyl}phenylsulfamate: Synthesis, SAR, crystal structure, and in vitro and in vivo activities. Chem. Med. Chem..

[30] Rozhkov V.S., Smushkevich Y.I., Suvorov N.N. (1980). Indole derivatives. 119. C-acylation of methyl (indol-3-yl)acetate. synthesis of 5-(indol-3-yl)pyrimidines. Khim. Geterotsikl. Soedin..

[31] McPhillips T.M., McPhillips S.E., Chiu H.-J., Cohen A.E., Deacon A.M., Ellis P.J., Garman E., Gonzalez A., Sauter N.K., Phizackerley R.P. (2002). Blu-ice and the distributed control system: Software for data acquisition and instrument control at macromolecular crystallography beamlines. J. Synchrotron Radiat..

[32] Battye T.G.G., Kontogiannis L., Johnson O., Powell H.R., Leslie A.G.W. (2011). iMOSFLM: A new graphical interface for diffraction-image processing with MOSFLM. Acta Crystallogr. Sect. D Boil. Crystallogr..

[33] Kabsch W. (2010). Xds. Acta Crystallogr. Sect. D Biol. Crystallogr..

[34] Evans P.R., Murshudov G.N. (2013). How good are my data and what is the resolution?. Acta Crystallogr. Sect. D Biol. Crystallogr..

[35] Collaborative C.P. (1994). The CCP4 suite: Programs for protein crystallography. Acta Crystallogr. Sect. D Biol. Crystallogr..

[36] Evans P. (2006). Scaling and assessment of data quality. Acta Crystallogr. Sect. D Biol. Crystallogr..

[37] McCoy A.J., Grosse-Kunstleve R.W., Adams P.D., Winn M.D., Storoni L.C., Read R.J. (2007). Phaser crystallographic software. J. Appl. Crystallogr..

[38] Guddat L.W., Bardwell J.C.A., Glockshuber R., Huber-Wunderlich M., Zander T., Martin J.L. (1997). Structural analysis of three His32 mutants of DsbA: Support for an electrostatic role of His32 in DsbA stability. Protein Sci..

[39] Emsley P., Cowtan K.D. (2004). Coot: Model-building tools for molecular graphics. Acta Crystallogr. Sect. D Boil. Crystallogr..

[40] Adams P.D., Afonine P.V., Bunkóczi G., Chen V.B., Davis I.W., Echols N., Headd J.J., Hung L.-W., Kapral G.J., Grosse-Kunstleve R.W. (2010). PHENIX: A comprehensive Python-based system for macromolecular structure solution. Acta Crystallogr. Sect. D Boil. Crystallogr..

[41] Ziarek J.J., Peterson F.C., Lytle B.L., Volkman B.F., Kuo L.C. (2011). Chapter ten—Binding site identification and structure determination of protein–ligand complexes by NMR: A semiautomated approach. Methods in Enzymology.

[42] Mortenson P.N., Murray C.W. (2011). Assessing the lipophilicity of fragments and early hits. J. Comput. Mol. Des..

