# Supplementary material for: The Fragment-Based Development of a Benzofuran Hit as a New Class of Escherichia coli DsbA Inhibitors
Source: Molecules. 2019 Oct 18;24(20):3756. doi: 10.3390/molecules24203756 (PMC6832960; doi:10.3390/molecules24203756)

Fragment-based development of a benzofuran hit as a new class of *Escherichia coli* DsbA inhibitors

Luke F. Duncan[1†], **Geqing Wang** [2†], Olga V. Ilyichova [3], Martin J. Scanlon[3,4], Begoña Heras[2] and Belinda M. Abbott[1*]

[1]Department of Chemistry and Physics, La Trobe Institute for Molecular Science, La Trobe University, Melbourne, VIC 3086, Australia

[2]Department of Biochemistry and Genetics, La Trobe Institute for Molecular Science, La Trobe University, Melbourne, VIC 3086, Australia

[3]Medicinal Chemistry, Monash Institute of Pharmaceutical Sciences, Monash University, 381 Royal Parade, Parkville, VIC 3052, Australia

[4]ARC Training Centre for Fragment Based Design, Monash Institute of Pharmaceutical Sciences, Monash University, 381 Royal Parade, Parkville, VIC 3052, Australia

† Both authors contributed equally to this work

* Corresponding author

Table of contents

Figure S1 Electron density maps for compounds ……………………………………………………………S3

Figure S2. Co-crystal structure of **50**……………………………………………………………………………S2

Table S1. Data collection and refinement statistics……………………………………………………S4-5

^{1}H NMR and ^{13}C NMR spectra for novel compounds S6-66

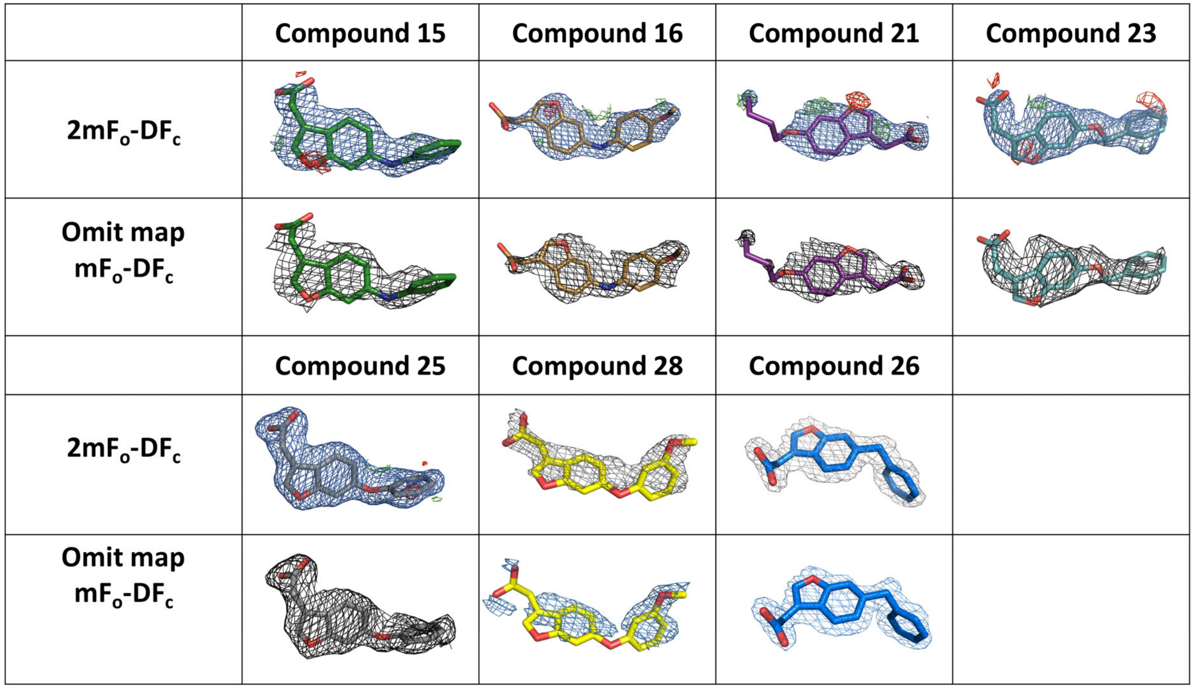

Figure S1. σ_A-weighted 2mFo−DFc electron density maps for compounds are contoured at 1σ and simulated annealing omit σA-weighted mFo-DFc electron density maps for compounds are contoured at 2.5σ.

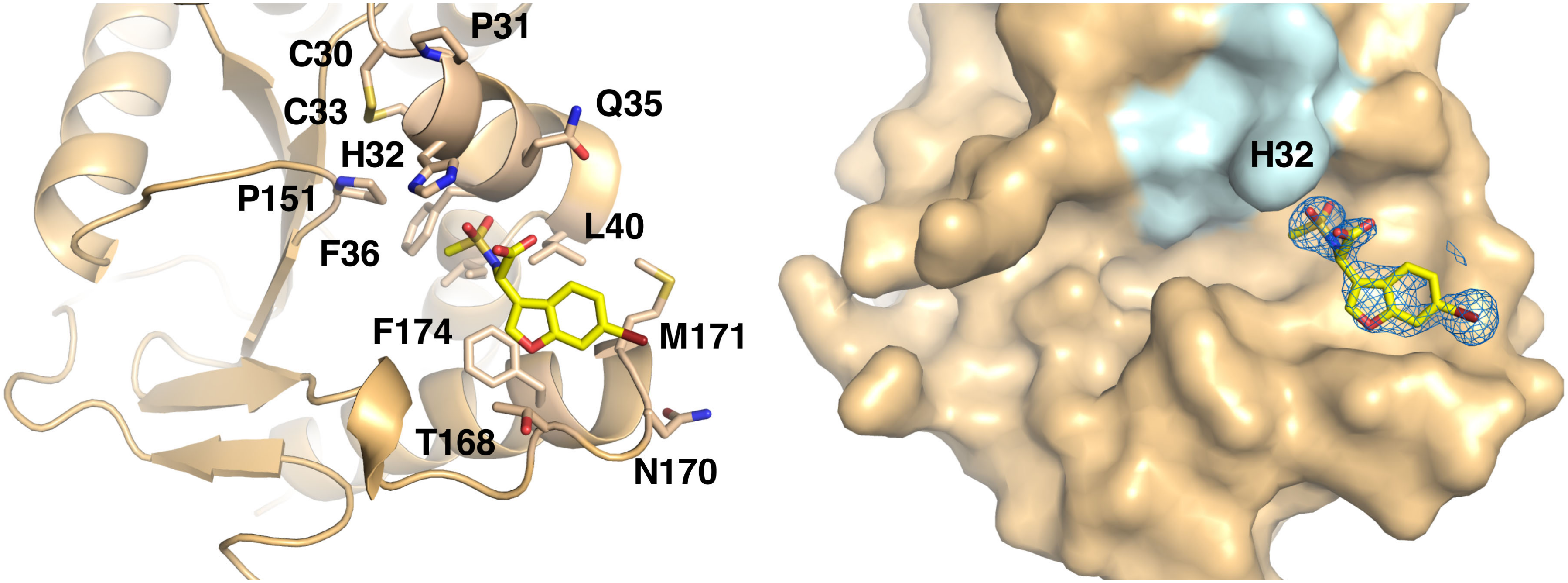

Figure S2. Co-crystal structure of 50 ((S)-enantiomer). Left panel: *Ec*DsbA (light yellow) is presented as a cartoon. The residues within 4 Å of **50** (yellow) are shown as sticks and labelled individually. The active site residues C30-P31-H32-C33 are also shown as sticks. Right panel: Surface presentation of *Ec*DsbA in the same orientation. The active site residues (C30, P31, H32, C33, V150 and P151) are shown in cyan colour. $2mF_o$-DF_c electron density map for **50** is contoured at 1σ and shown as blue mesh.

Table S1. Data collection and refinement statistics

	Compound 15	Compound 16	Compound 21	Compound 23	Compound 26	Compound 25	Compound 28
PDB ID	6PMF	6POQ	6POH	6PML	6PVY	6POI	6PVZ
Wavelength	0.9537	0.9537	0.9537	0.9537	0.95366	0.9537	0.95370
Resolution range (Å)	37.28 - 1.95 (2.02 - 1.95)	34.97 - 1.8 (1.864 - 1.8)	31.87 - 1.67 (1.73 - 1.67)	34.84 - 2.0 (2.072 - 2.0)	34.63-1.736 (1.799-1.736)	33.37 - 1.77 (1.833 - 1.77)	28.64-1.986 (2.057-1.986)
Space group	C 1 2 1	C 1 2 1	C 1 2 1	C 1 2 1	C 1 2 1	C 1 2 1	C 1 2 1
Cell dimensions (Å) (a, b, c)	117.72 64.07 74.73	118.16 63.71 74.79	118.05 63.74 74.54	117.54 63.74 96.19	117.32, 63.90, 74.46	117.75 63.67 96.60	117.59, 63.43, 74.23
Angles (°)(α, β, γ)	90 125.53 90	90 125.25 90	90 124.95 90	58149 (5330)	90.0, 125.8, 90.0	90 140.96 90	90.0, 125.0, 90.0
Total reflections	65749 (6503)	178012 (17713)	105145 (10495)	58149 (5330)	161666 (14562)	87194 (8620)	60764 (5911)
Unique reflections	33084 (3296)	42174 (4225)	52679 (5263)	29758 (2848)	45579 (4393)	43840 (4330)	30859 (3051)
Multiplicity	2.0 (2.0)	4.2 (4.2)	2.0 (2.0)	2.0 (1.9)	3.5 (3.3)	2.0 (2.0)	2.0 (1.9)
Completeness (%)	99.73 (99.52)	99.98 (100.00)	99.92 (99.96)	97.62 (94.12)	98.66 (95.54)	99.72 (99.79)	99.39 (98.67)
$<I/\sigma_I>$	12.80 (2.06)	21.67 (2.24)	14.27 (2.25)	9.27 (1.72)	9.69 (1.18)	14.08 (2.17)	16.13 (4.34)
Wilson B-factor	29.54	29.18	19.21	25.68	33.26	20.85	20.76
R_{merge}	0.03437 (0.3369)	0.08923 (0.6813)	0.03339 (0.3625)	0.05591 (0.4229)	0.0632 (0.5206)	0.03635 (0.3825)	0.0281 (0.1683)
R_{meas}	0.04861 (0.4764)	0.1023 (0.7823)	0.04723 (0.5127)	0.07907 (0.598)	0.07375 (0.6214)	0.05141 (0.5409)	0.03974 (0.238)
R_{pim}	0.03437 (0.3369)	0.04974 (0.381)	0.03339 (0.3625)	0.05591 (0.4229)	0.03747 (0.3344)	0.03635 (0.3825)	0.0281 (0.1683)
$CC_{1/2}$	0.998 (0.88)	0.994 (0.79)	0.999 (0.742)	0.996 (0.662)	0.995 (0.825)	0.999 (0.725)	0.999 (0.92)
CC*	1 (0.968)	0.999 (0.94)	1 (0.923)	0.999 (0.893)	0.999 (0.951)	1 (0.917)	1 (0.979)
Reflections used in refinement	33049 (3285)	42173 (4225)	52674 (5263)	29757 (2849)	45559 (4391)	43839 (4330)	30858 (3051)
Reflections used for R_{free}	1652 (177)	2187 (198)	2588 (240)	1618 (142)	2418 (209)	2198 (209)	1570 (190)
R_{work}	0.1909 (0.3027)	0.1638 (0.2665)	0.1652 (0.2625)	0.1824 (0.2583)	0.1925 (0.2954)	0.1695 (0.2474)	0.1663 (0.1972)
R_{free}	0.2258 (0.3505)	0.2011 (0.3178)	0.1909 (0.3026)	0.2197 (0.3028)	0.2256 (0.3448)	0.2064 (0.2871)	0.2004 (0.2671)
CC(work)	0.965 (0.886)	0.949 (0.730)	0.969 (0.885)	0.963 (0.824)	0.957 (0.874)	0.964 (0.873)	0.963 (0.919)
CC(free)	0.956 (0.853)	0.946 (0.507)	0.959 (0.787)	0.934 (0.764)	0.940 (0.788)	0.961 (0.799)	0.952 (0.869)
Number of non-hydrogen atoms	3187	3388	3510	3301	3177	3377	3269
macromolecules	2899	2931	2970	2922	2873	2940	2896
ligands	21	23	19	29	36	21	21
solvent	267	434	521	350	268	416	352
Protein residues	376	376	376	376	376	376	376
RMS(bonds)	0.004	0.01	0.006	0.003	0.004	0.012	0.004
RMS(angles)	0.58	0.82	0.75	0.53	0.58	0.95	0.58
Ramachandran favored (%)	97.85	98.39	98.12	97.85	97.31	97.85	98.66
Ramachandran allowed (%)	2.15	1.61	1.88	1.88	2.69	2.15	1.34
Ramachandran outliers (%)	0.00	0	0	0.27	0.00	0	0.00
Rotamer outliers (%)	0.00	0	0	1	0.34	0.66	1.00

Clashscore	1.77	1.74	1.37	2.09	3.71	2.08	3.52
Average B-factor	42.81	36.19	27.52	34.29	46.87	27.53	26.72
macromolecules	42.44	34.78	25.44	33.2	46.15	26.28	25.81
ligands	62.56	60.46	51.51	63.86	84.36	36.74	30.74
solvent	45.26	44.42	38.53	40.88	49.64	35.92	33.97

^{1}H-NMR (400 MHz, $CDCl_3$) of **4-(Chloromethyl)-7-iodo-2*H*-chromen-2-one, 3**

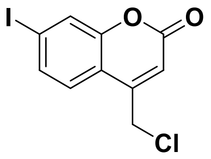

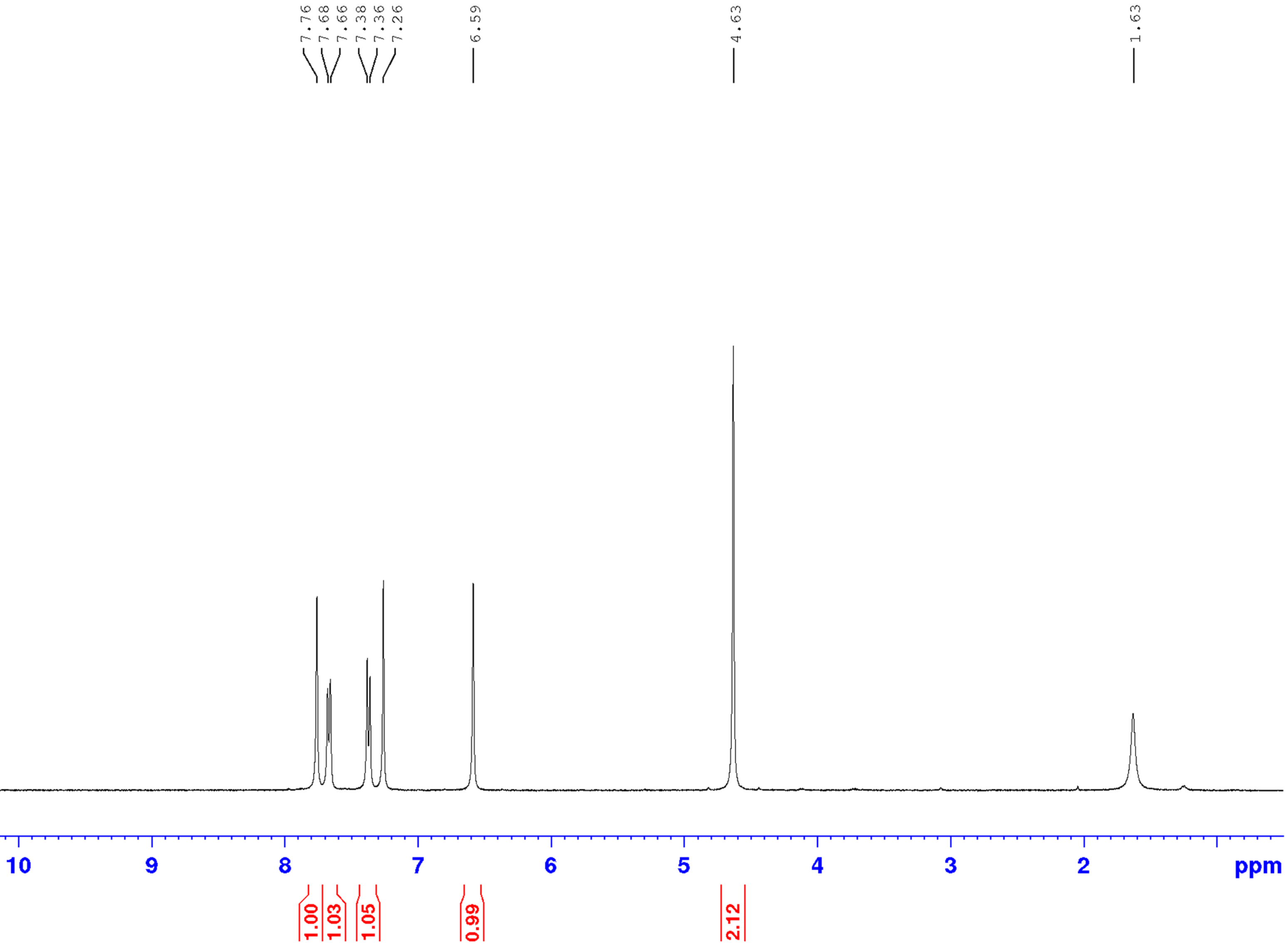

^{13}C-NMR (100 MHz, $CDCl_3$) of **4-(Chloromethyl)-7-iodo-2*H*-chromen-2-one, 3**

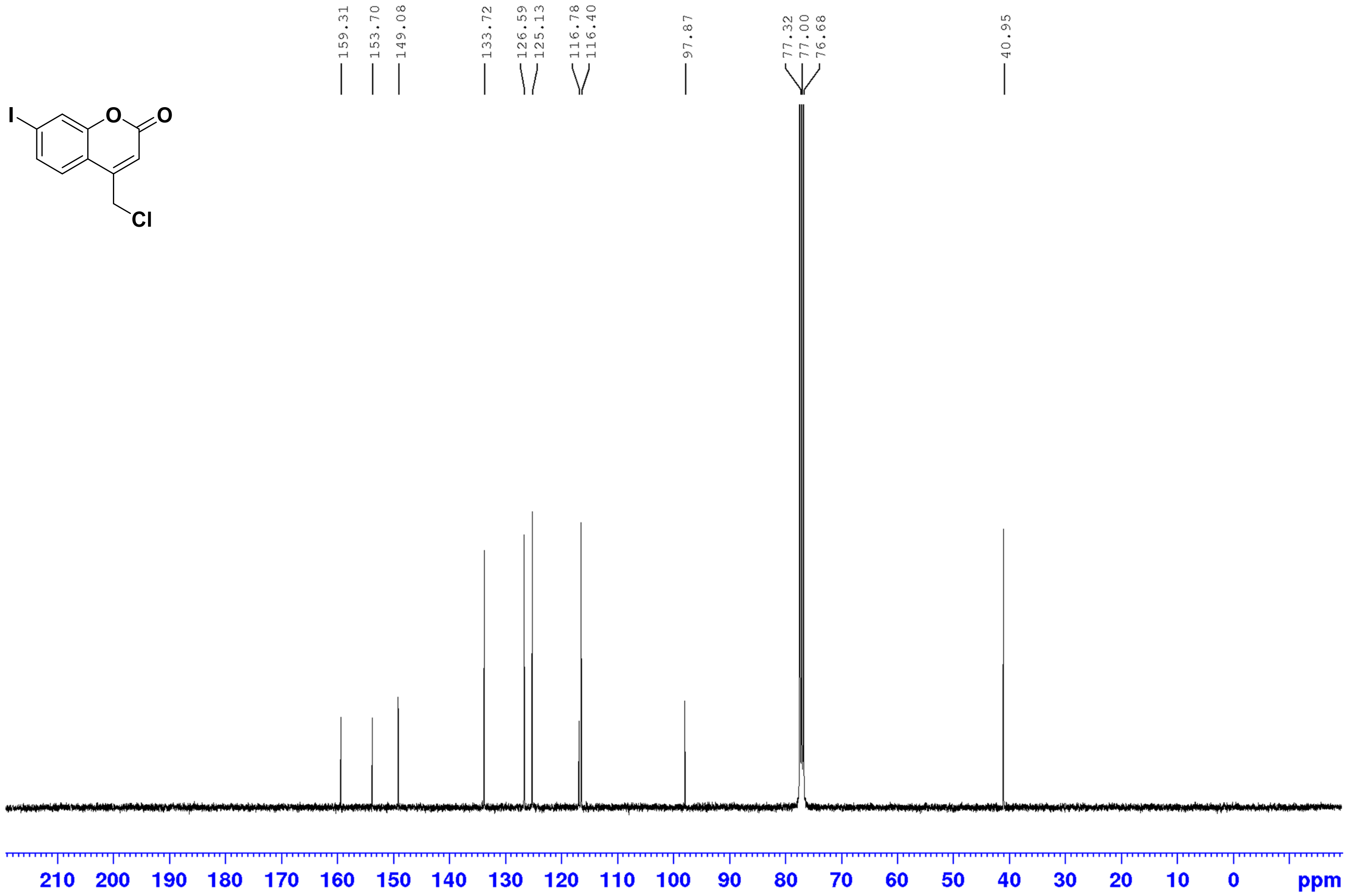

^{1}H-NMR (400 MHz, $CDCl_3$) of **2-(6-Iodobenzofuran-3-yl)acetic acid, 8**

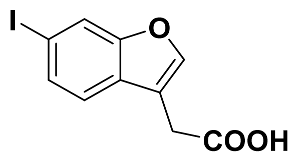

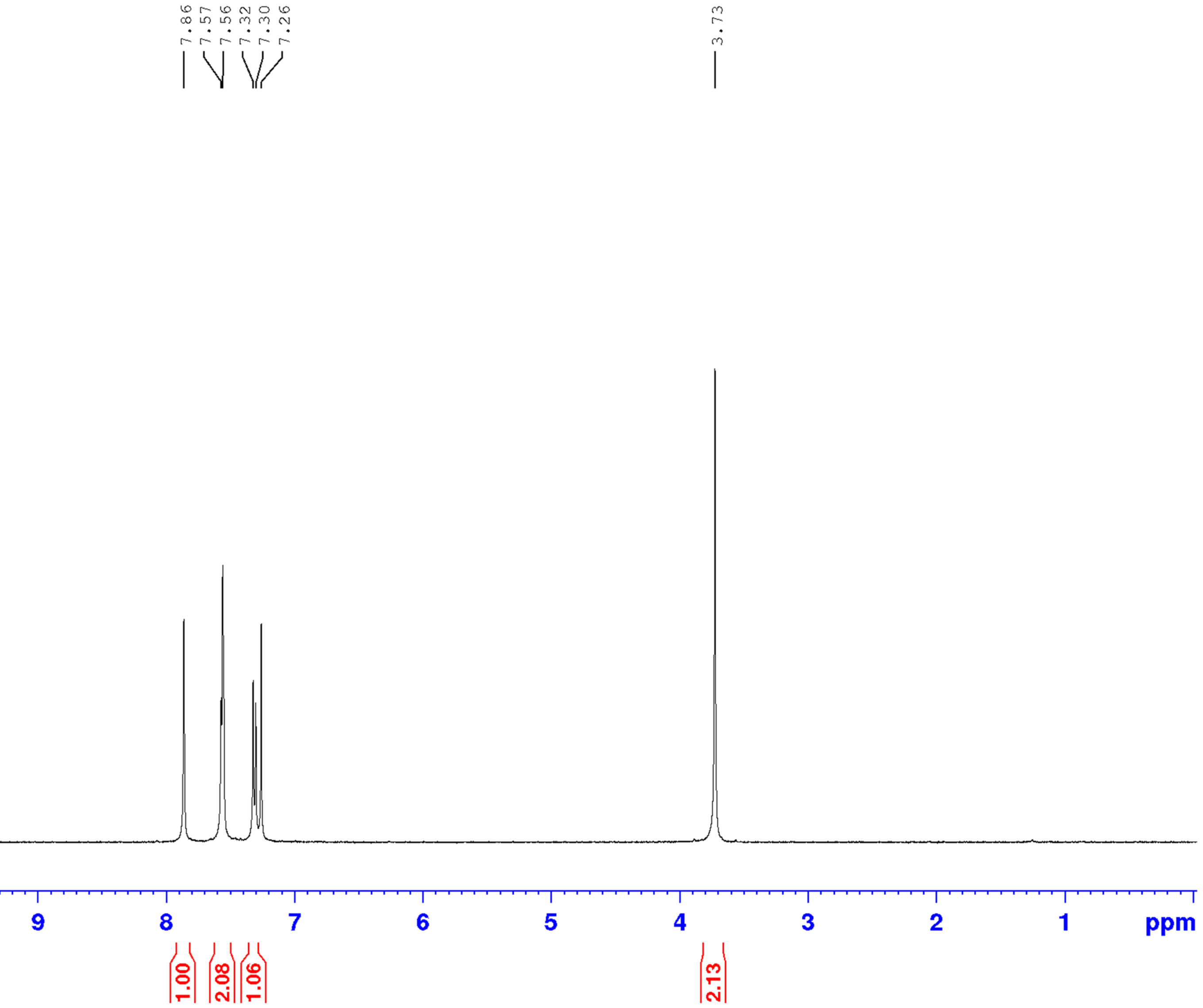

^{13}C-NMR (100 MHz, DMSO-d_6) of **2-(6-Iodobenzofuran-3-yl)acetic acid, 8**

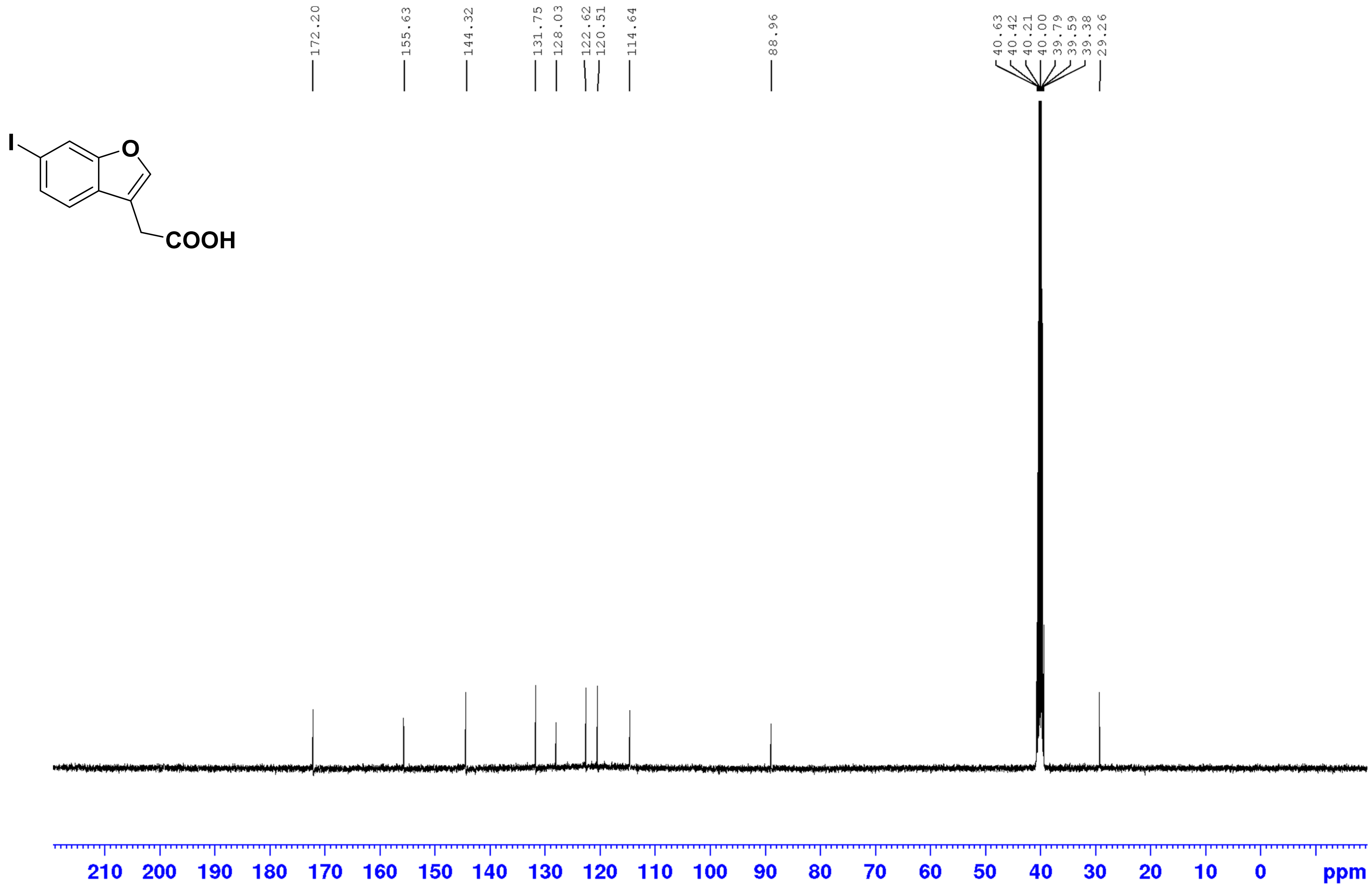

^{1}H-NMR (400 MHz, $CDCl_3$) **Methyl 2-(6-bromobenzofuran-3-yl)acetate, 11**

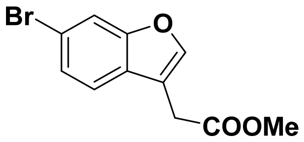

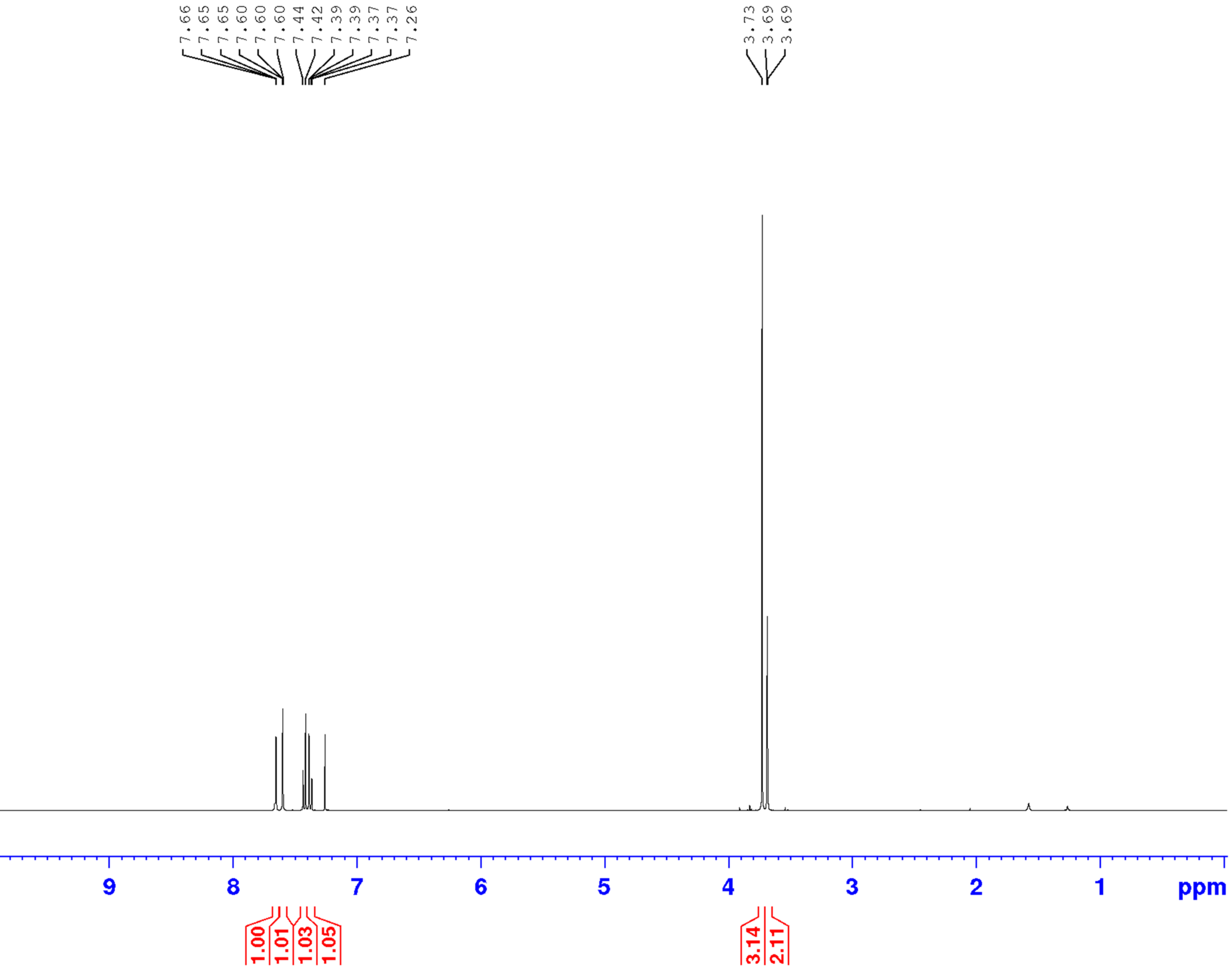

^{13}C-NMR (100 MHz, $CDCl_3$) of **Methyl 2-(6-bromobenzofuran-3-yl)acetate, 11**

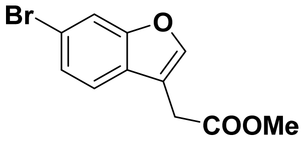

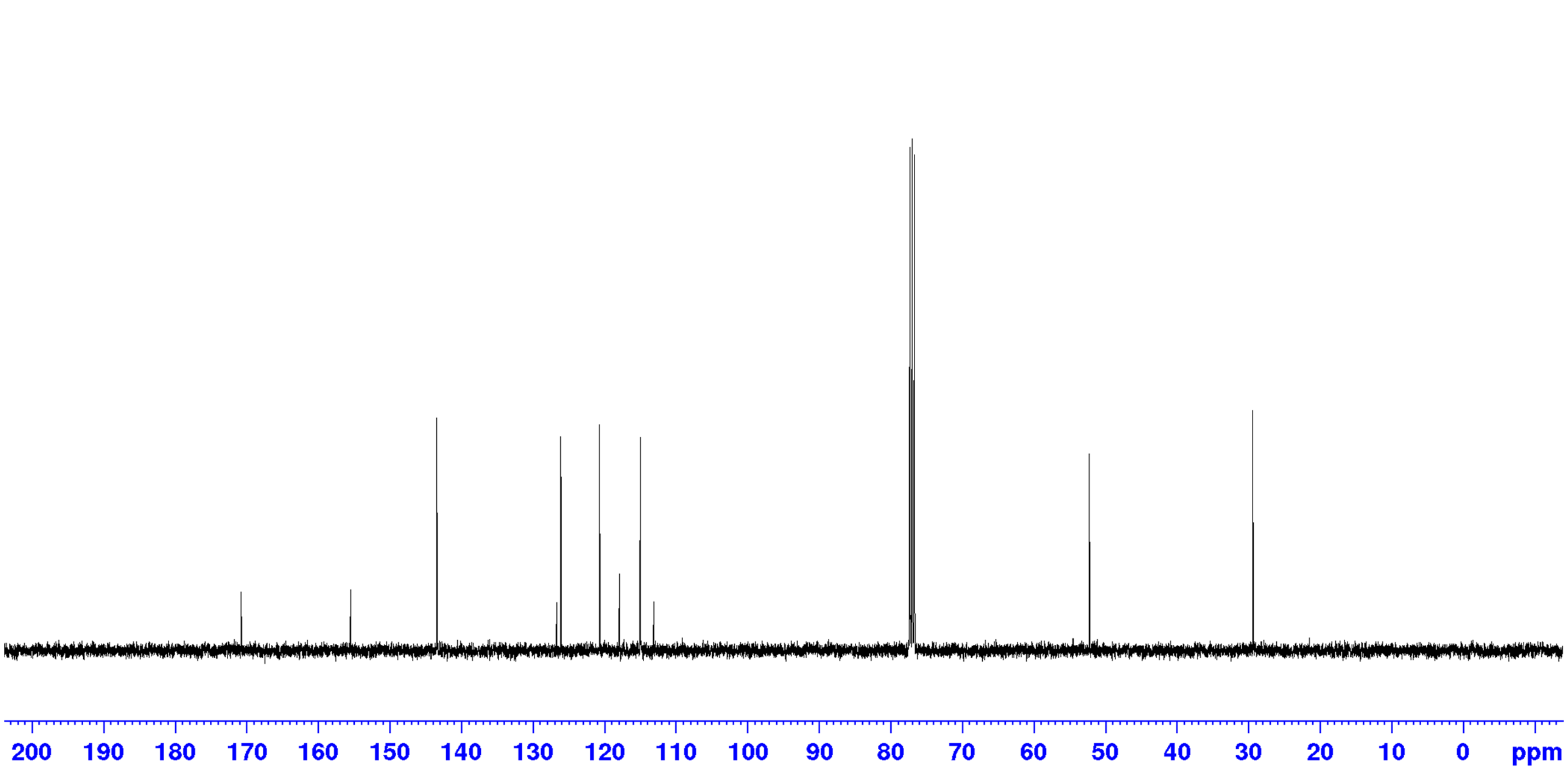

^{1}H-NMR (400 MHz, $CDCl_3$) of **Methyl 2-(6-Iodobenzofuran-3-yl)acetate, 12**

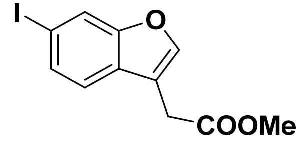

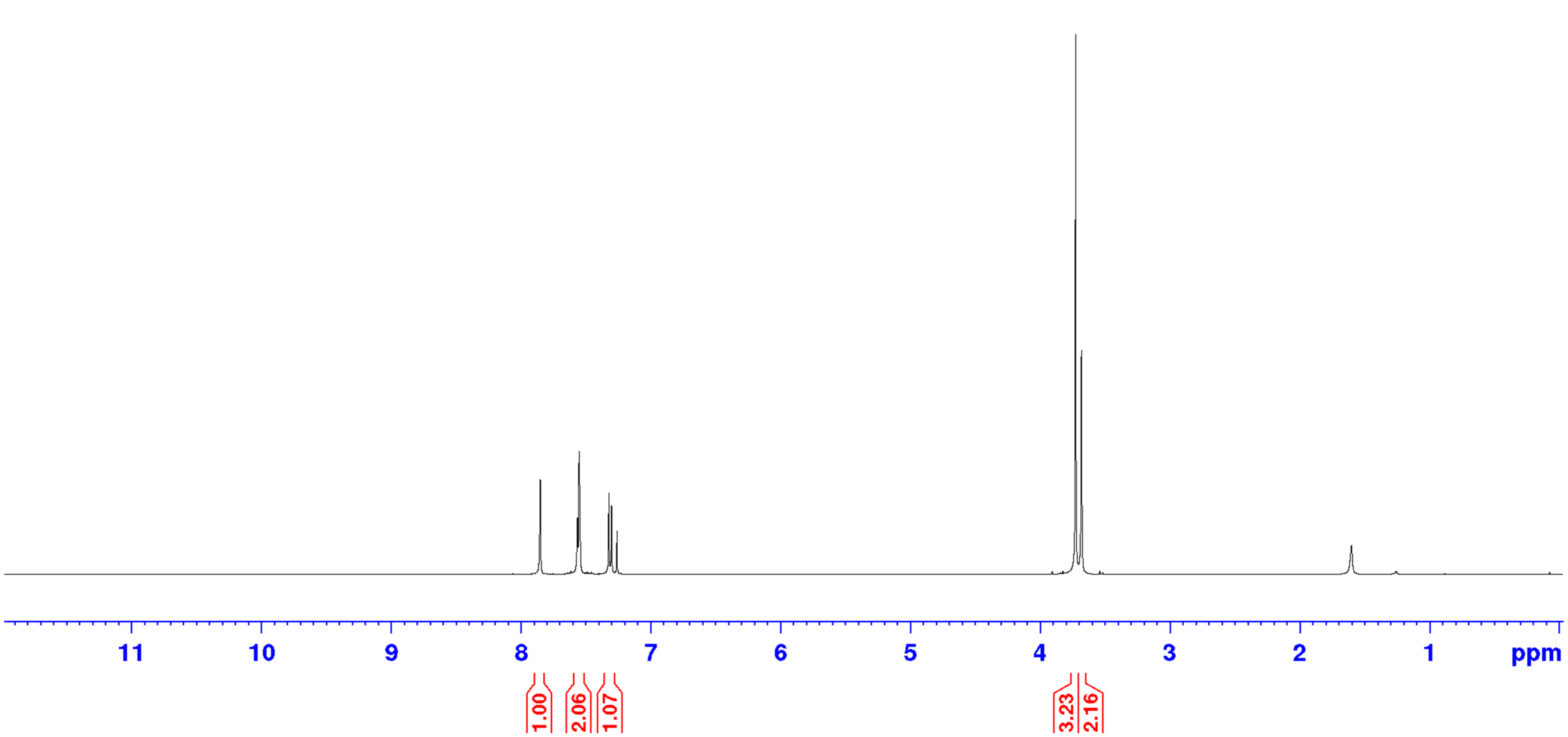

^{13}C-NMR (100 MHz, $CDCl_3$) of **Methyl 2-(6-Iodobenzofuran-3-yl)acetate, 12**

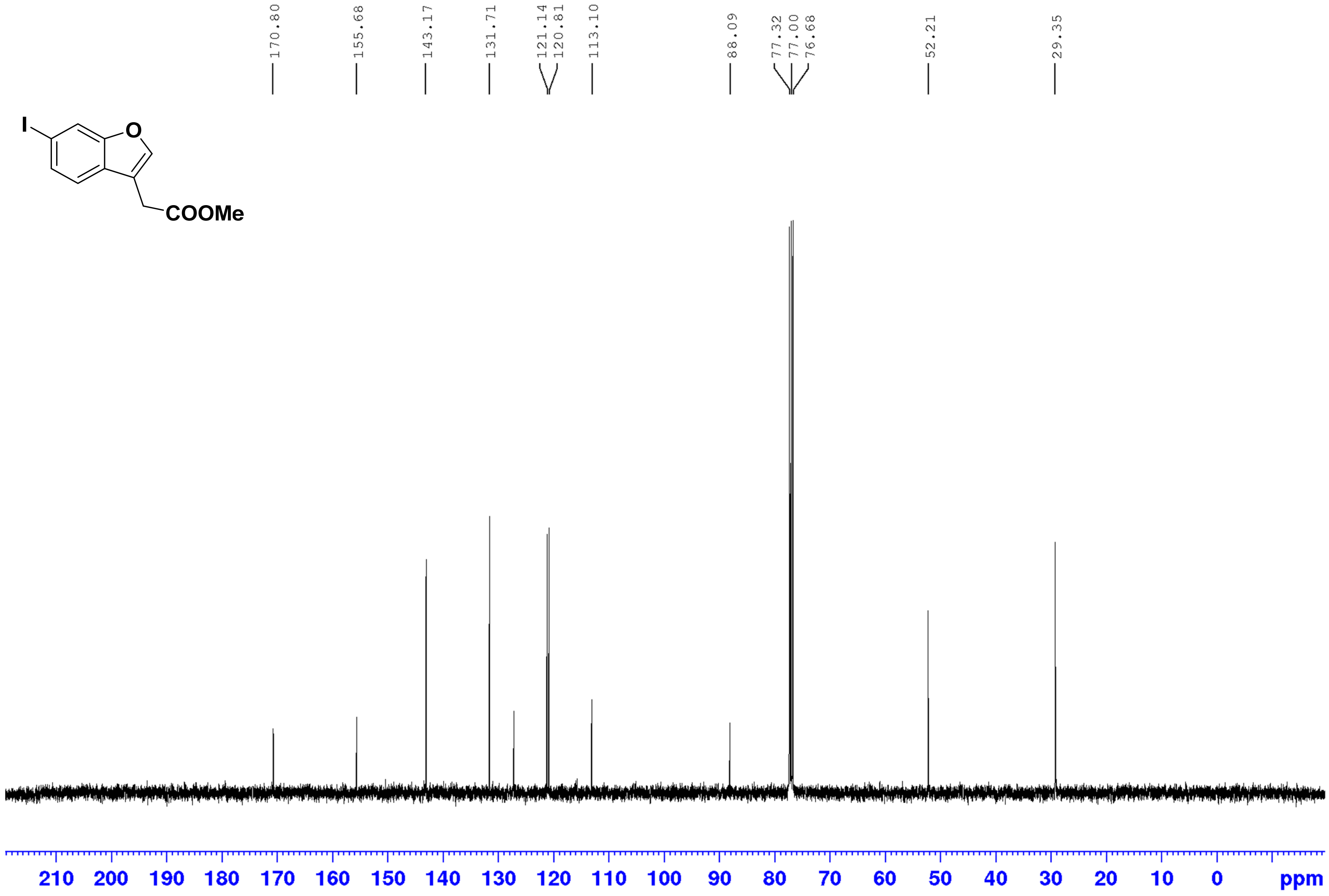

^{1}H-NMR (400 MHz, $CDCl_3$) of **2-(6-(Phenylamino)benzofuran-3-yl)acetic acid, 15**

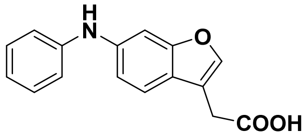

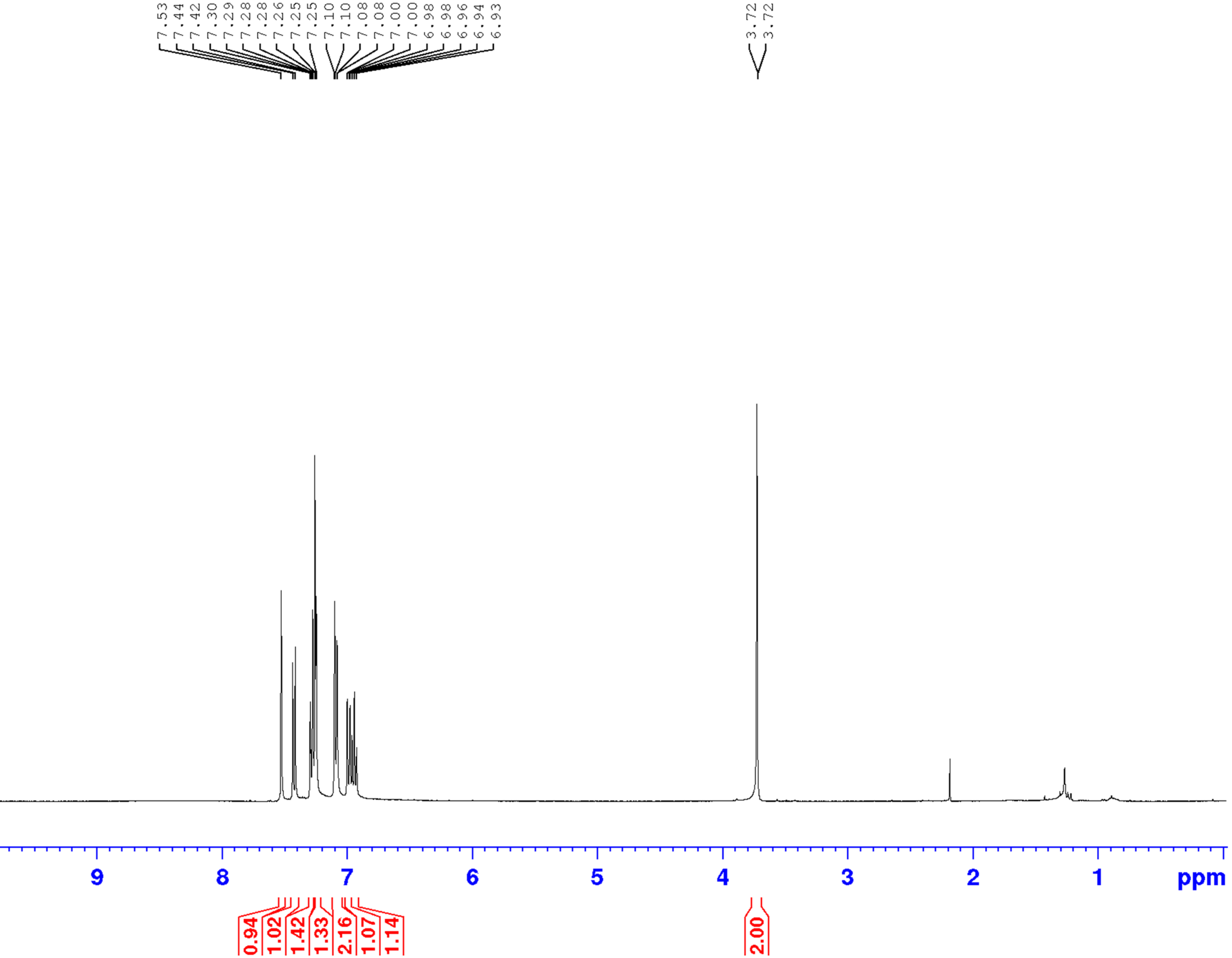

^{13}C-NMR (125 MHz, $CDCl_3$) of **2-(6-(Phenylamino)benzofuran-3-yl)acetic acid, 15**

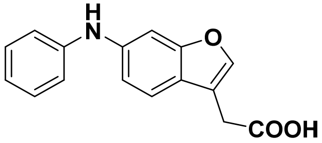

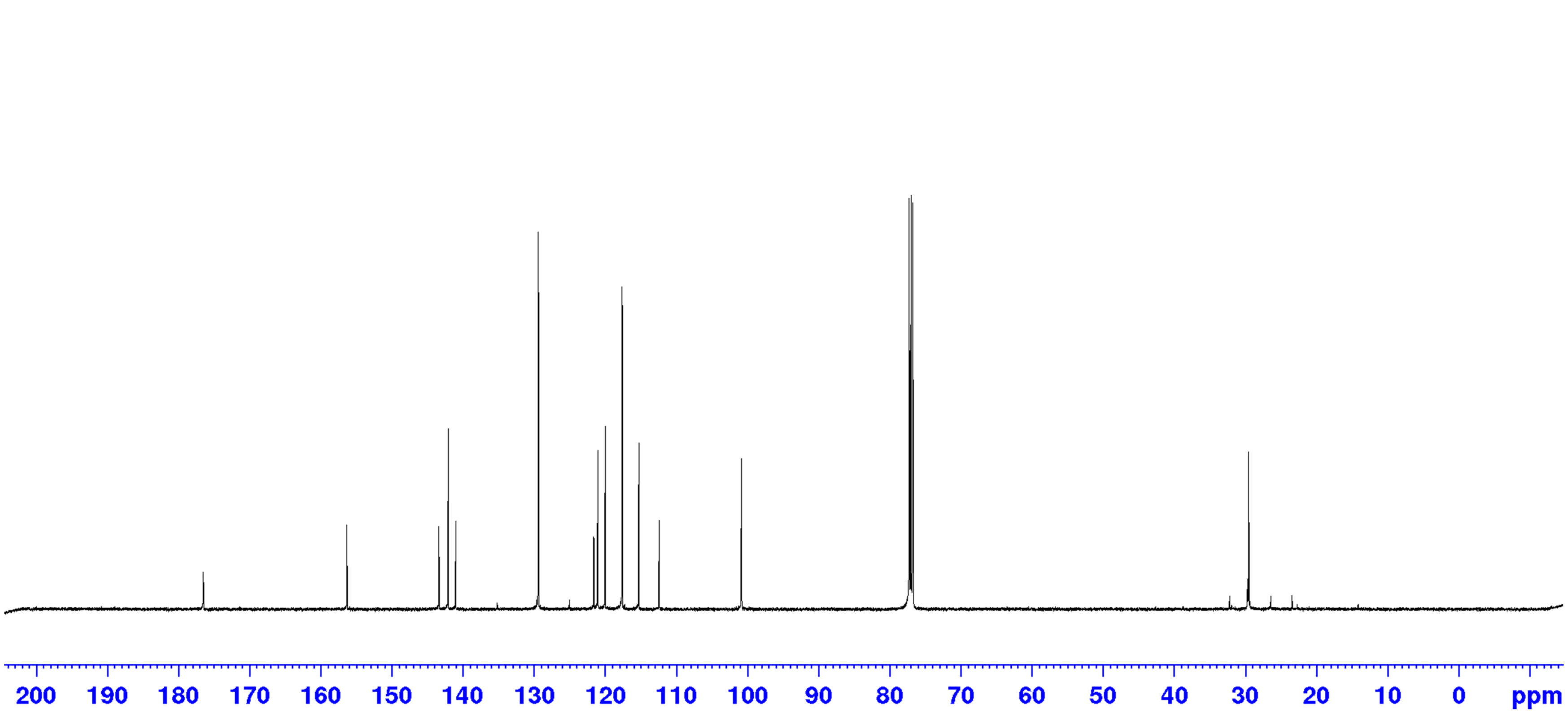

^{1}H-NMR (400 MHz, $CDCl_3$) of **2-(6-((4-Methoxyphenyl)amino)benzofuran-3-yl)acetic acid, 16**

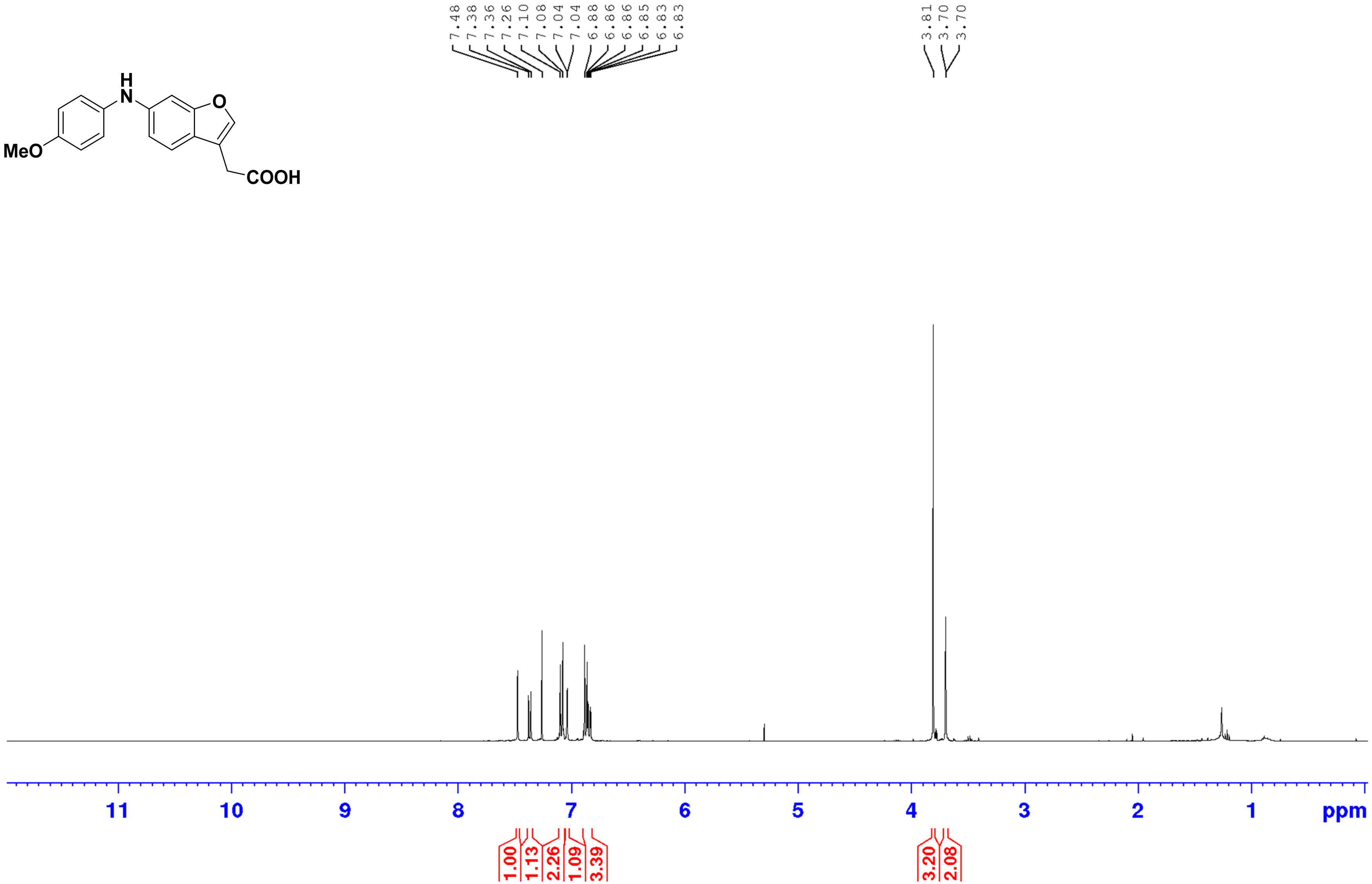

^{13}C-NMR (100 MHz, $CDCl_3$) of **2-(6-((4-Methoxyphenyl)amino)benzofuran-3-yl)acetic acid, 16**

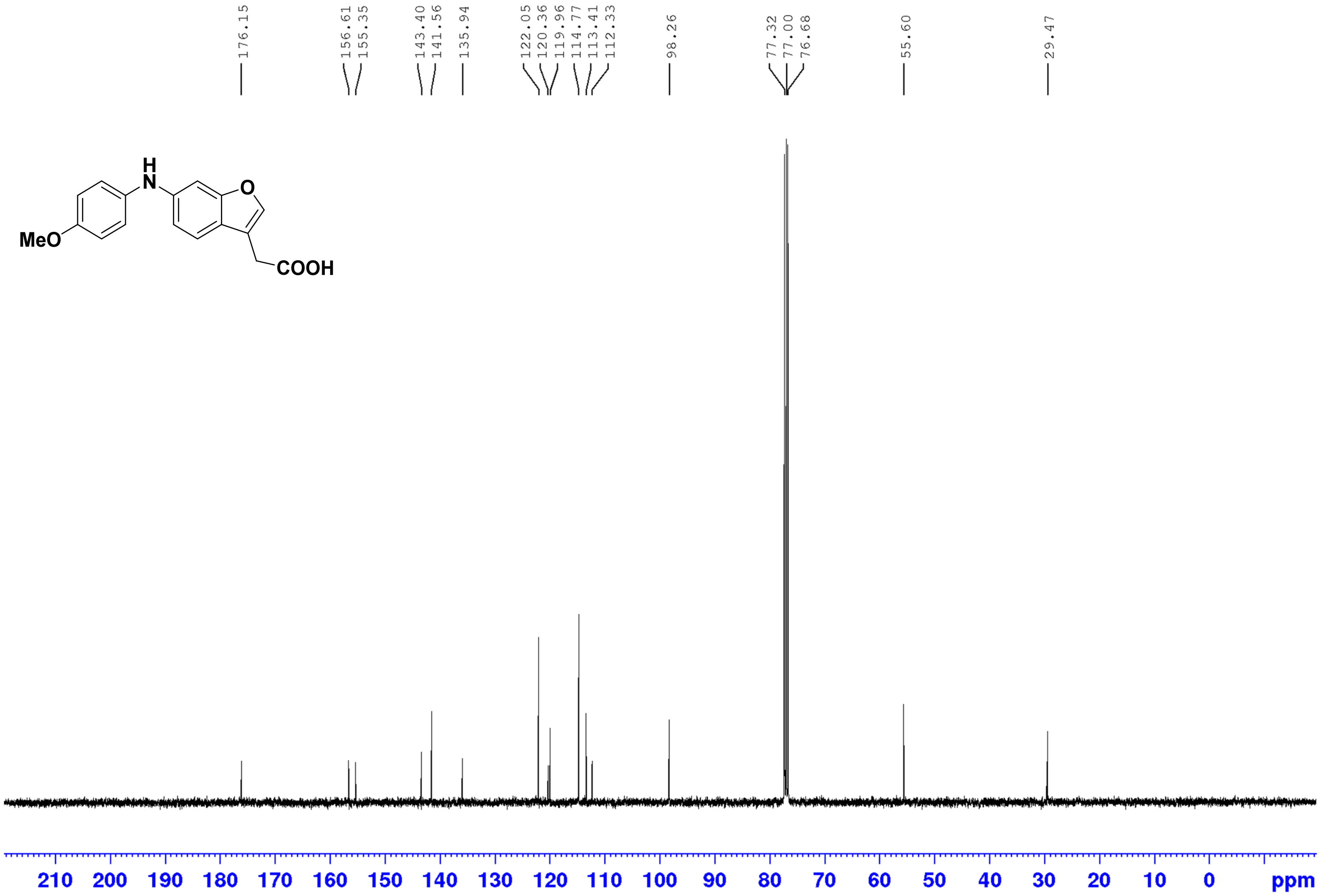

^{1}H-NMR (400 MHz, $CDCl_3$) of **Isobutyl 2-(6-iso butoxybenzofuran-3-yl)acetate, 18**

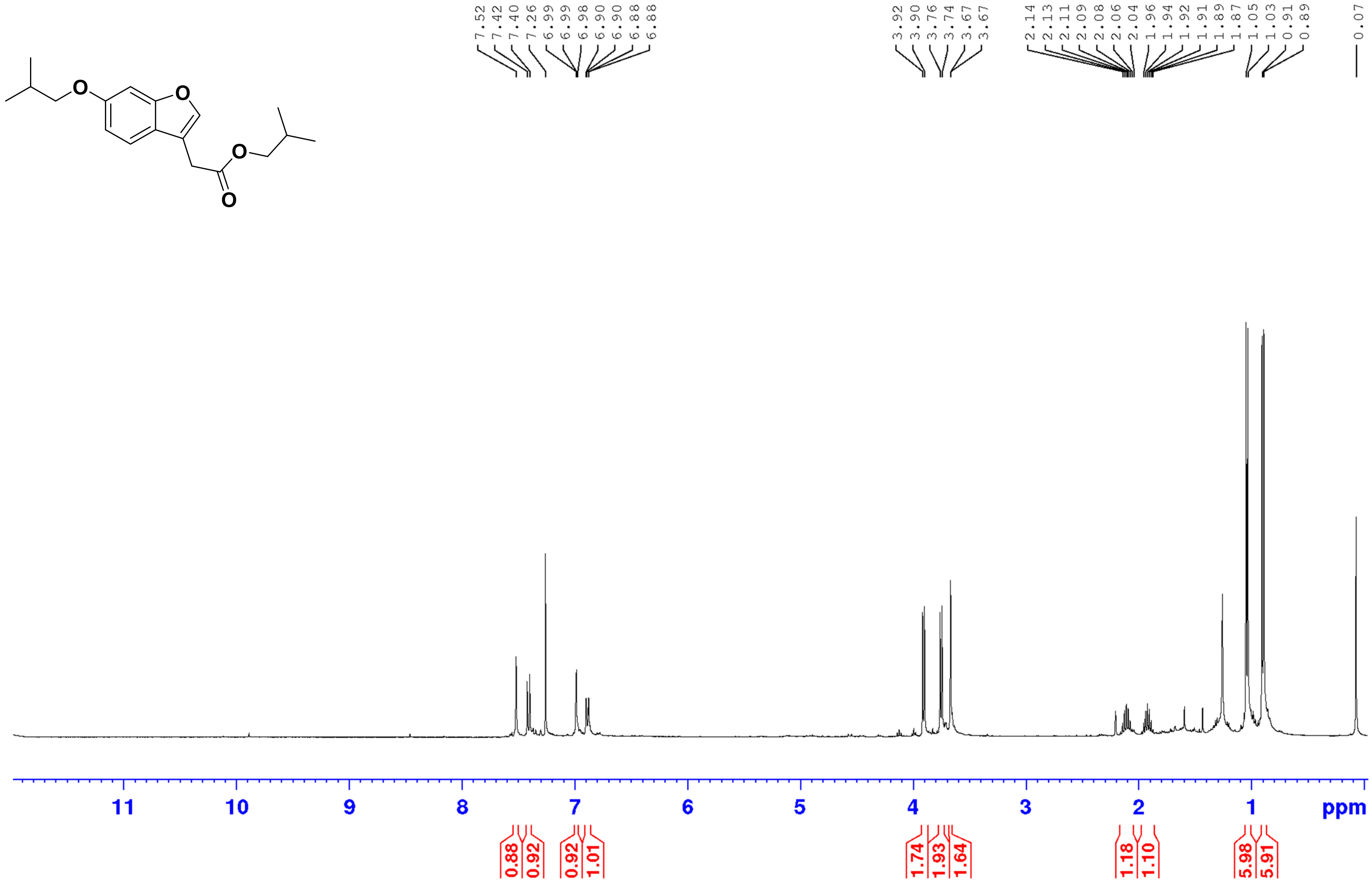

^{13}C-NMR (100 MHz, $CDCl_3$) of **Isobutyl 2-(6-iso butoxybenzofuran-3-yl)acetate, 18**

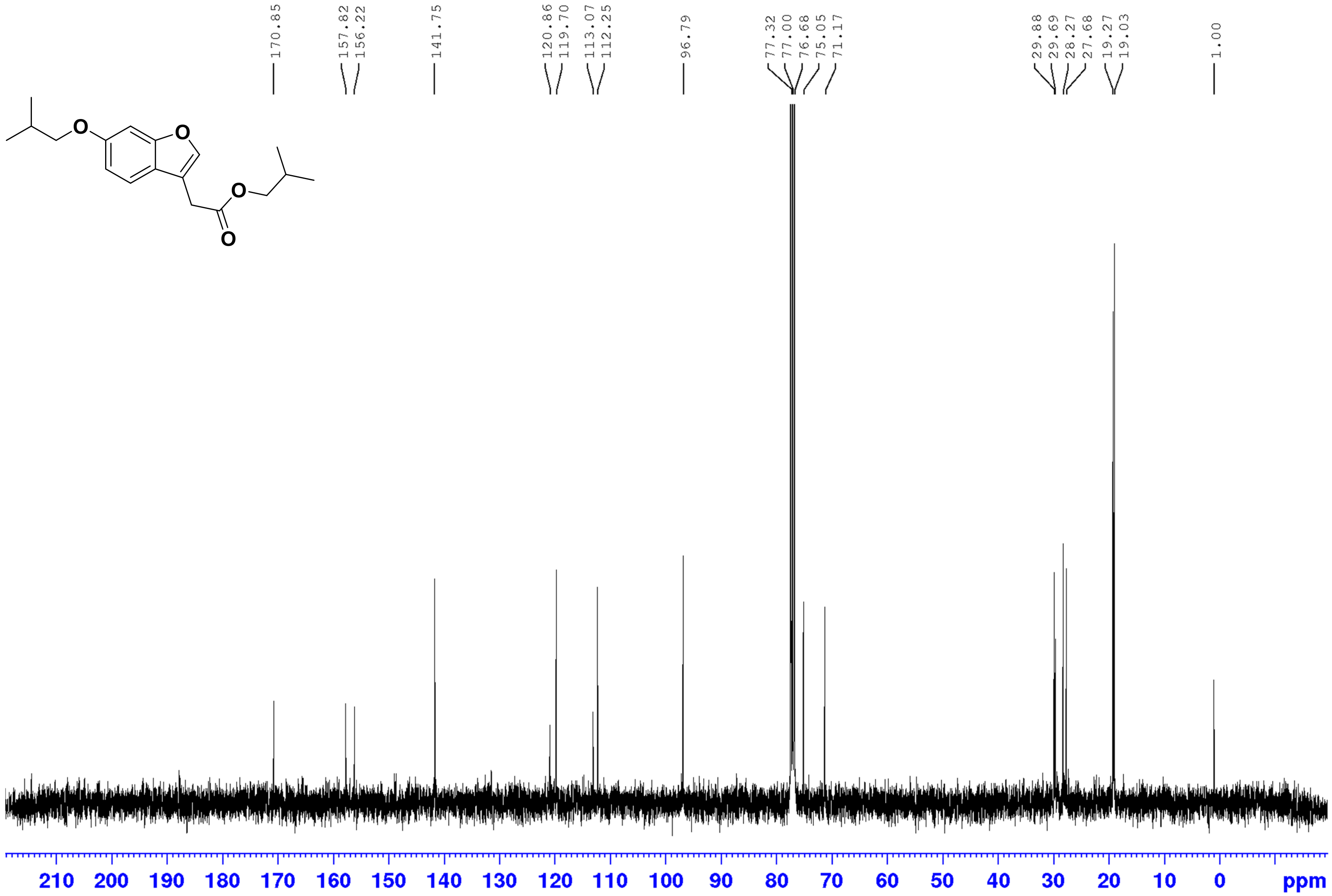

[11]1H-NMR (400 MHz, $CDCl_3$) of **Benzyl 2-(6-(benzyloxyl)benzofuran-3-yl)acetate, 19**

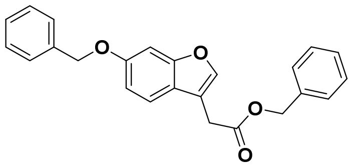

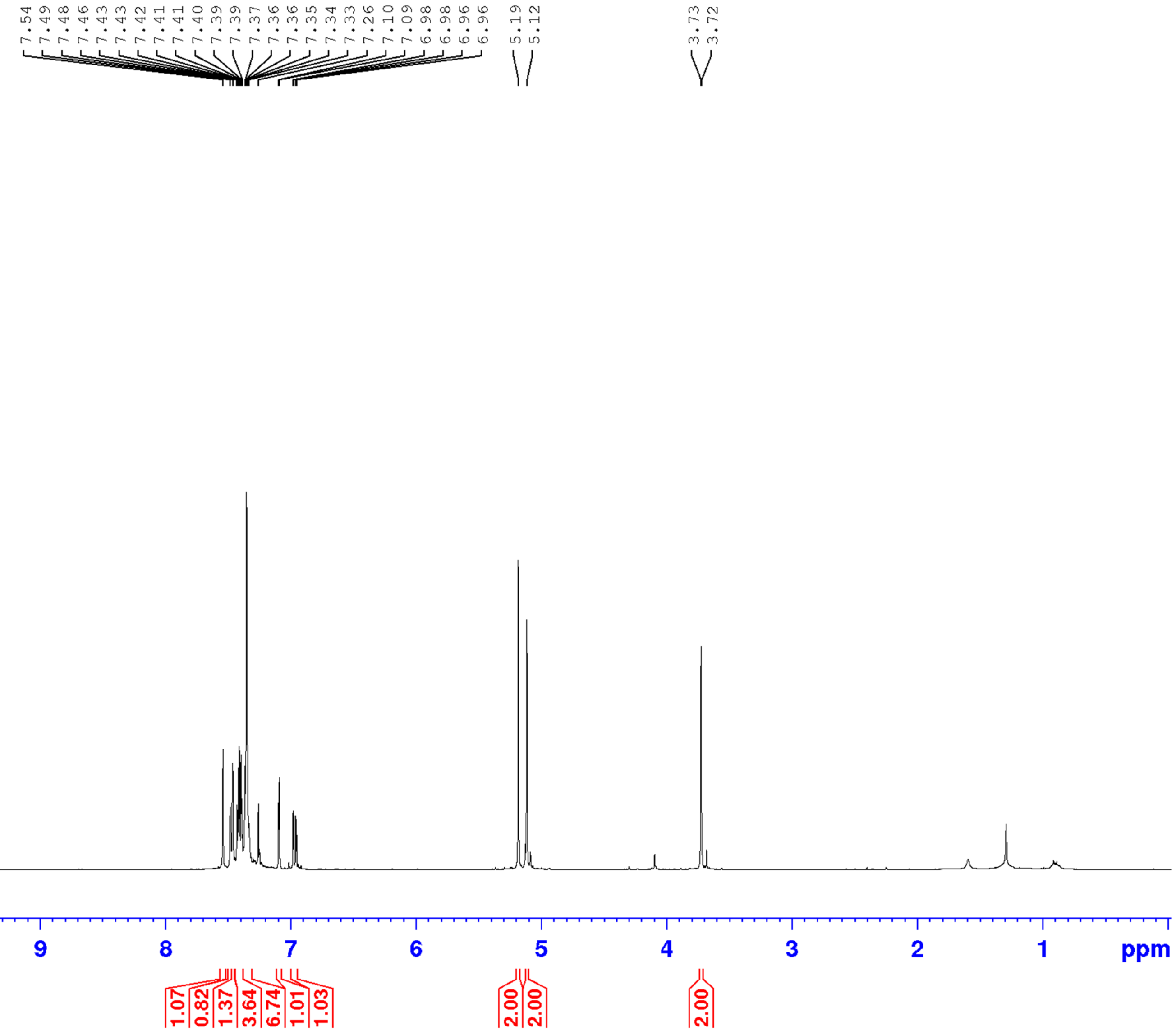

^{13}C-NMR (100 MHz, $CDCl_3$) of **Benzyl 2-(6-(benzyloxyl)benzofuran-3-yl)acetate, 19**

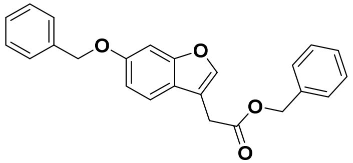

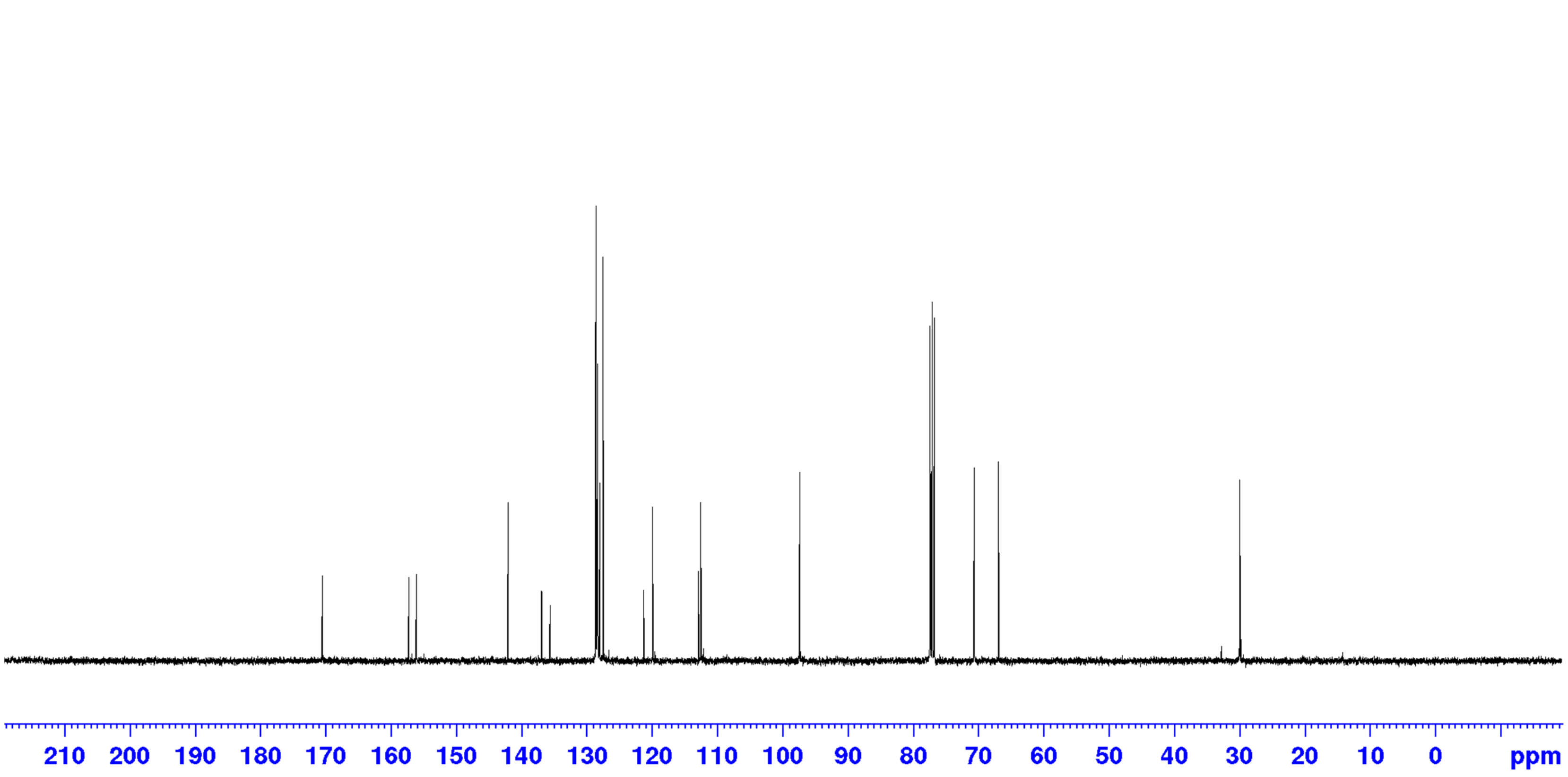

^{1}H-NMR (400 MHz, $CDCl_3$) of **Benzyl 2-(5-benzyloxy)benzofuran-3-yl)acetate, 20**

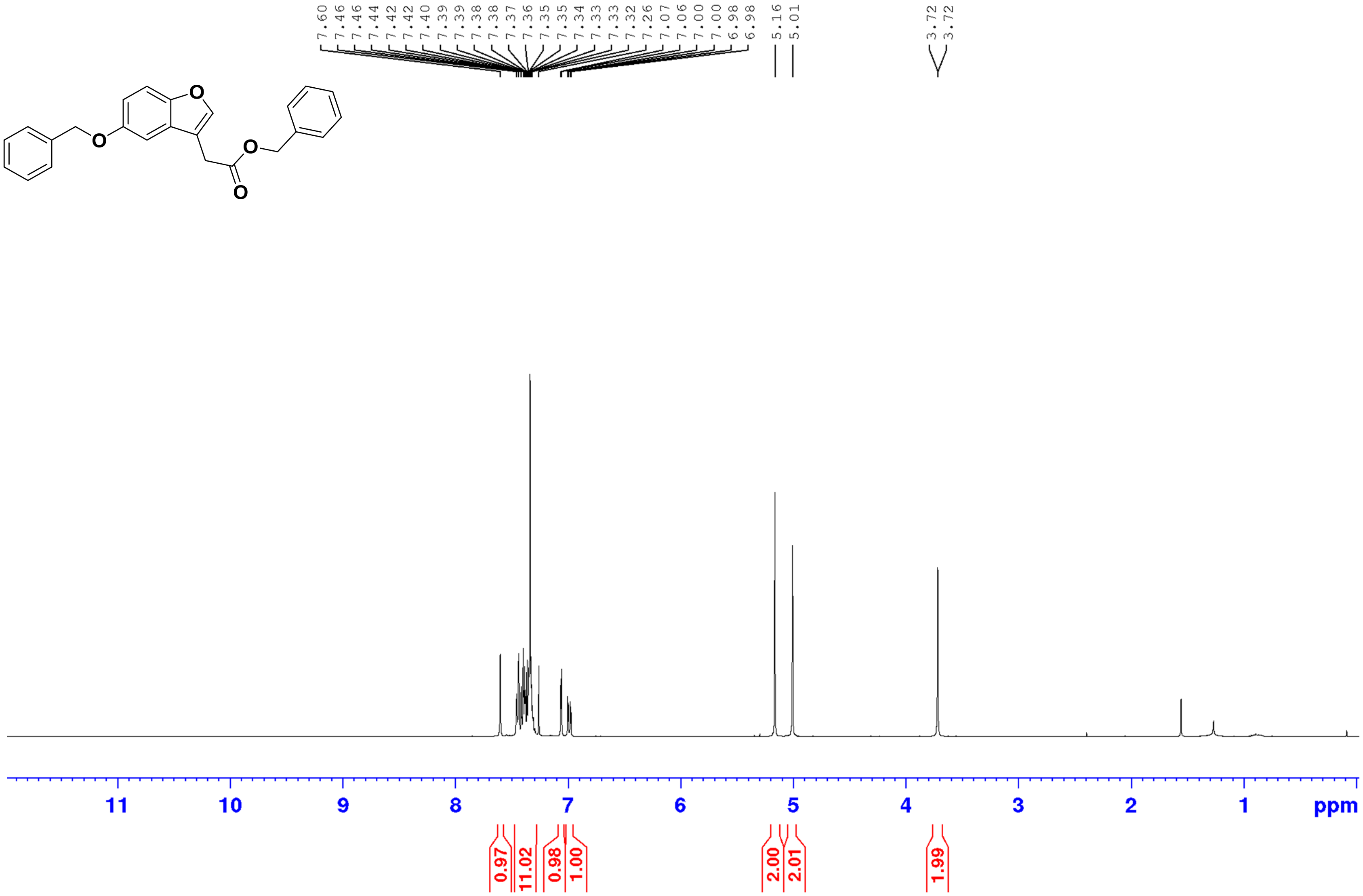

^{13}C-NMR (100 MHz, $CDCl_3$) of **Benzyl 2-(5-benzyloxy)benzofuran-3-yl)acetate, 20**

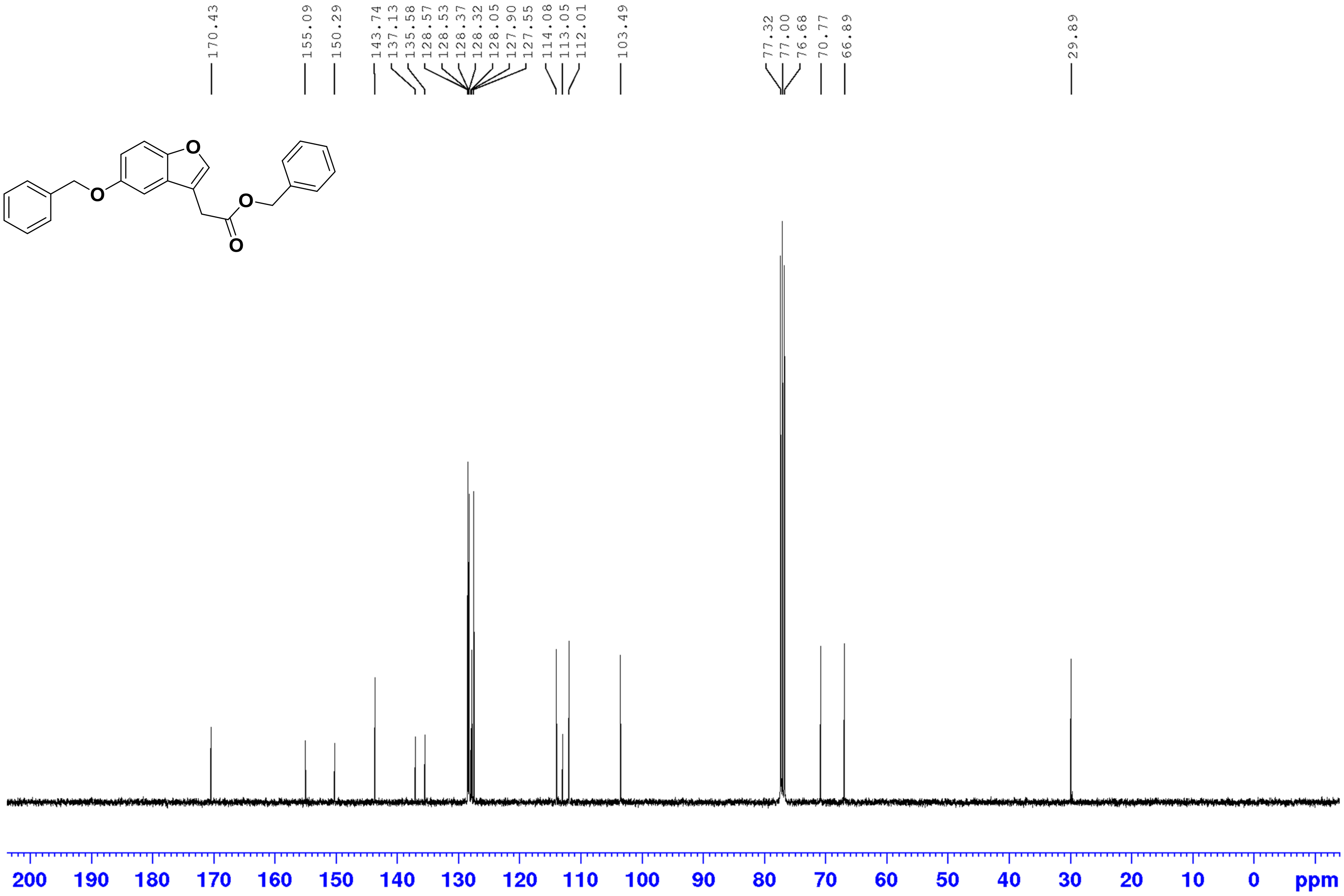

^{1}H-NMR (500 MHz, $CDCl_3$) of **2-(6-Butoxybenzofuran-3-yl)acetic acid, 21**

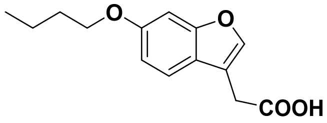

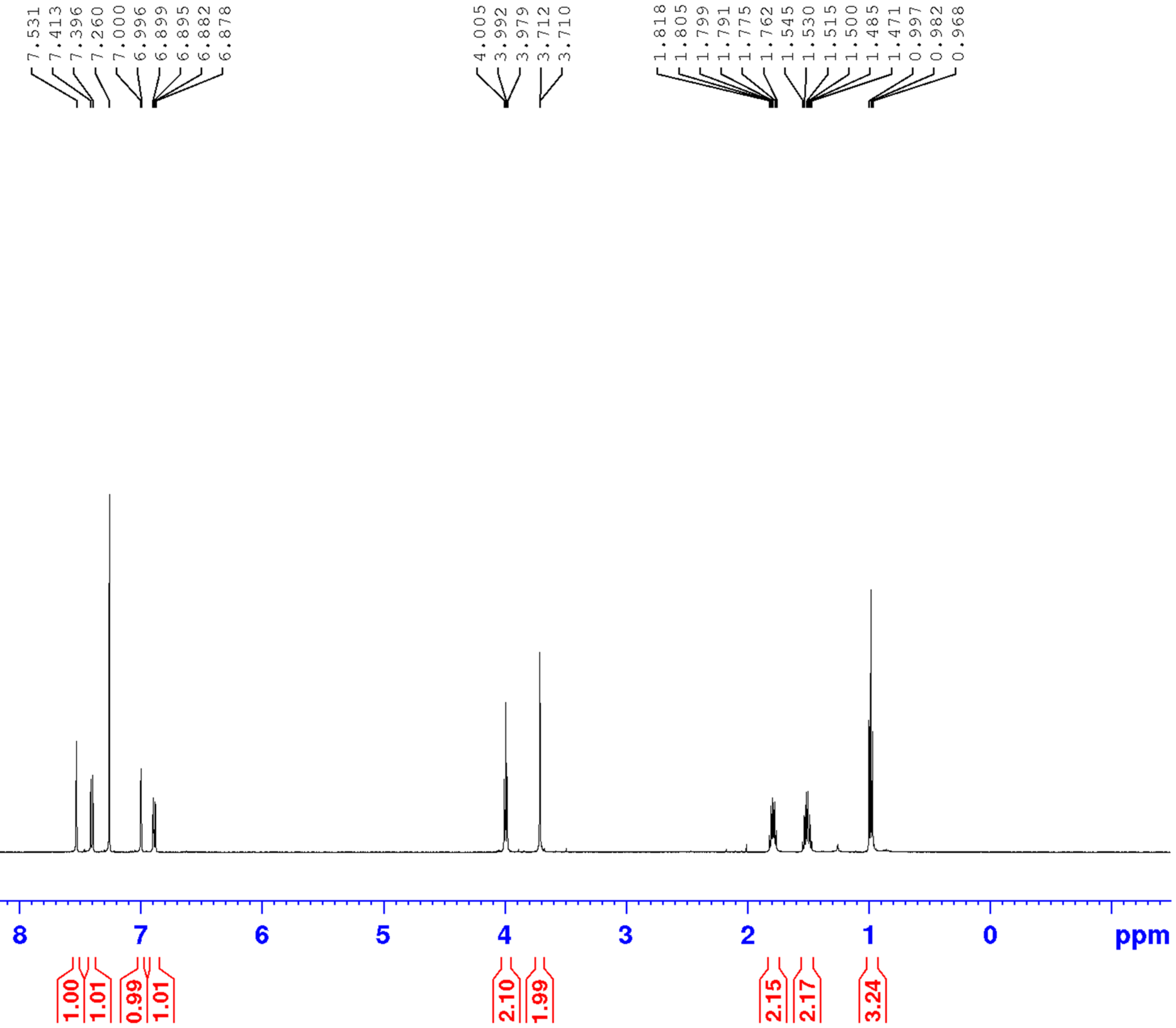

^{13}C-NMR (100 MHz, $CDCl_3$) of **2-(6-Butoxybenzofuran-3-yl)acetic acid, 21**

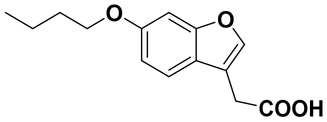

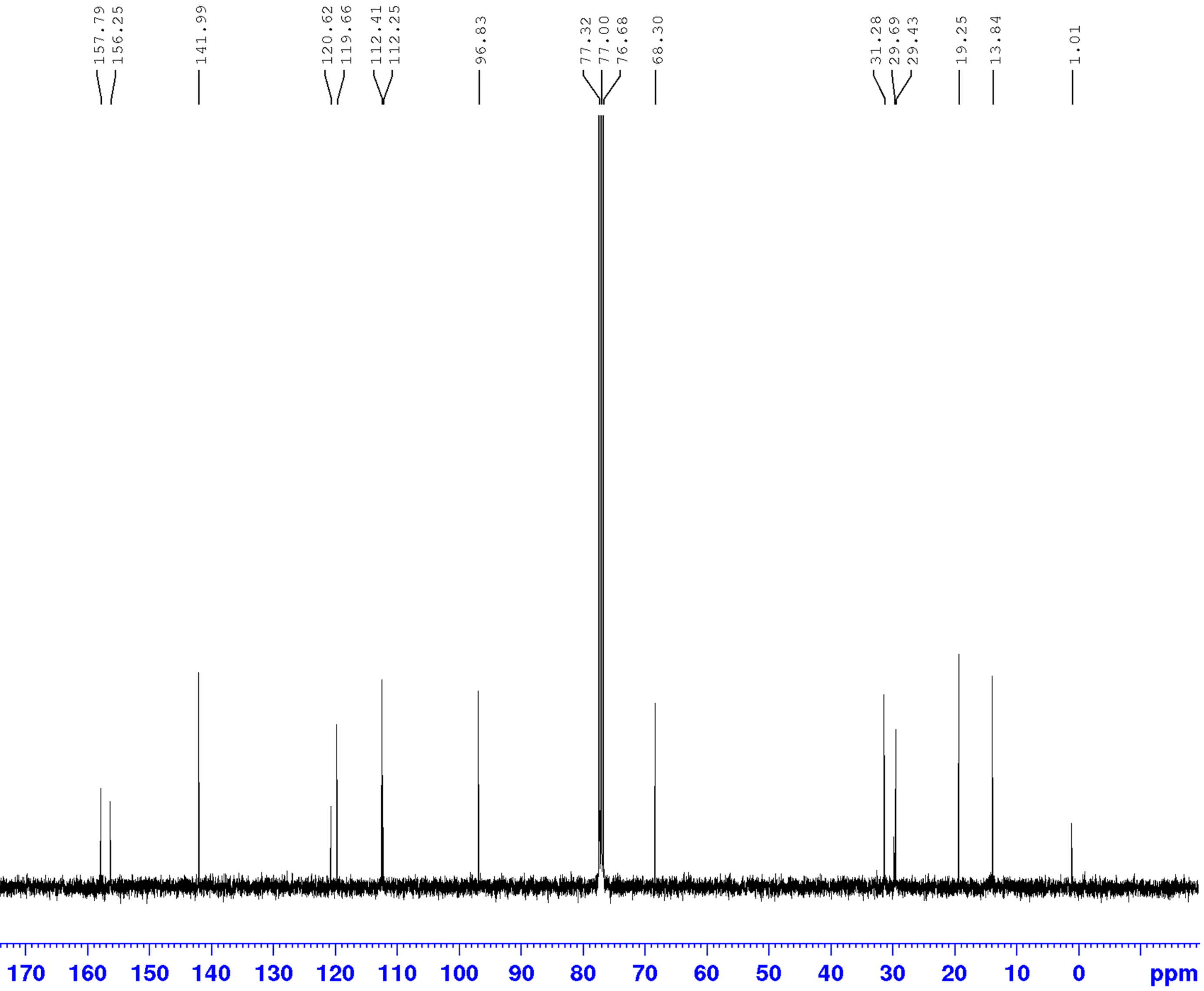

^{1}H-NMR (400 MHz, $CDCl_3$) of **2-(6-Isobutoxybenzofuran-3-yl)acetic acid, 22**

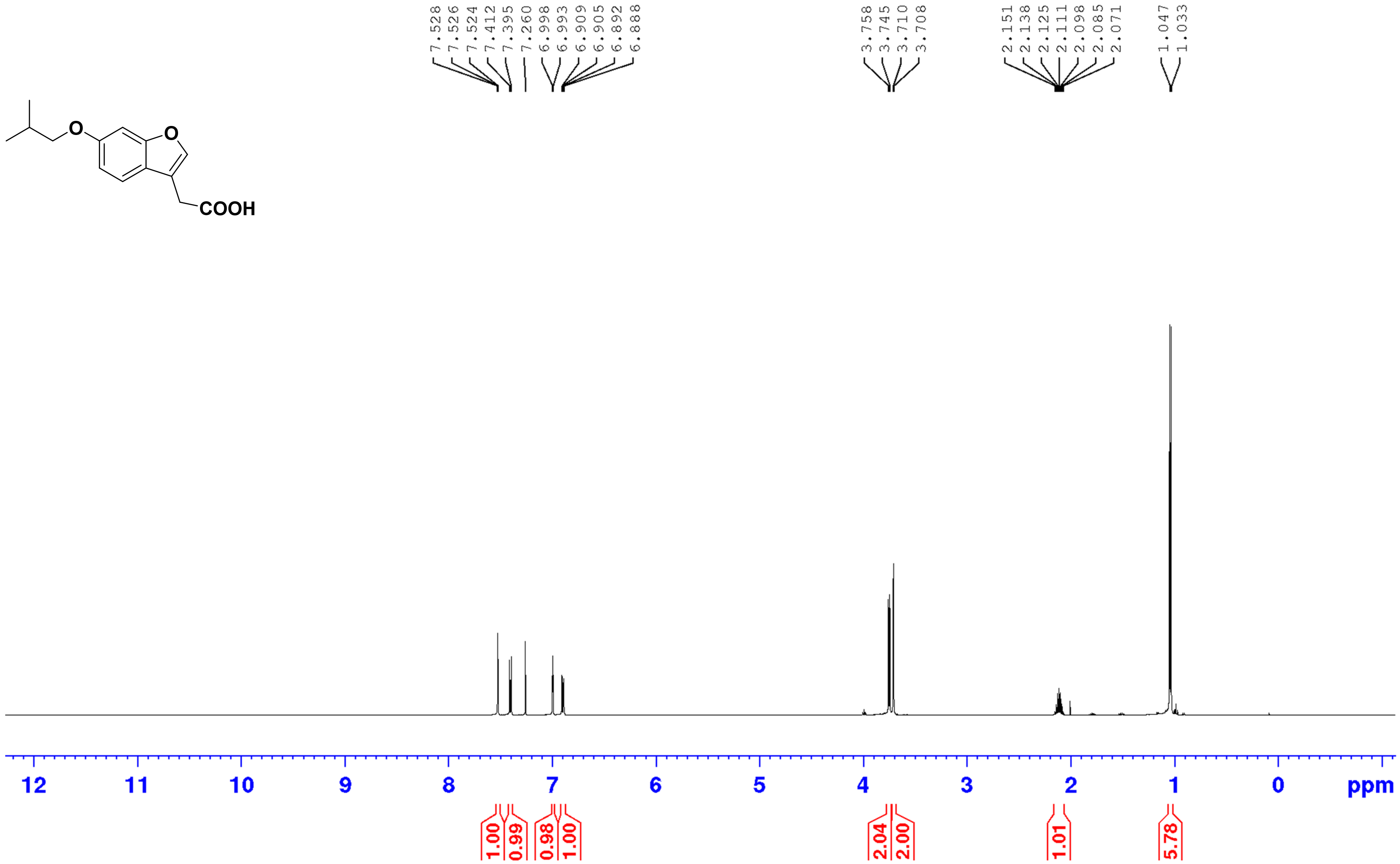

^{13}C-NMR (100 MHz, $CDCl_3$) of **2-(6-Isobutoxybenzofuran-3-yl)acetic acid, 22**

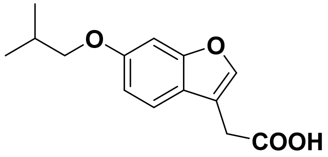

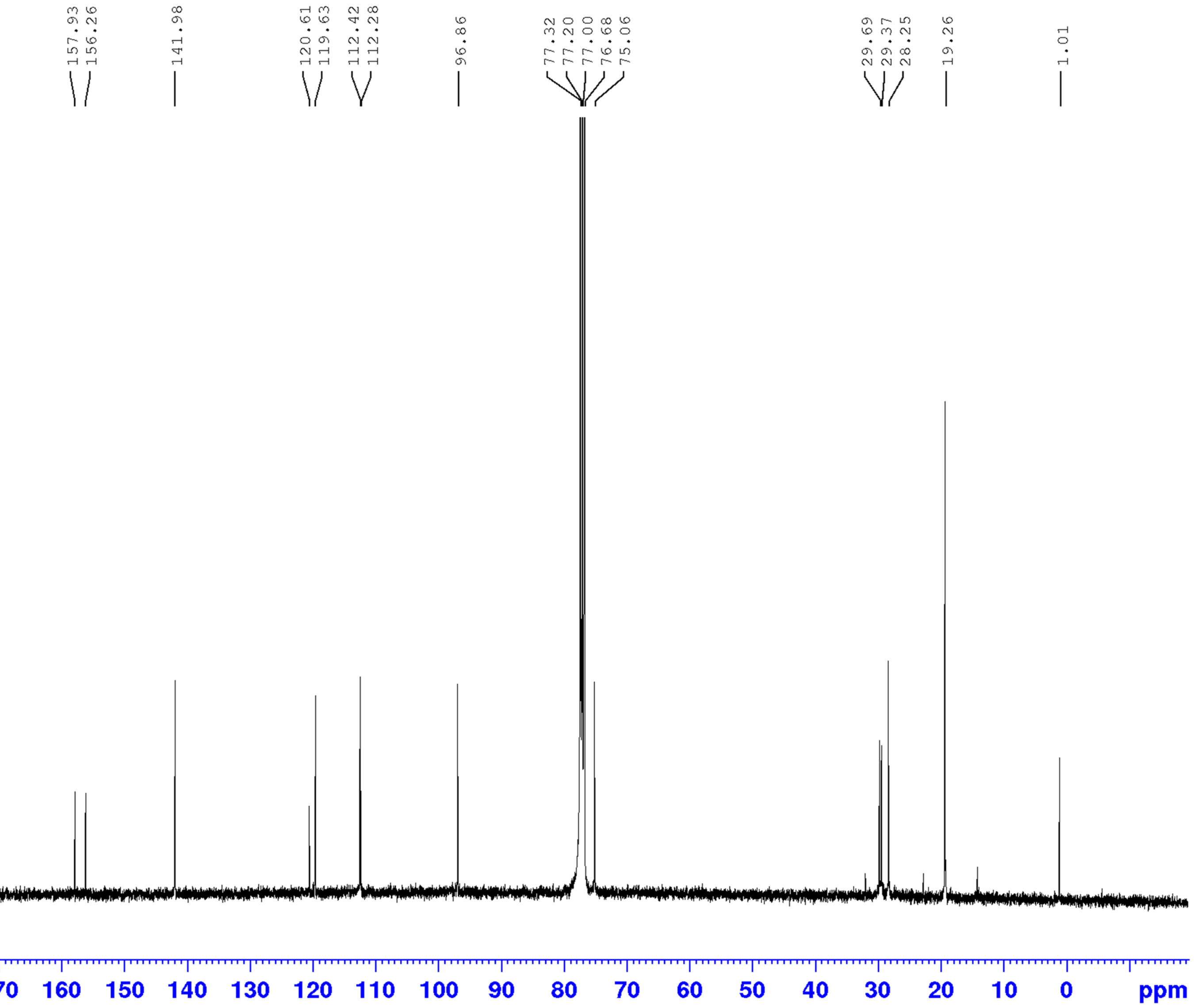

^{1}H-NMR (500 MHz, $CDCl_3$) of **2-(6-(Benzylfuran-3-yl)acetic acid, 23**

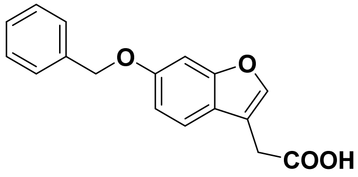

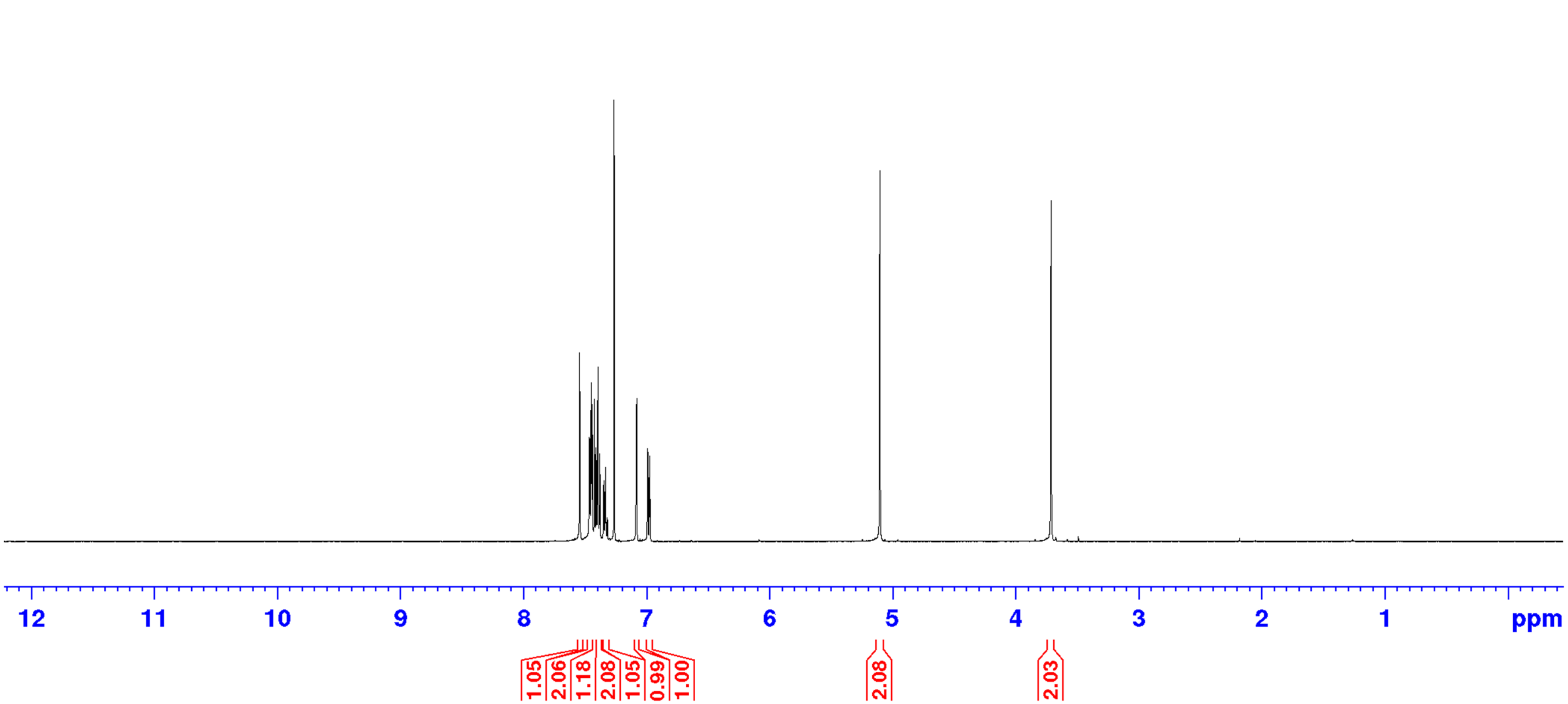

^{13}C-NMR (100 MHz, $CDCl_3$) of **2-(6-(Benzylfuran-3-yl)acetic acid, 23**

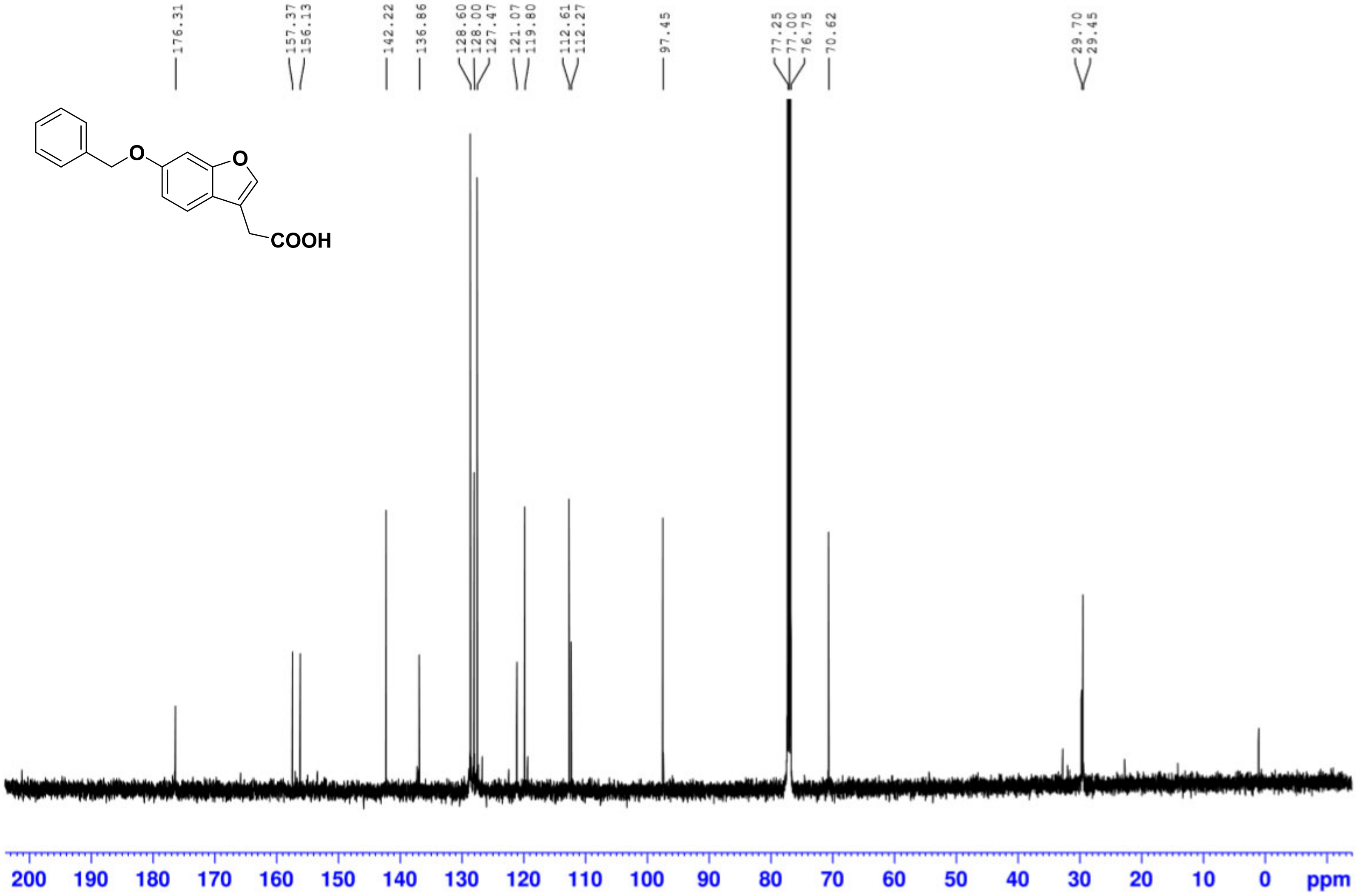

^{1}H-NMR (400 MHz, $CDCl_3$) of **2-(5-(Benzyloxy)benzofuran-3-yl)acetic acid, 24**

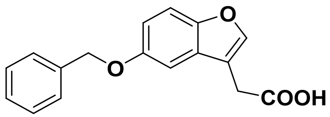

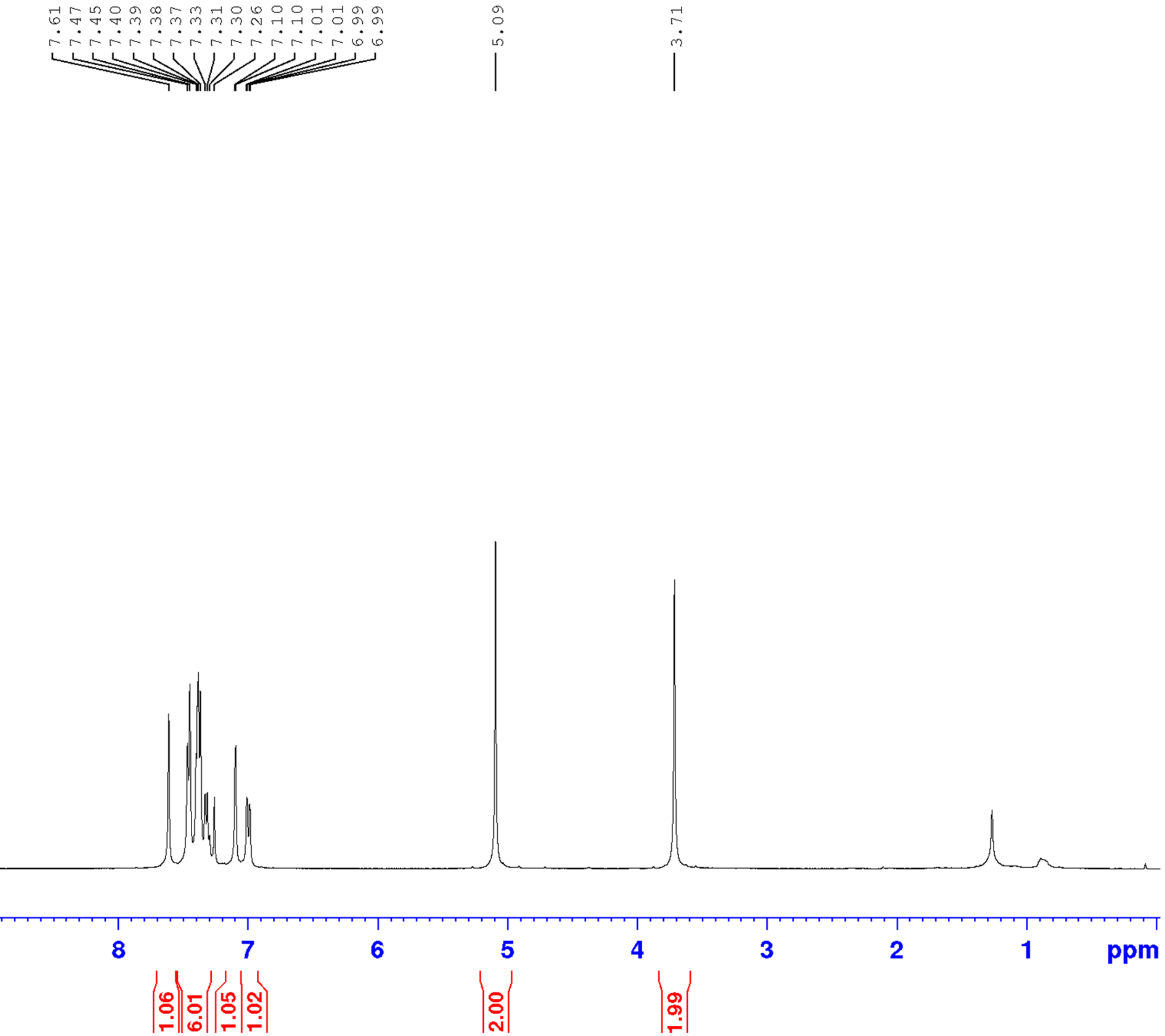

^{13}C-NMR (100 MHz, $CDCl_3$) of **2-(5-(Benzyloxy)benzofuran-3-yl)acetic acid, 24**

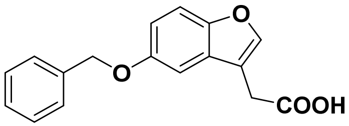

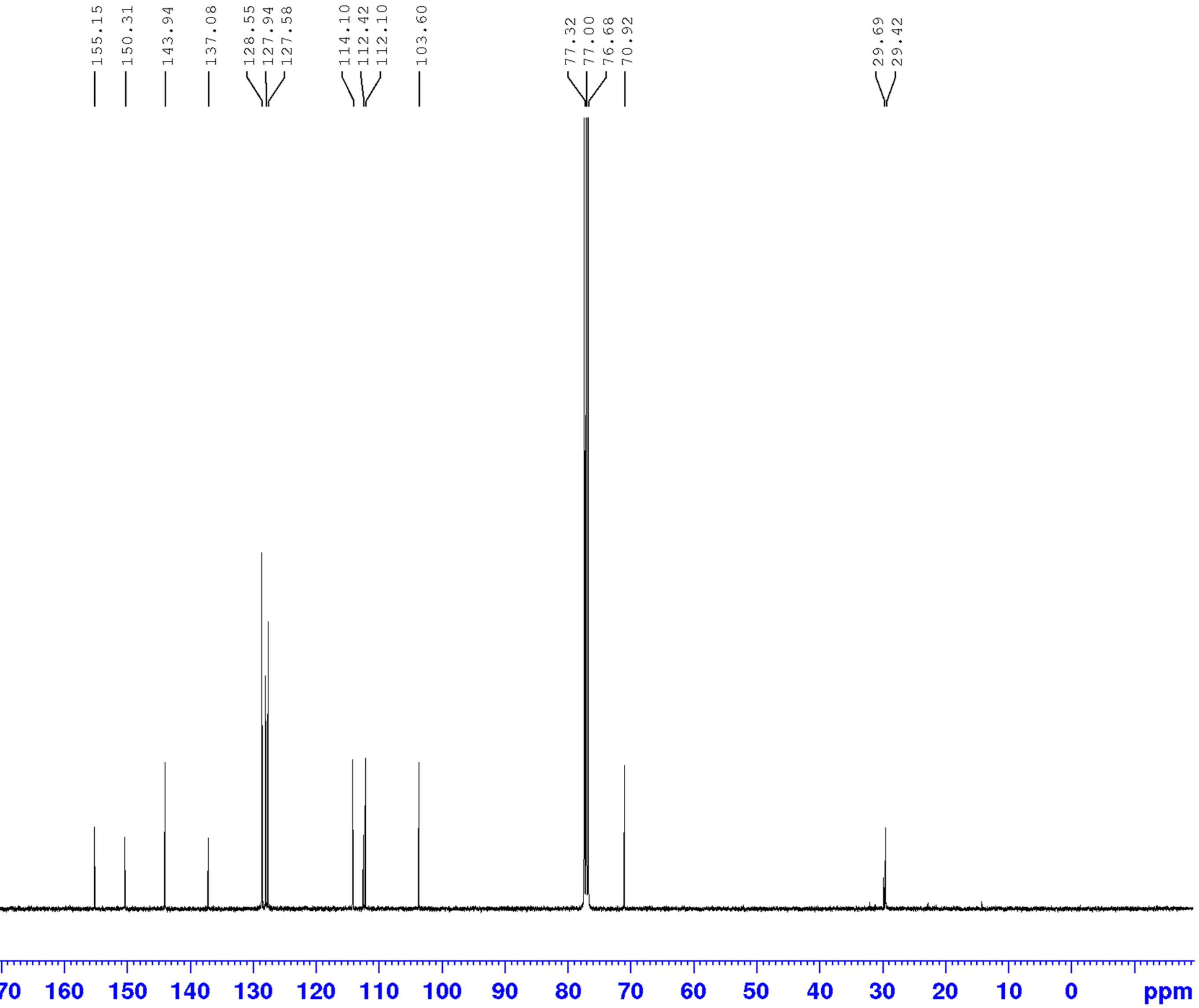

H-NMR (400 MHz, $CDCl_3$) of **2-(6-Phenoxybenzofuran-3-yl)acetic acid, 25**

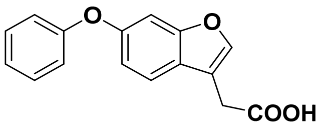

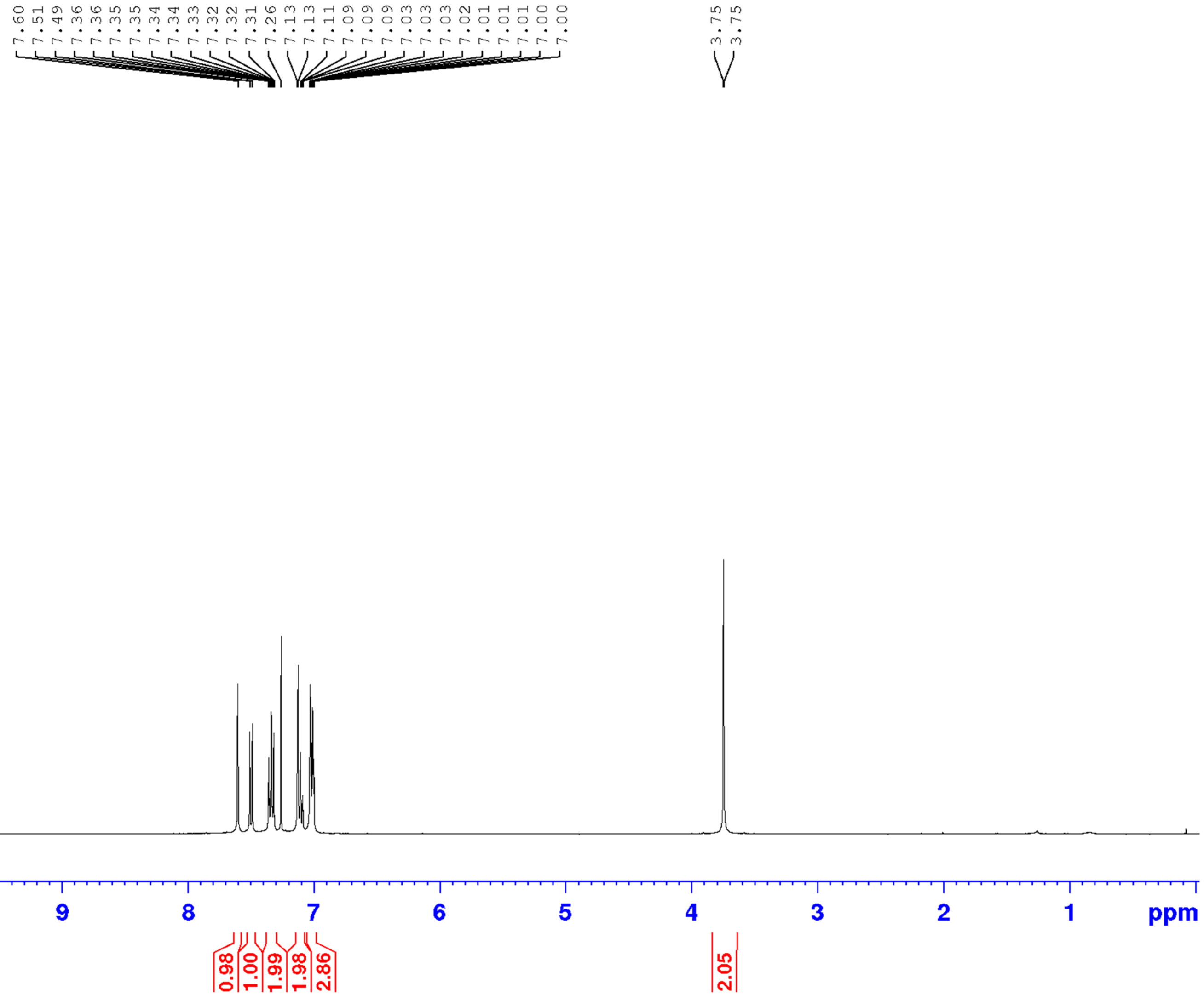

^{13}C-NMR (100 MHz, $CDCl_3$) of **2-(6-Phenoxybenzofuran-3-yl)acetic acid, 25**

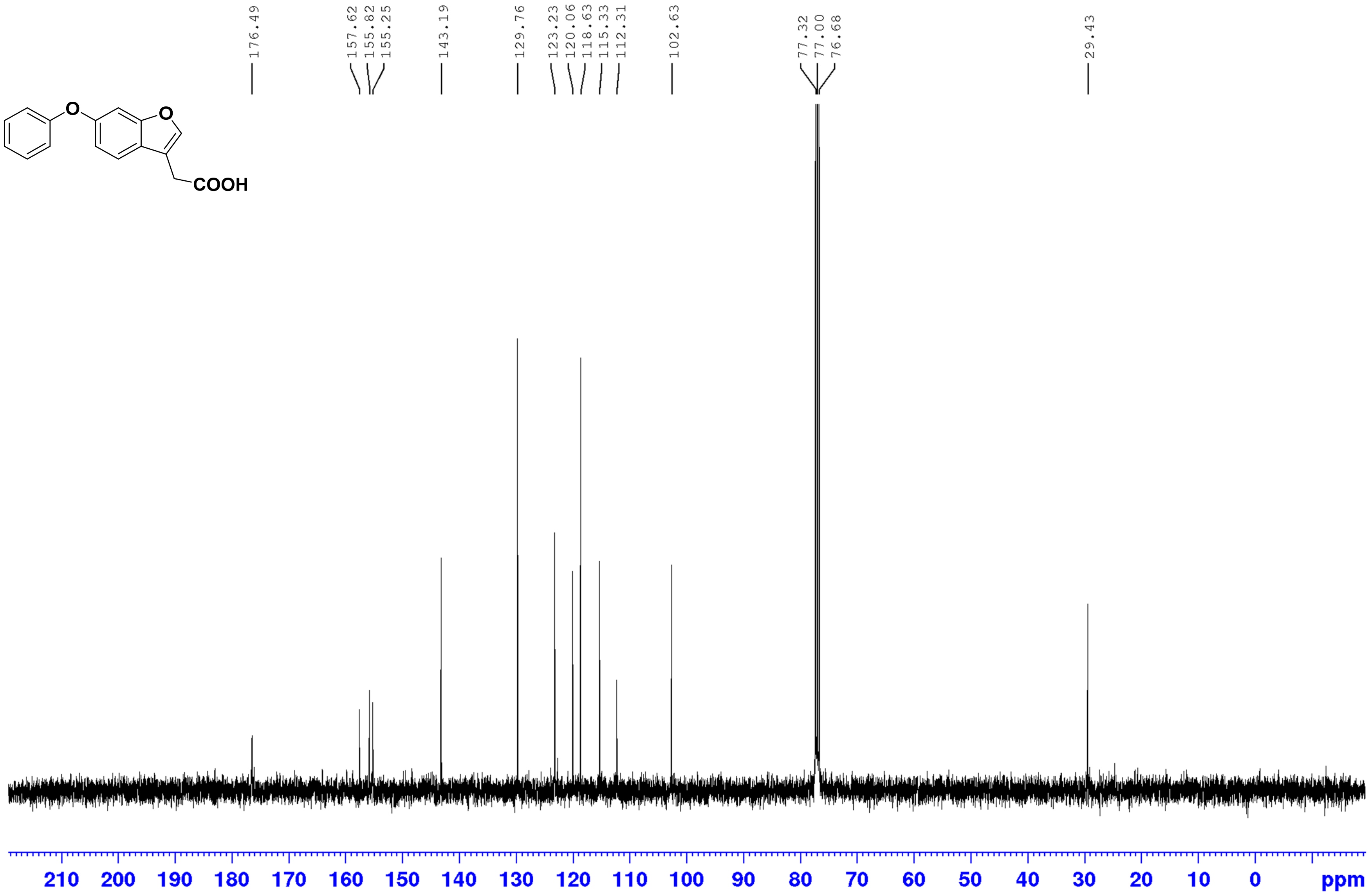

^{1}H-NMR (400 MHz, $CDCl_3$) of **2-(6-(3-Methoxyphenoxy)benzofuran-3-yl)acetic acid, 26**

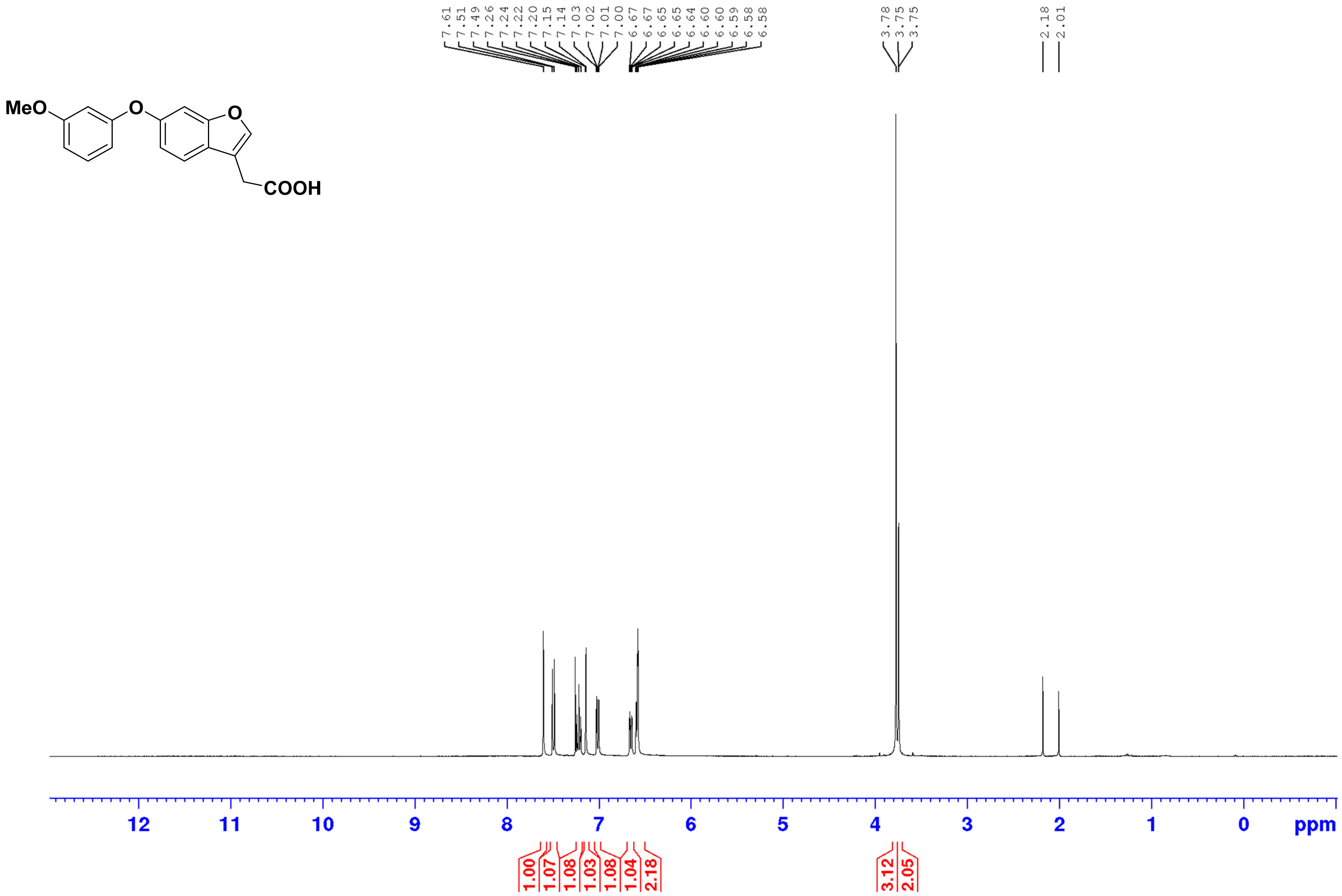

^{13}C-NMR (100 MHz, $CDCl_3$) of **2-(6-(3-Methoxyphenoxy)benzofuran-3-yl)acetic acid, 26**

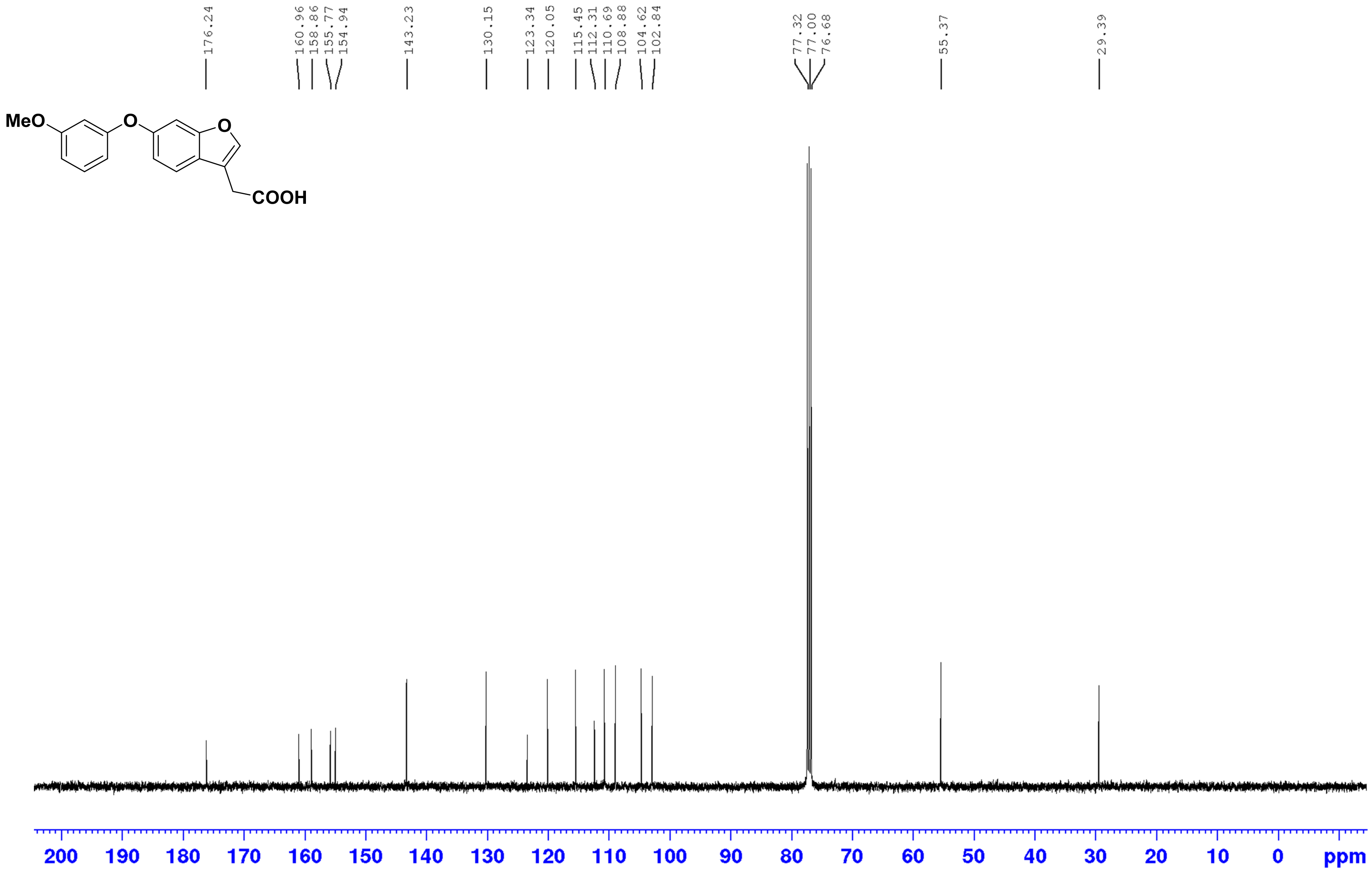

^{1}H-NMR (400 MHz, $CDCl_3$) of **Methyl 2-(6-benzylbenzofuran-3-yl)acetate, 27**

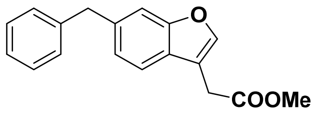

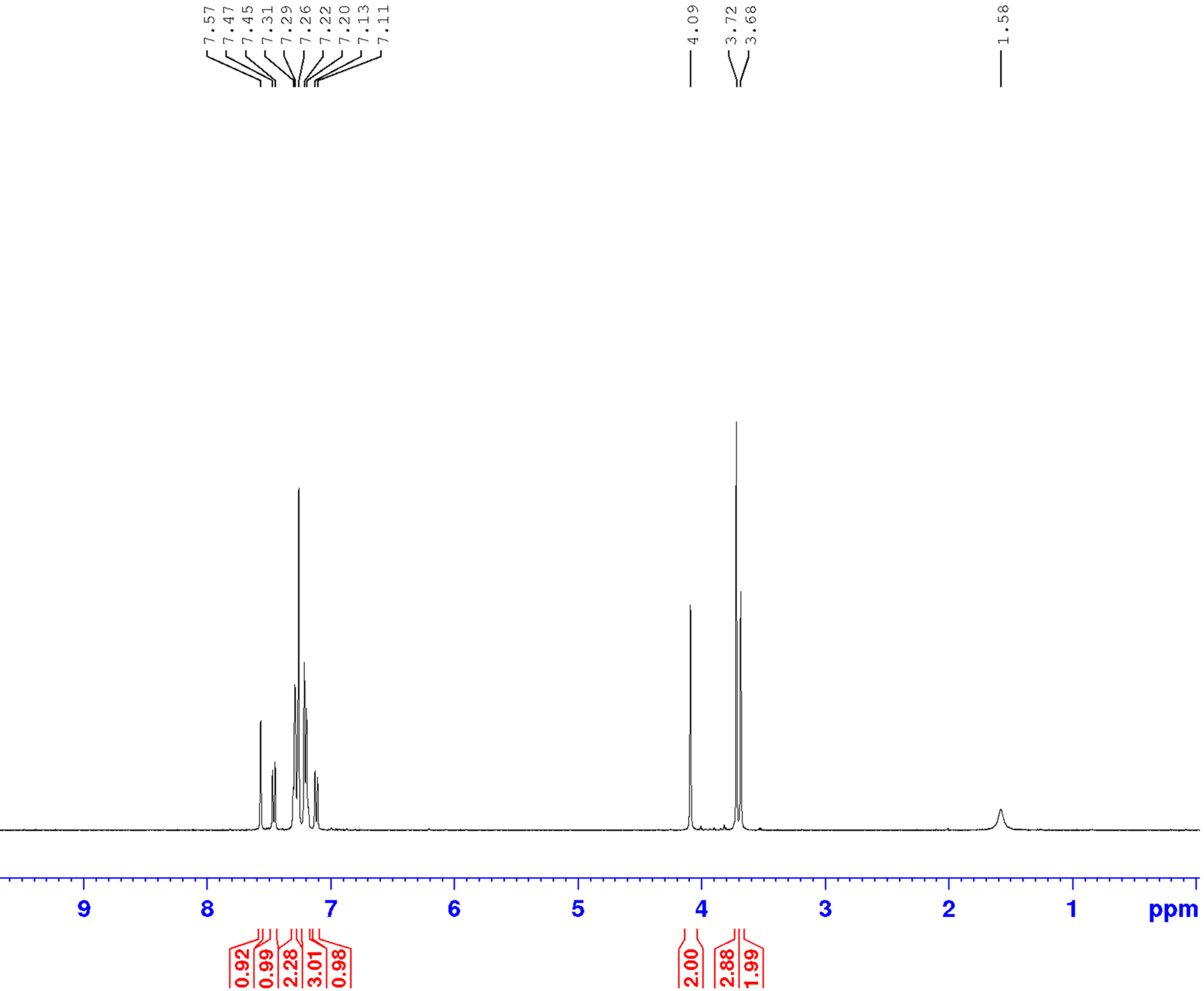

^{13}C-NMR (100 MHz, $CDCl_3$) of **Methyl 2-(6-benzylbenzofuran-3-yl)acetate, 27**

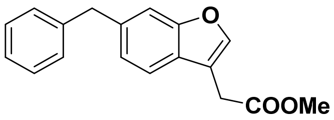

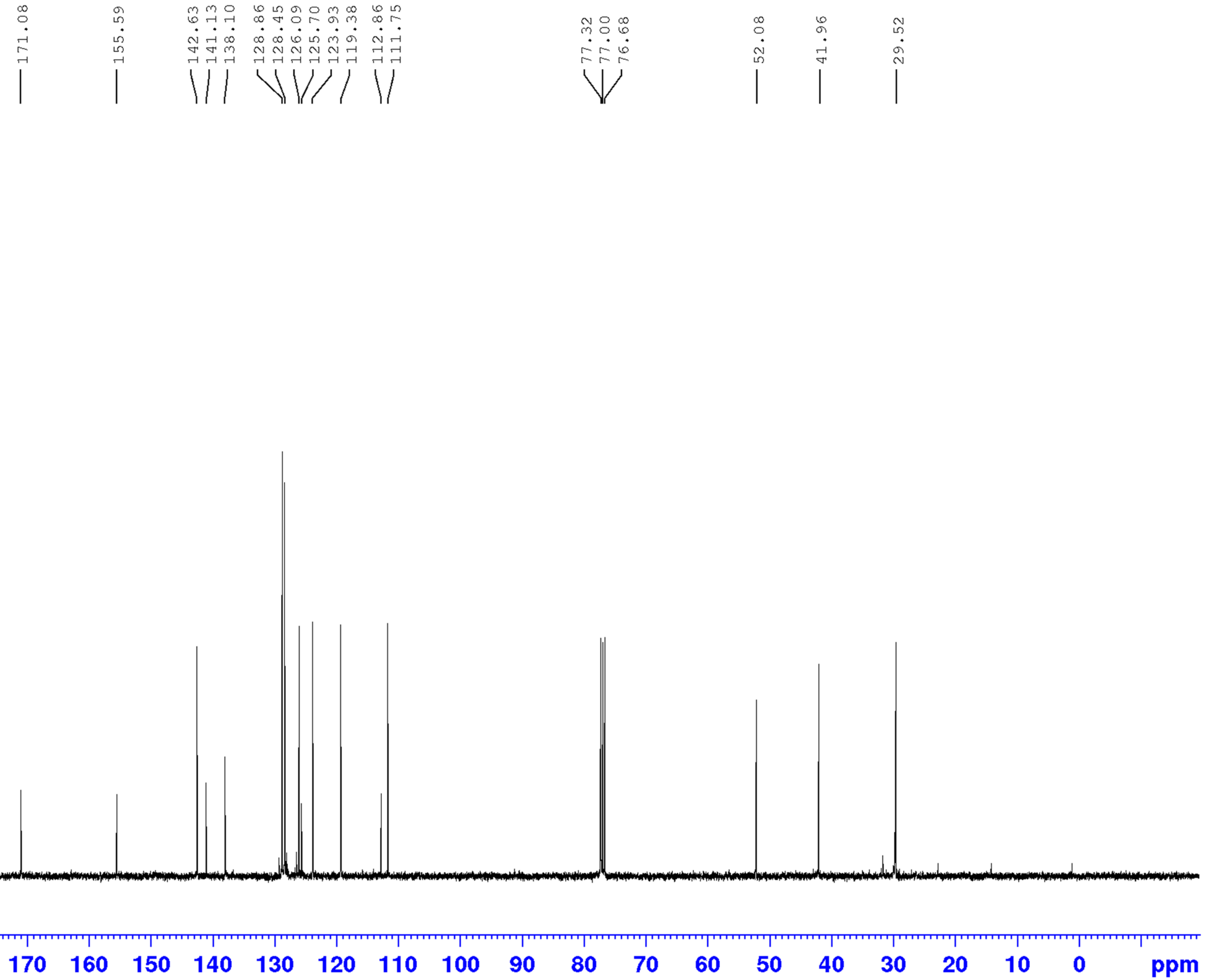

^{1}H-NMR (400 MHz, $CDCl_3$) of **2-(6-Benzylbenzofuran-3-yl)acetic acid, 28**

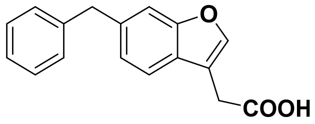

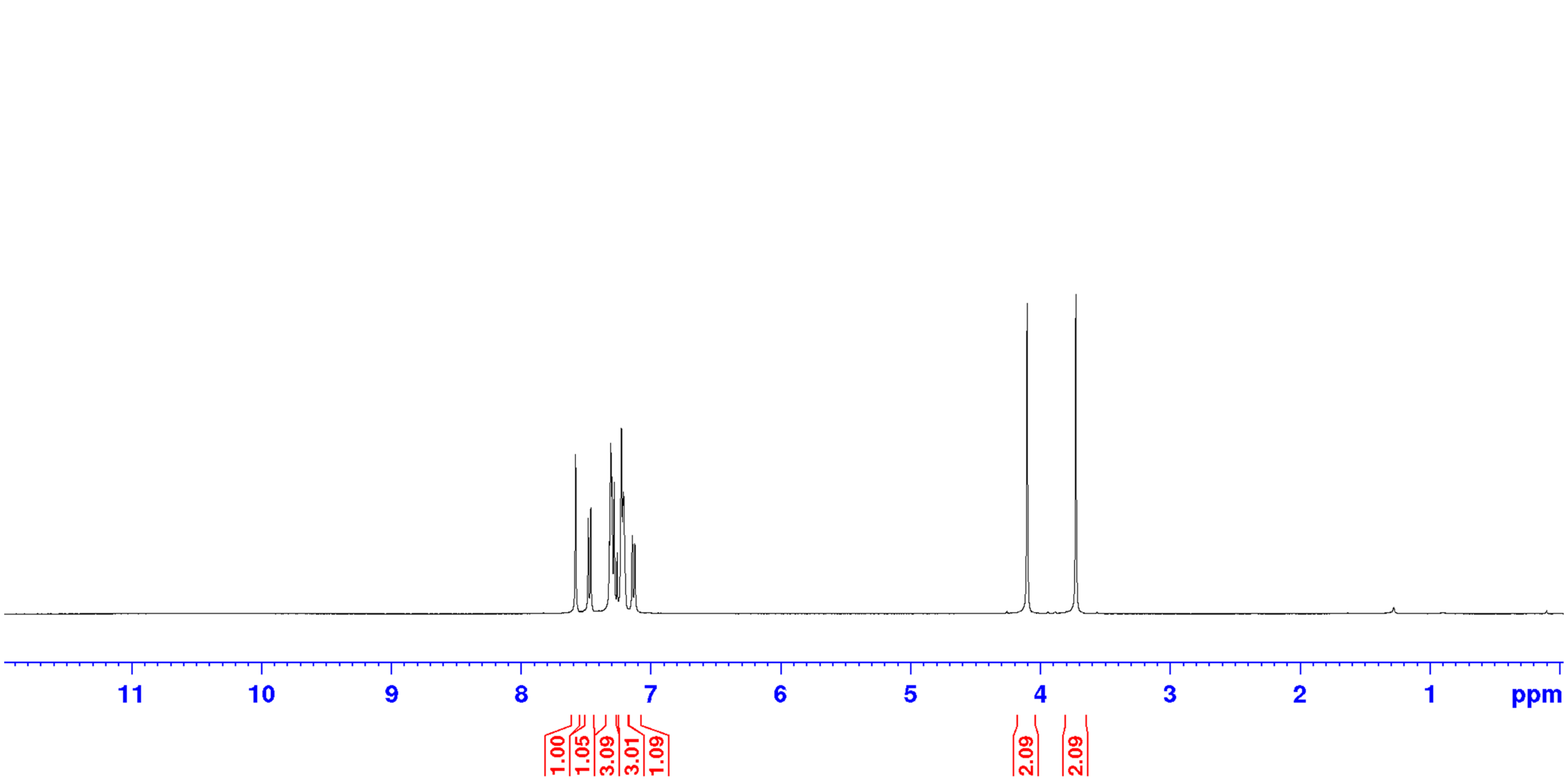

^{13}C-NMR (100 MHz, $CDCl_3$) of **2-(6-Benzylbenzofuran-3-yl)acetic acid, 28**

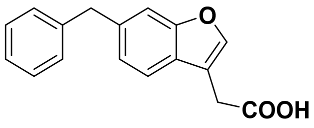

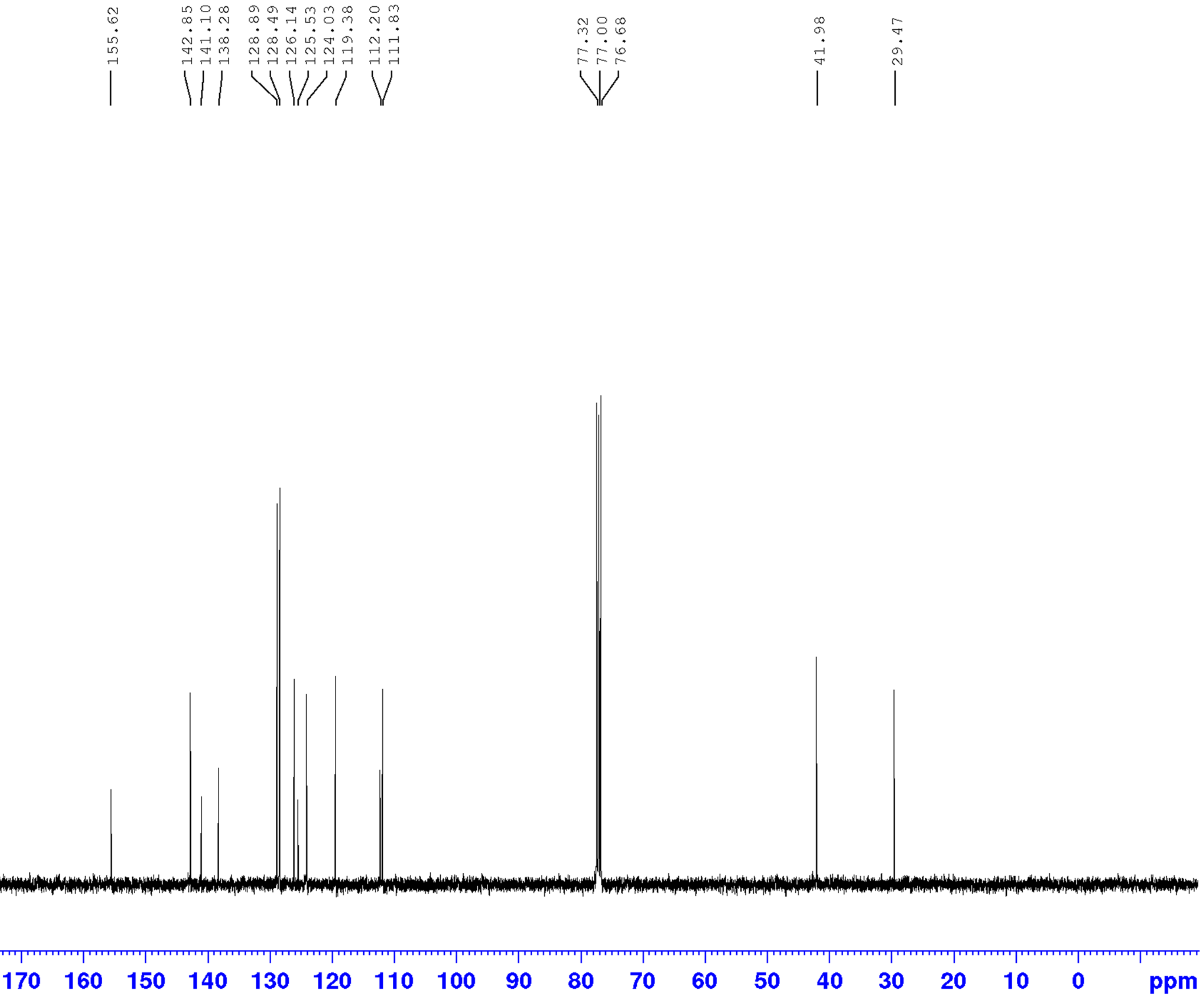

^{1}H-NMR (400 MHz, $CDCl_3$) of **2-(6-Bromobenzofuran-3-yl)acetamide, 29**

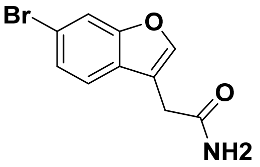

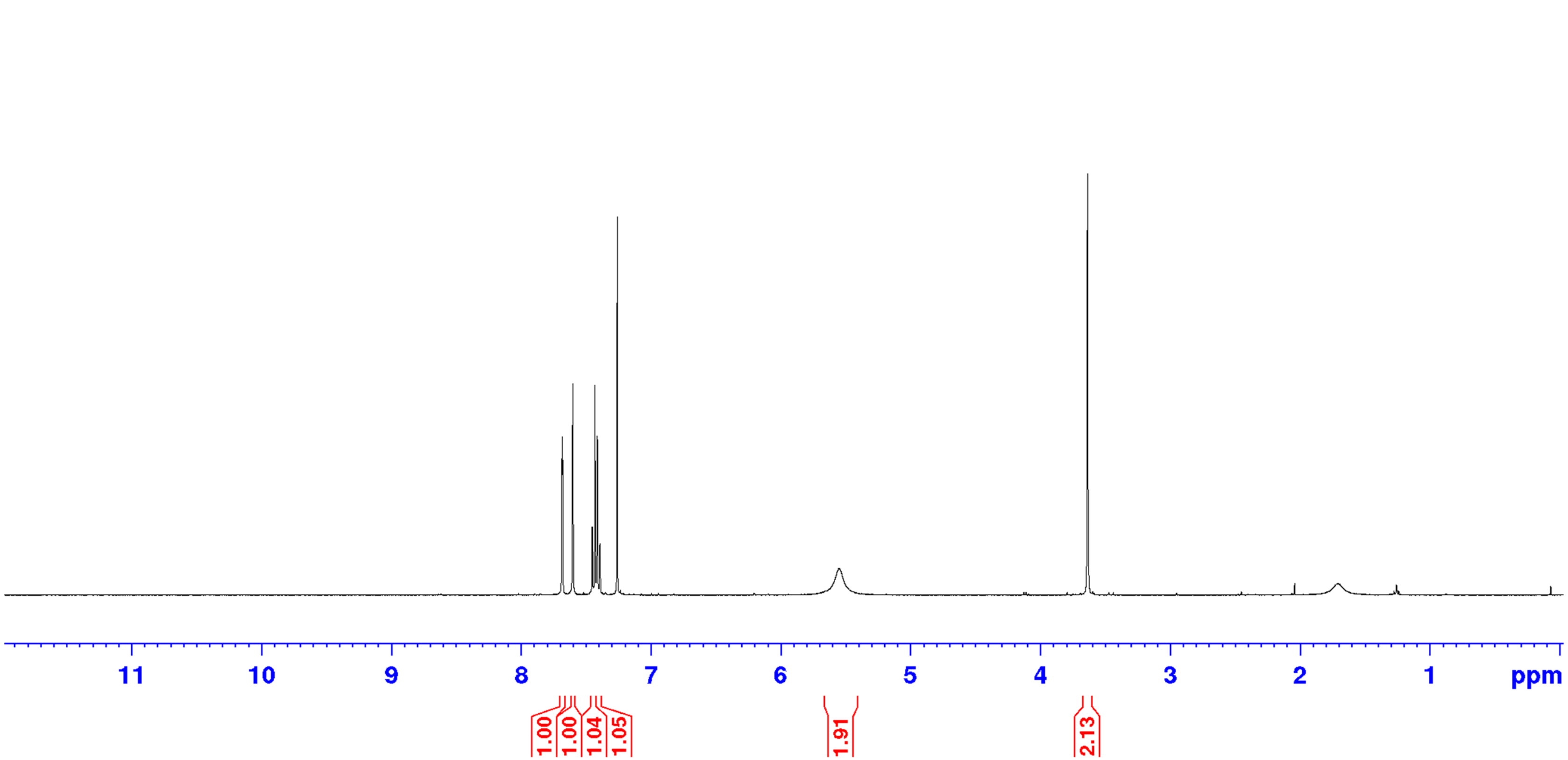

^{13}C-NMR (100 MHz, DMSO-d_6) of **2-(6-Bromobenzofuran-3-yl)acetamide, 29**

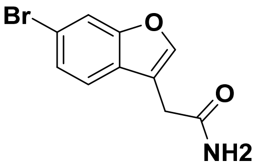

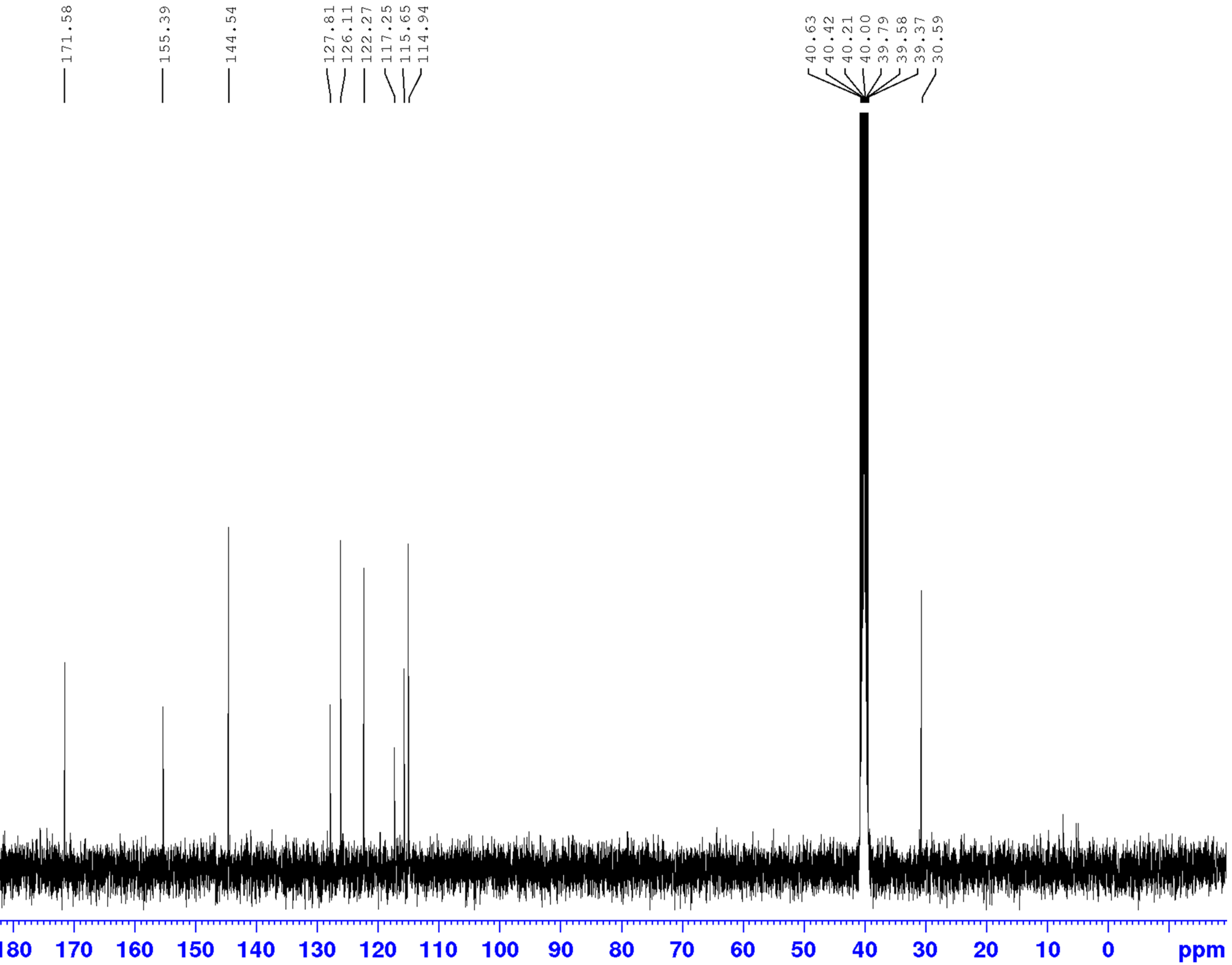

^{1}H-NMR (400 MHz, $CDCl_3$) of **2-(6-Bromobenzofuran-3-yl)acetonitrile, 30**

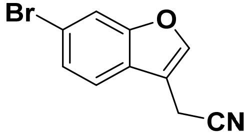

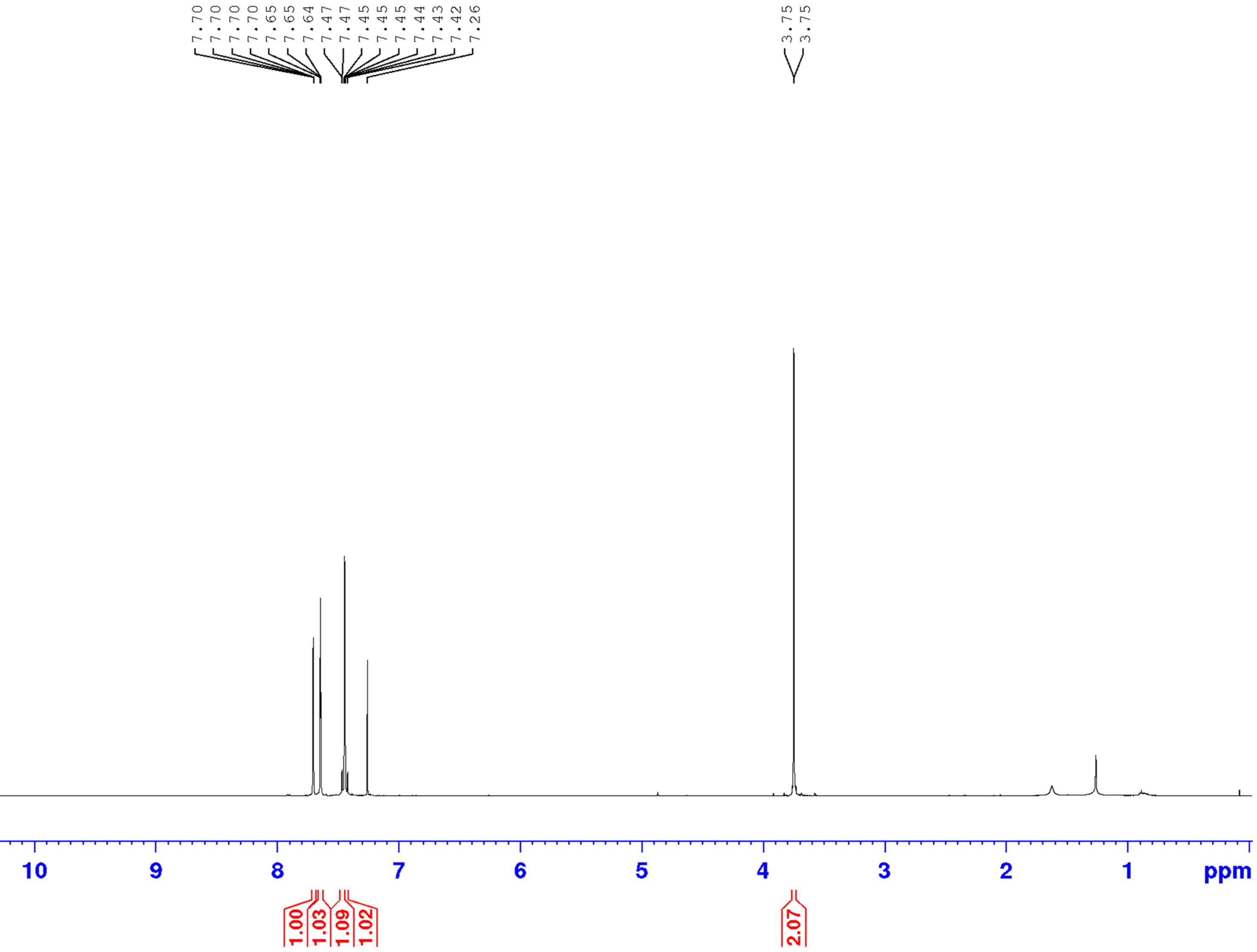

^{13}C-NMR (100 MHz, $CDCl_3$) of **2-(6-Bromobenzofuran-3-yl)acetonitrile, 30**

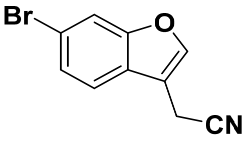

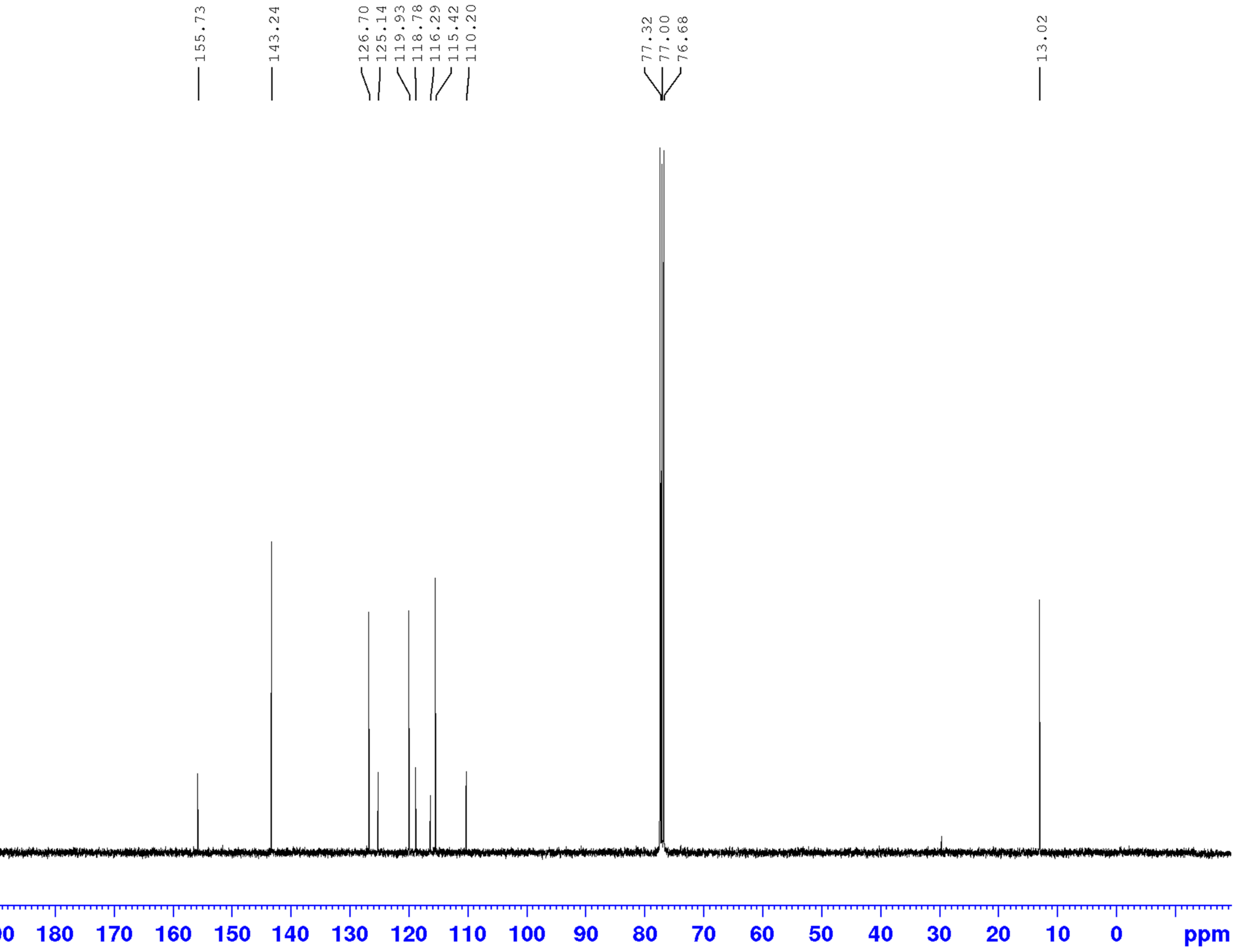

^{1}H-NMR (400 MHz, DMSO-d_6) of **5-((6-Bromobenzofuran-3-yl)methyl)-1*H*-tetrazole, 31**

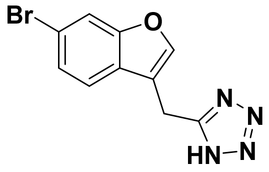

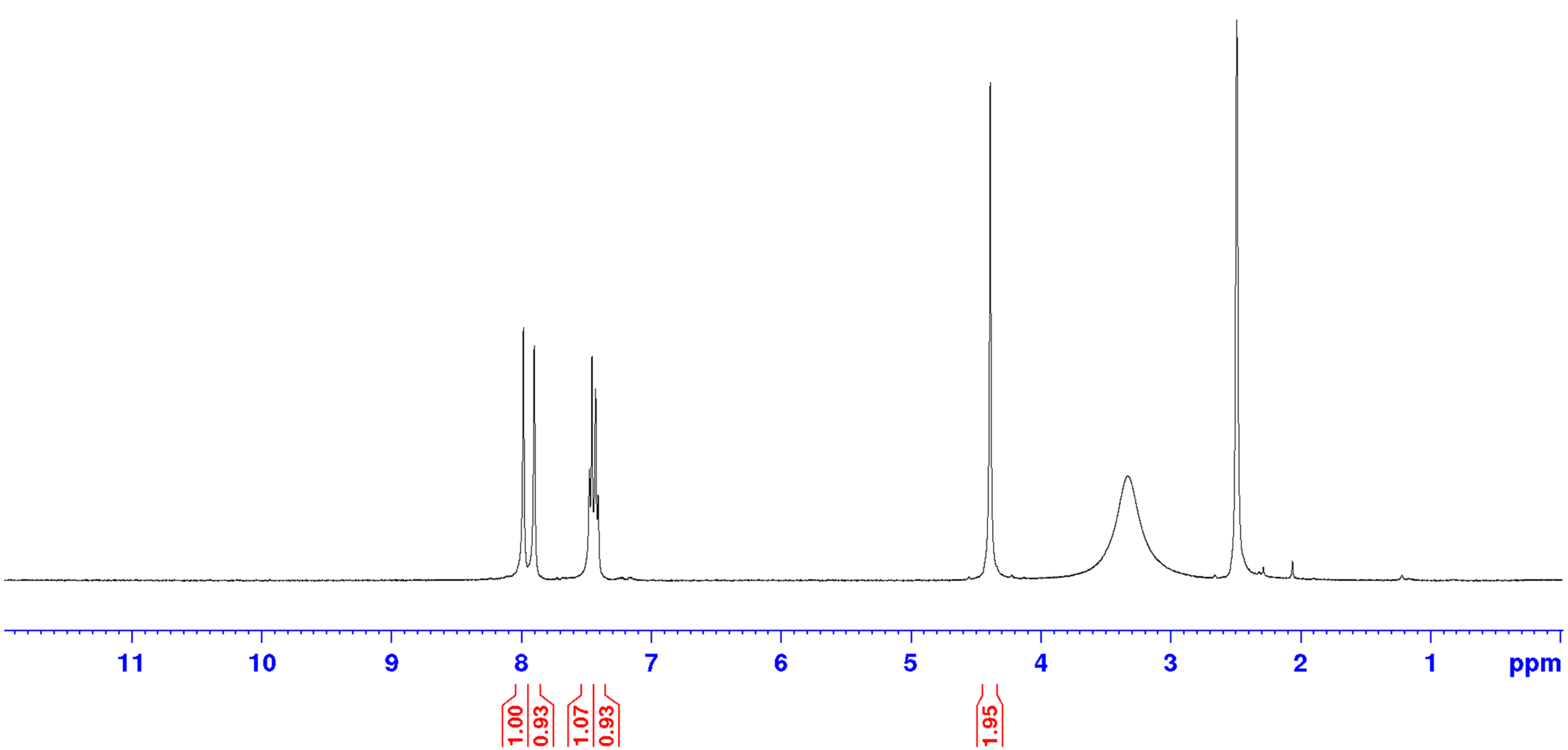

^{13}C-NMR (100 MHz, DMSO-d_6) of **5-((6-Bromobenzofuran-3-yl)methyl)-1*H*-tetrazole, 31**

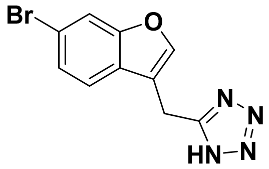

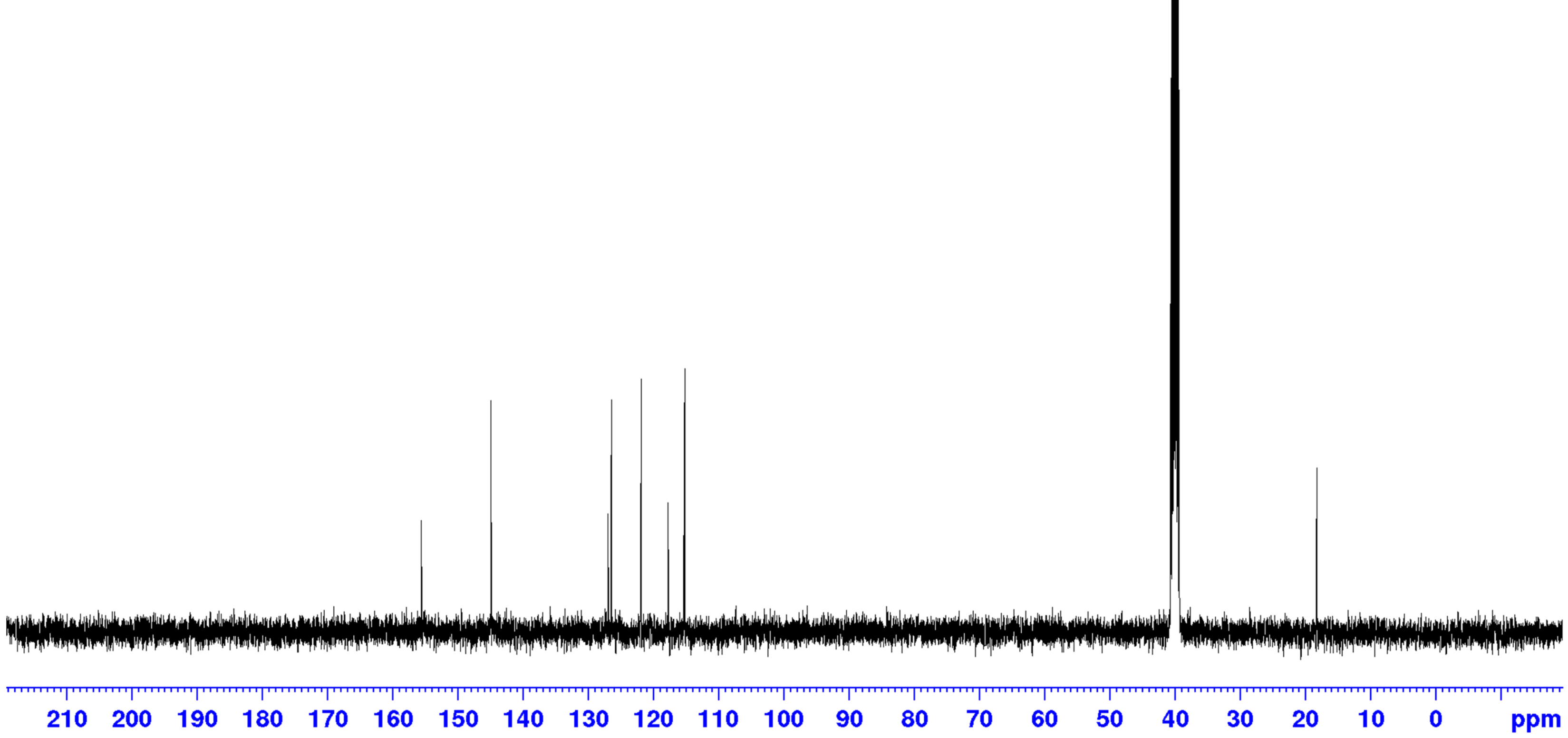

^{1}H-NMR (400 MHz, $CDCl_3$) of **Dimethyl 2-(1-(methoxycarbonyl)-6-phenyl-1*H*-indol-3-yl)malonate, 35**

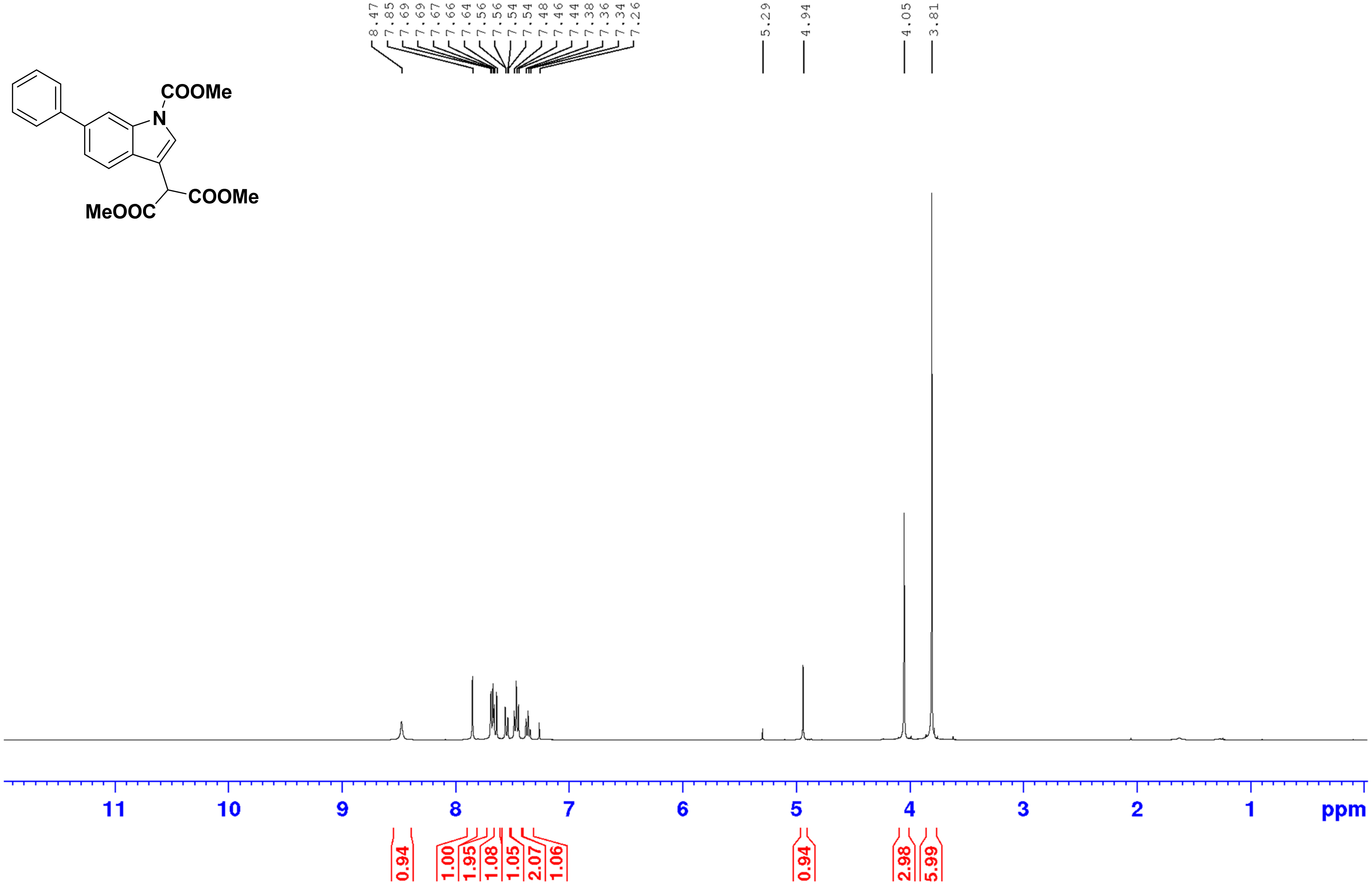

^{13}C-NMR (100 MHz, $CDCl_3$) of **Dimethyl 2-(1-(methoxycarbonyl)-6-phenyl-1*H*-indol-3-yl)malonate, 35**

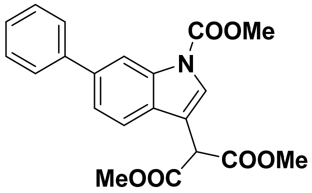

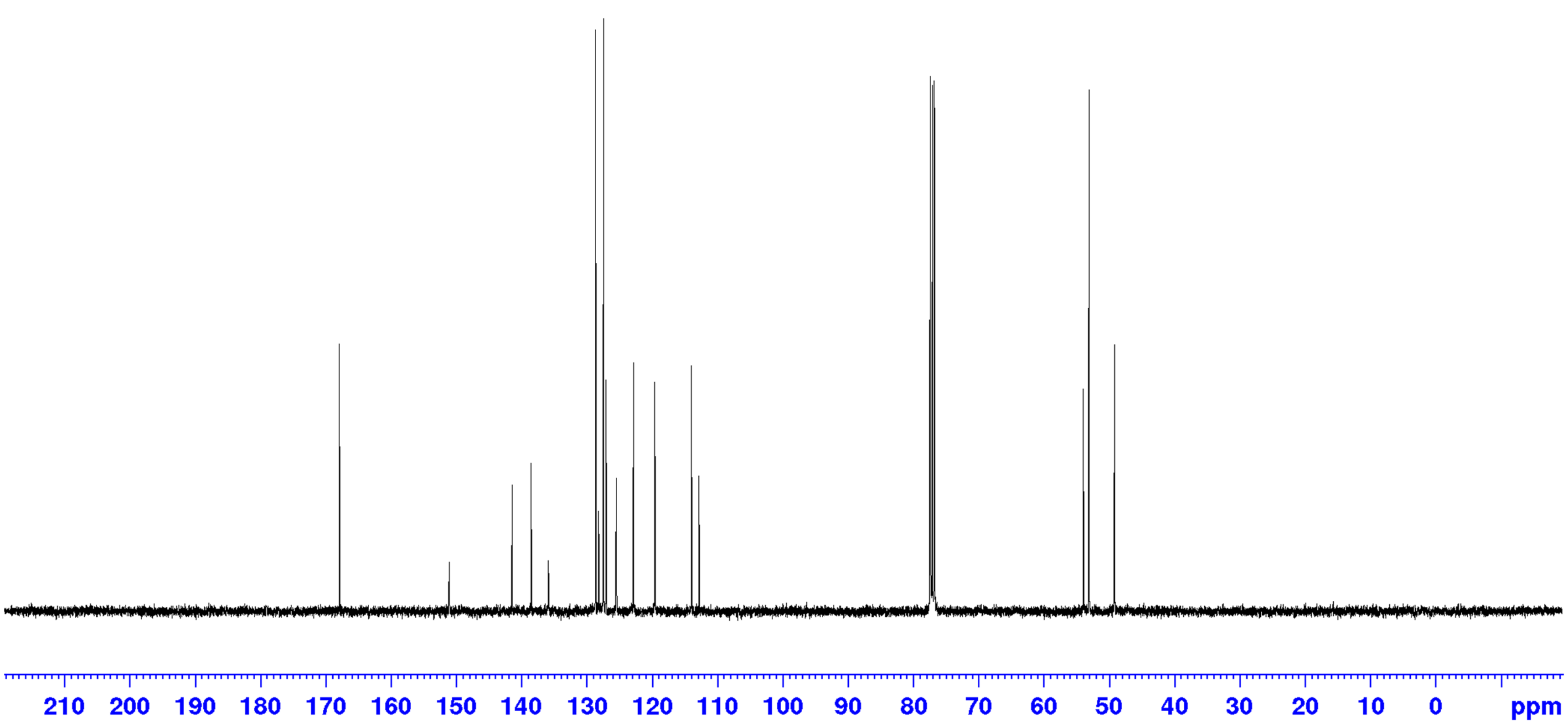

^{1}H-NMR (500 MHz, acetone-d_6) of **2-(6-Phenyl-1*H*-3-yl)acetic acid, 36**

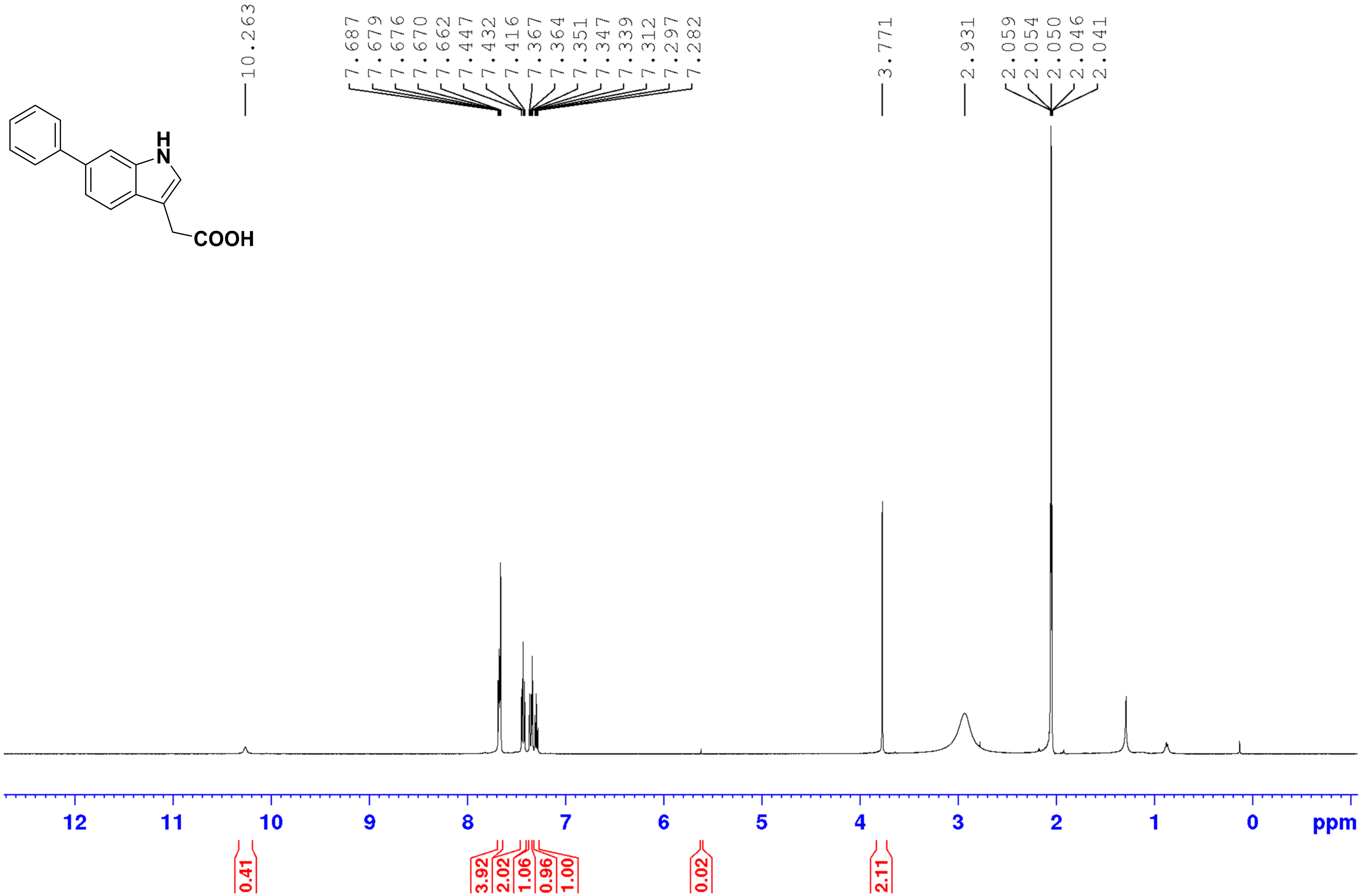

^{13}C-NMR (100 MHz, acetone-d_6) of **2-(6-Phenyl-1*H*-3-yl)acetic acid, 36**

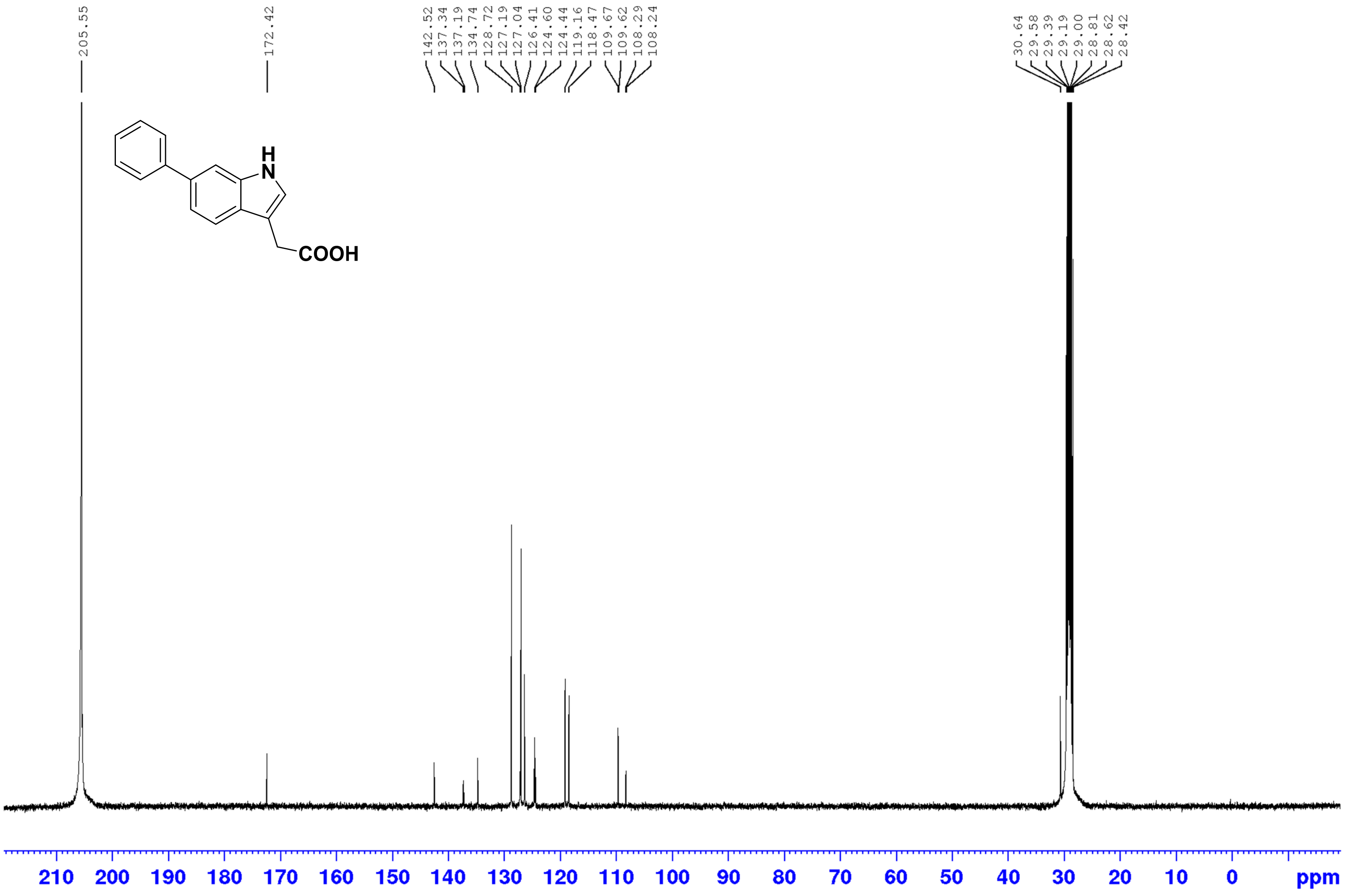

^{1}H-NMR (400 MHz, $CDCl_3$) of **Methyl 2-bromo-2-(6-bromobenzofuran-3-yl)acetate, 37**

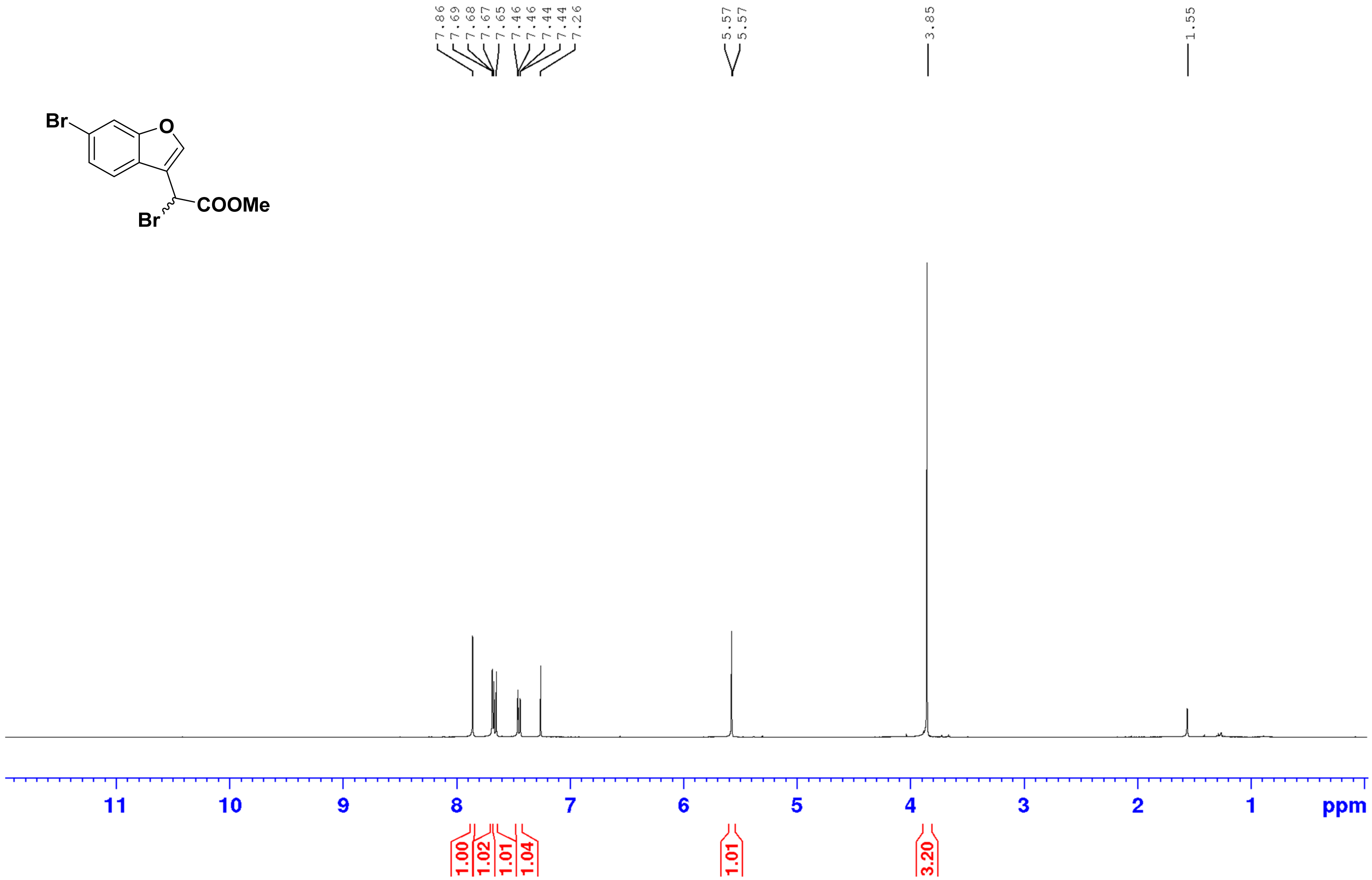

^{13}C-NMR (100 MHz, $CDCl_3$) of **Methyl 2-bromo-2-(6-bromobenzofuran-3-yl)acetate, 37**

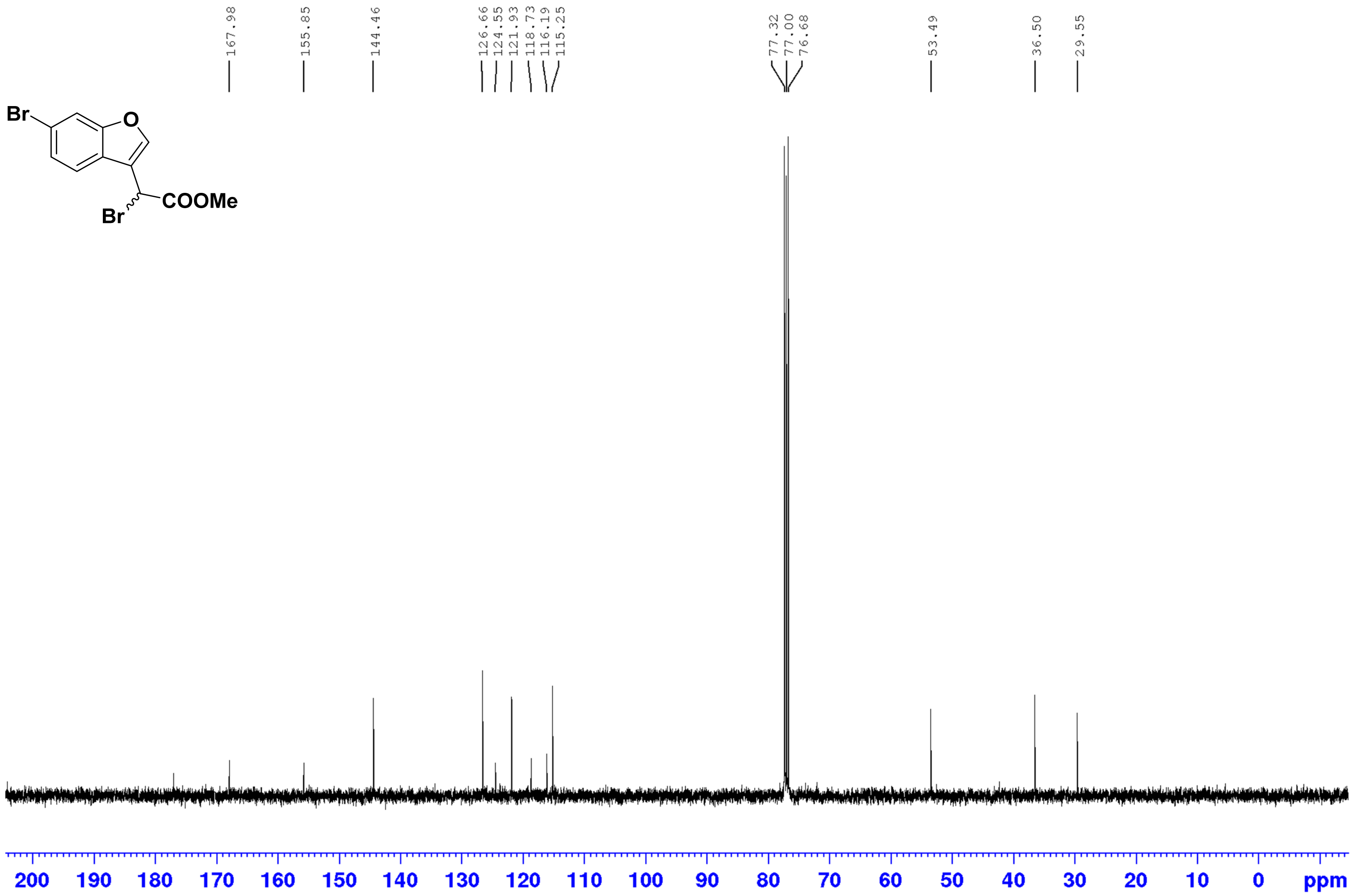

^{1}H-NMR (400 MHz, $CDCl_3$) of **Methyl-2-azido-2-(6-bromobenzofuran-3-yl)acetate, 38**

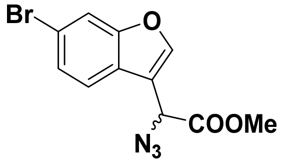

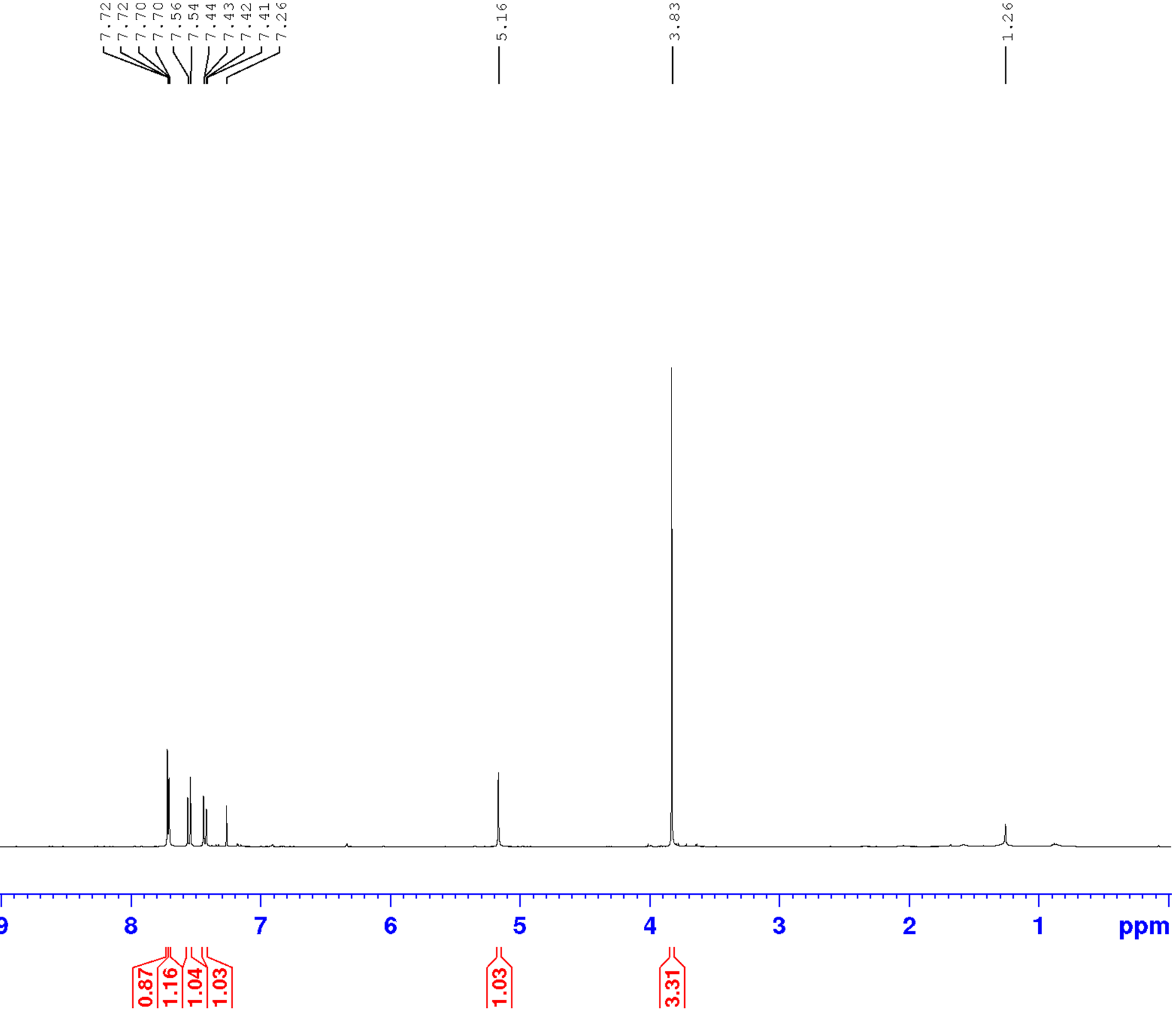

^{13}C-NMR (100 MHz, $CDCl_3$) of **Methyl-2-azido-2-(6-bromobenzofuran-3-yl)acetate, 38**

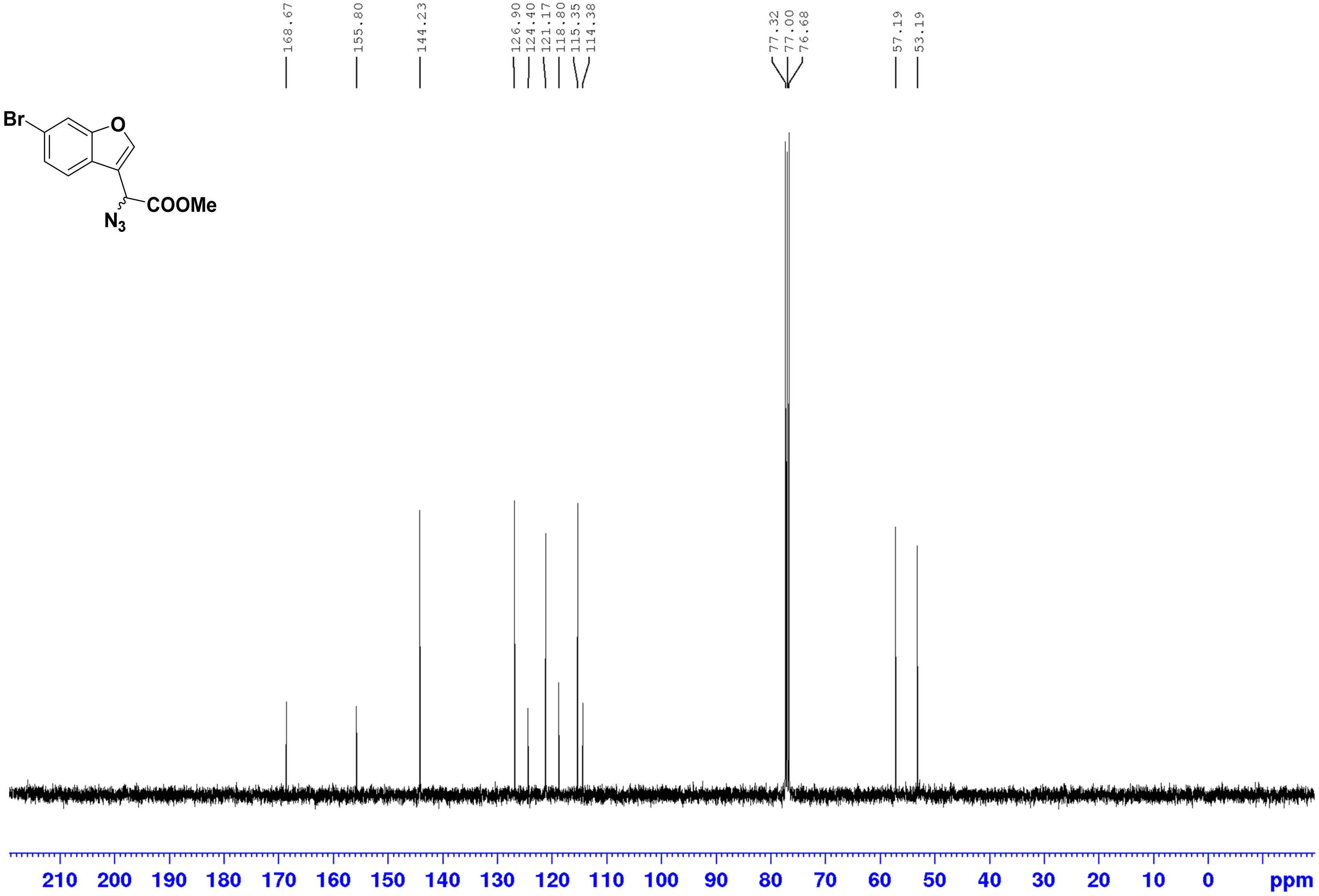

^{1}H-NMR (500 MHz, $CDCl_3$) of **Methyl-2-amino-2-(6-bromobenzofuran-3-yl)acetate, 39**

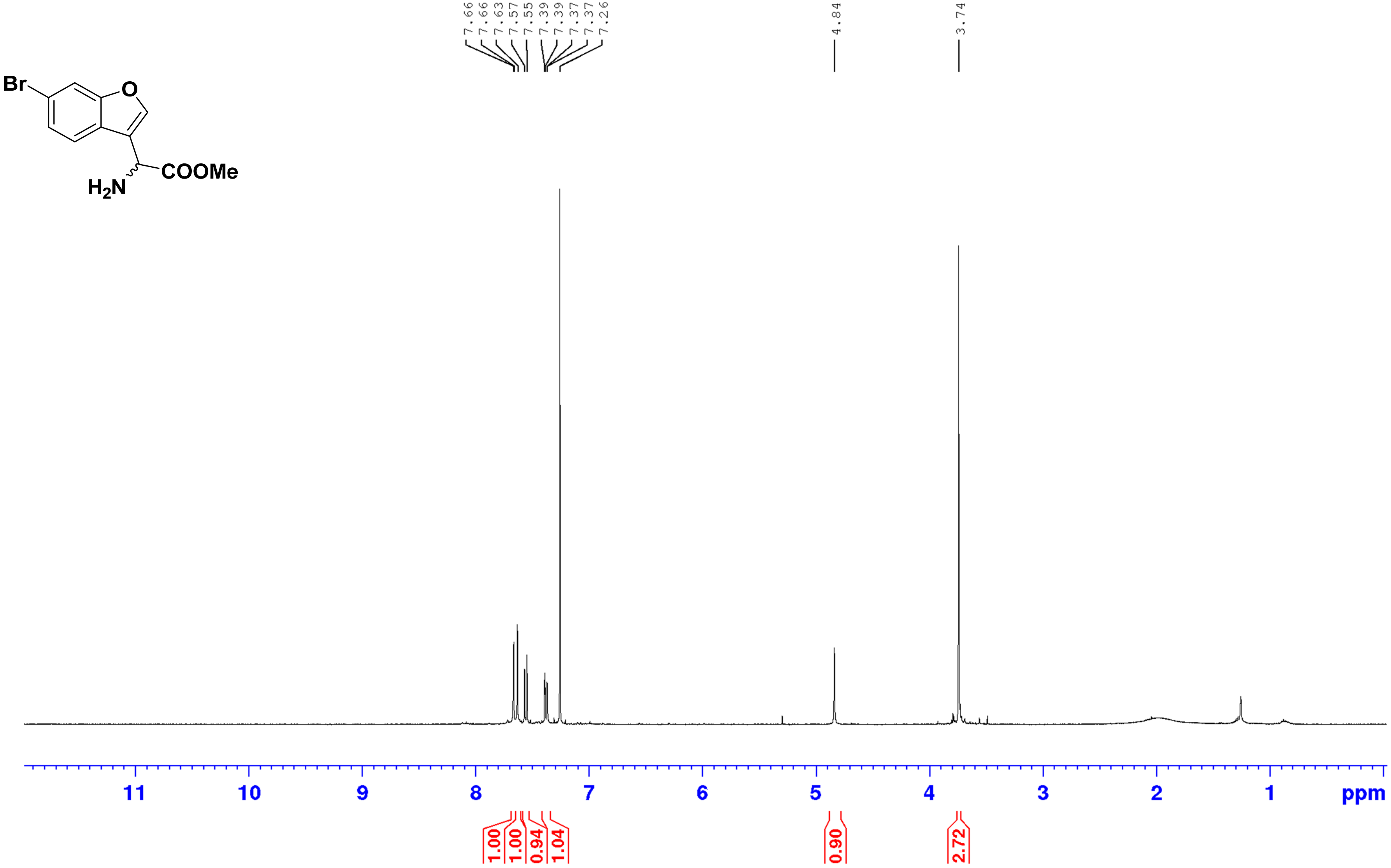

^{13}C-NMR (100 MHz, $CDCl_3$) of **Methyl-2-amino-2-(6-bromobenzofuran-3-yl)acetate, 39**

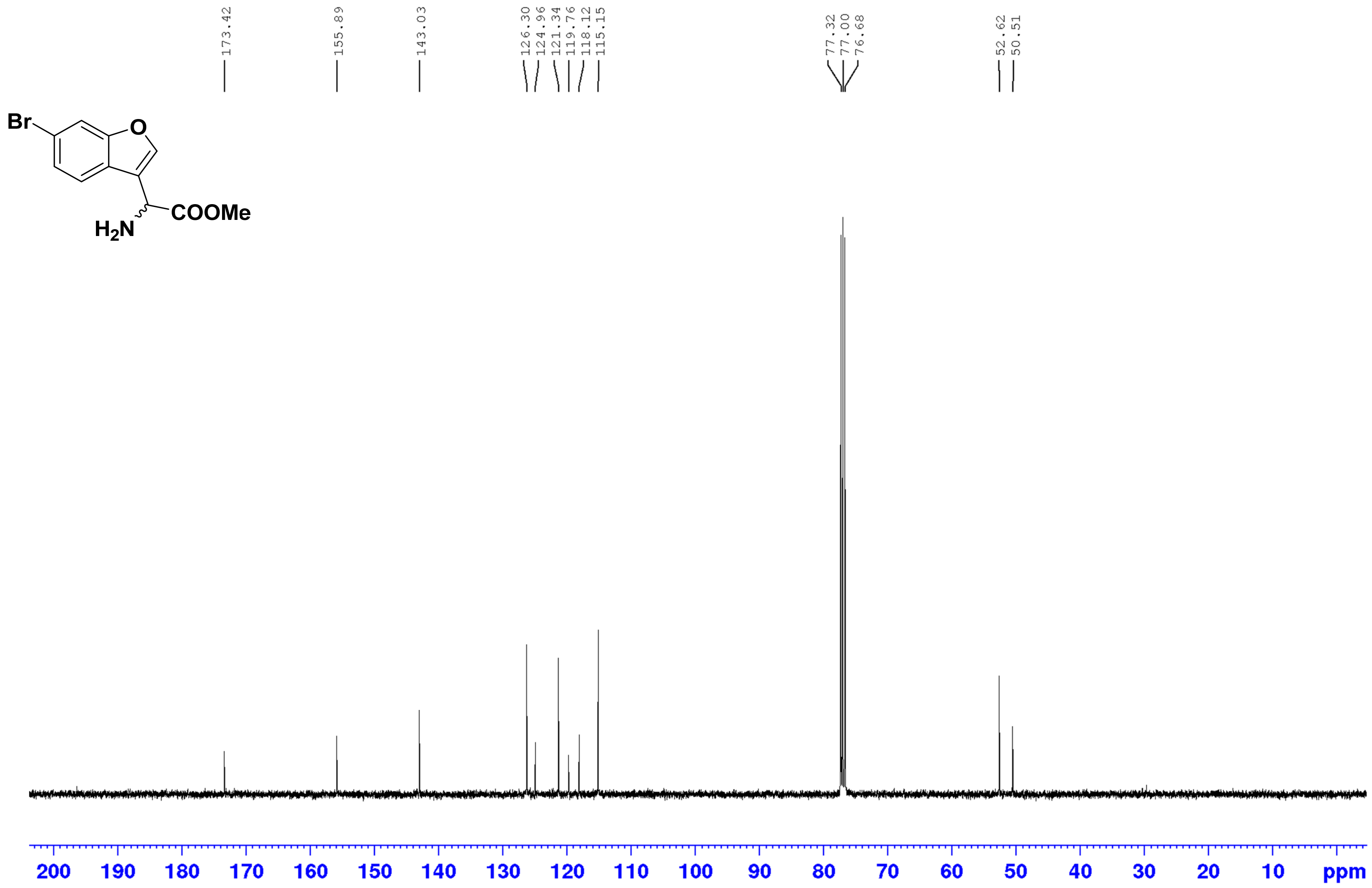

^{1}H-NMR (500 MHz, acetone-d_6) of **2-Acetamido-2-(6-bromobenzofuran-3-yl)acetic acid, 46**

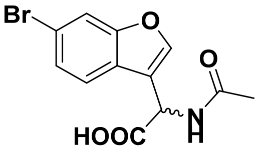

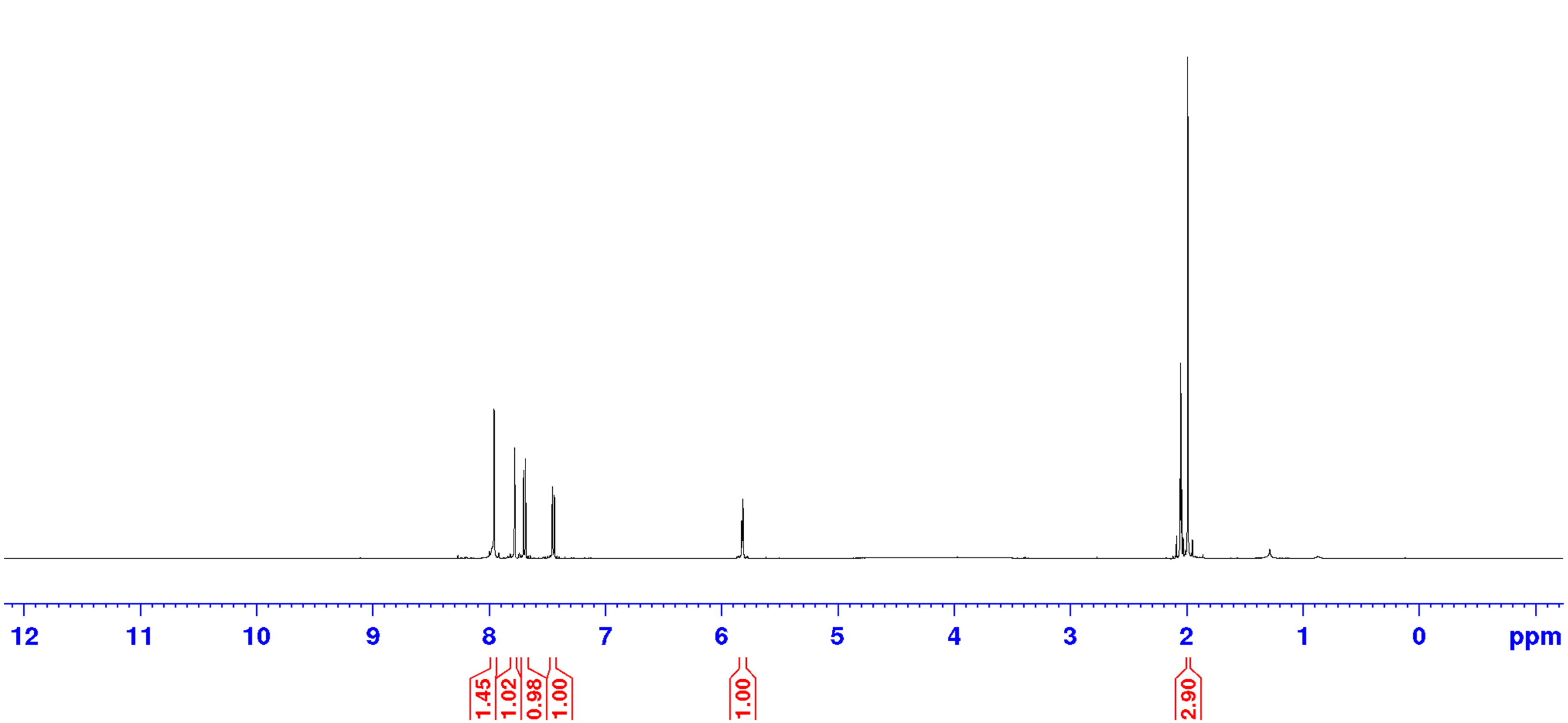

^{13}C-NMR (100 MHz, acetone-d_6) of **2-Acetamido-2-(6-bromobenzofuran-3-yl)acetic acid, 46**

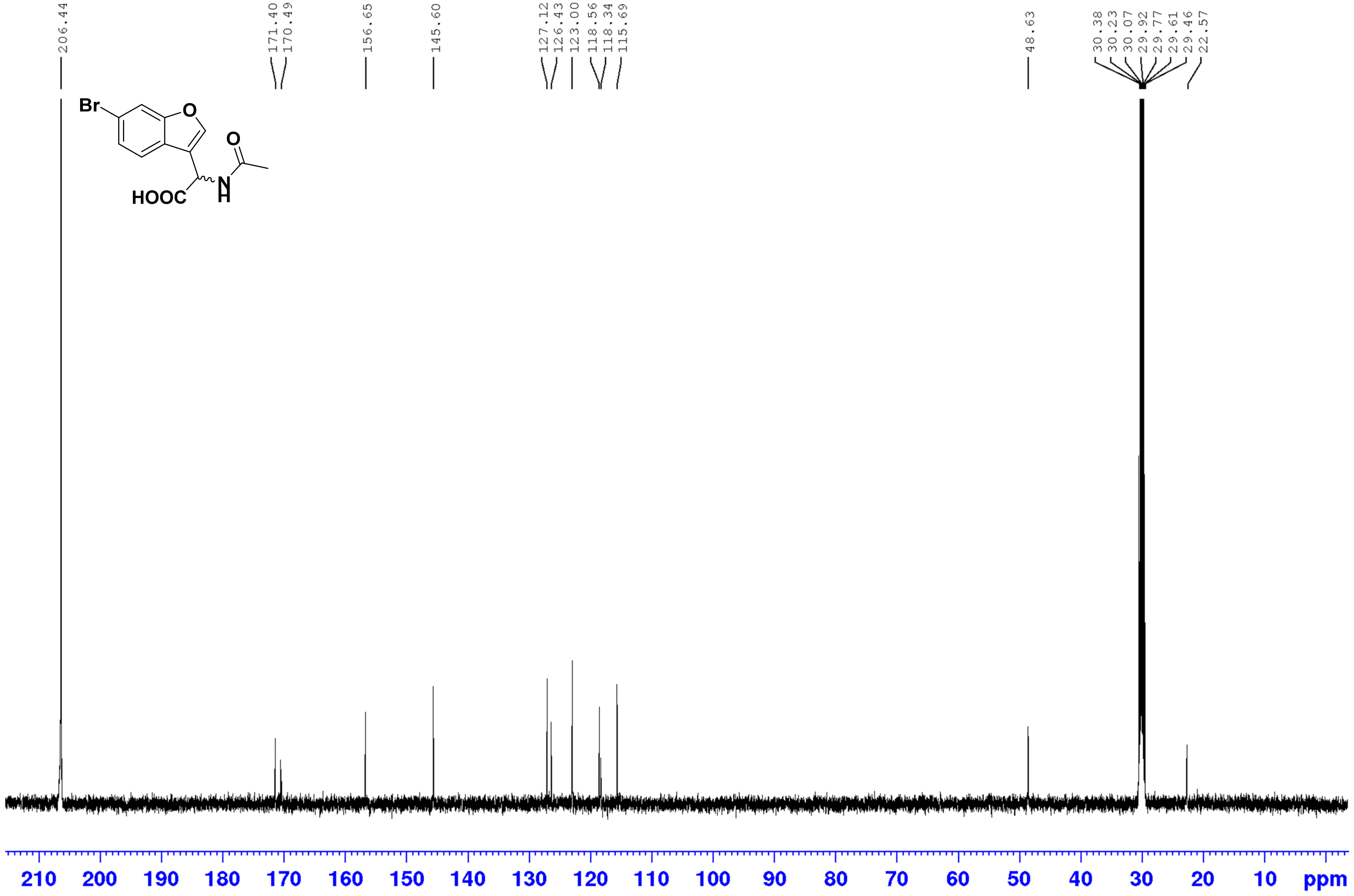

^{1}H-NMR (500 MHz, $CDCl_3$) of **2-Benzamido-2-(6-bromobenzofuran-3-yl)-2-acetic acid, 47**

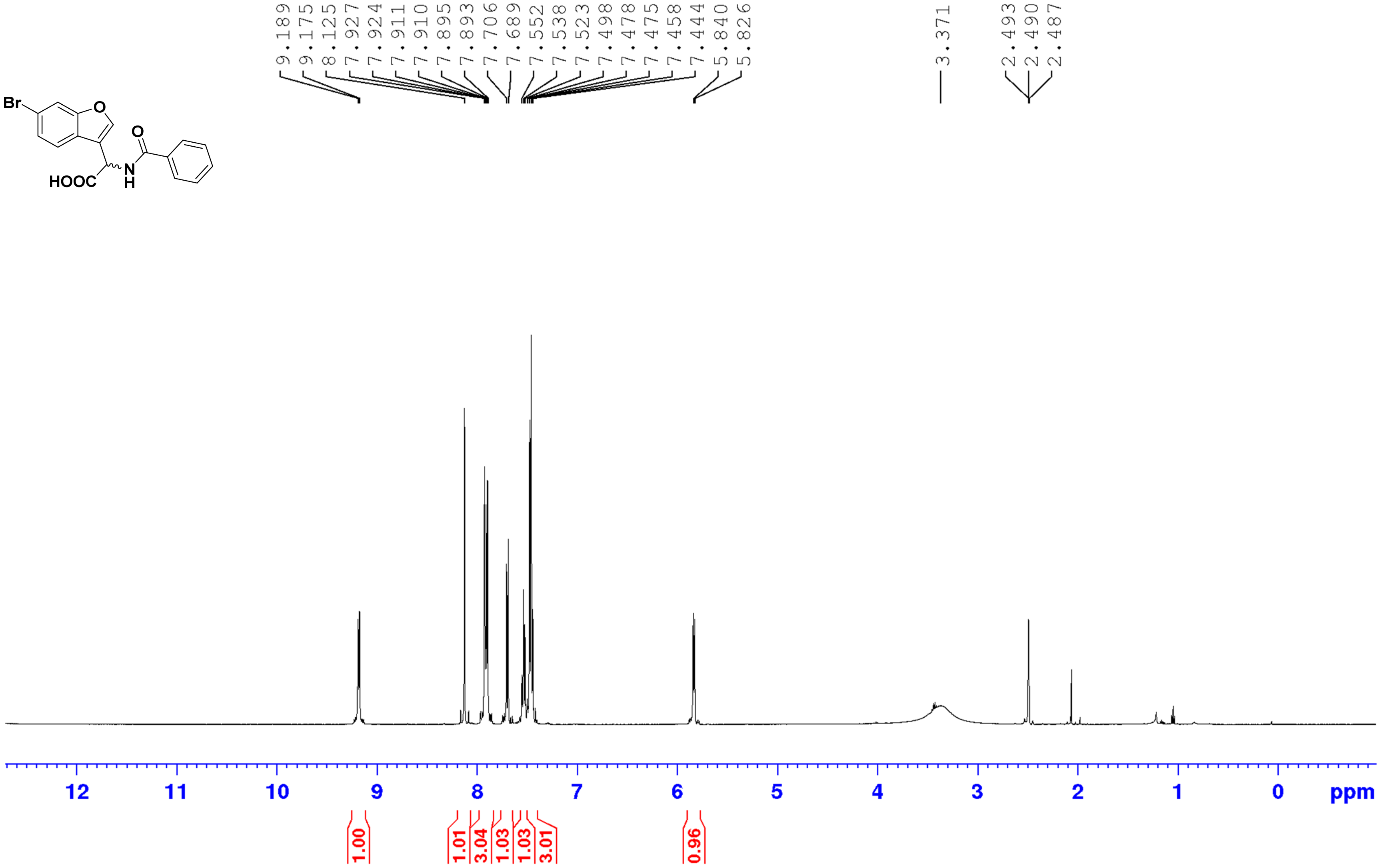

^{13}C-NMR (100 MHz, DMSO-d_6) of **2-Benzamido-2-(6-bromobenzofuran-3-yl)-2-acetic acid, 47**

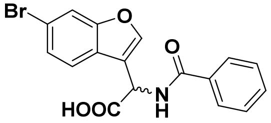

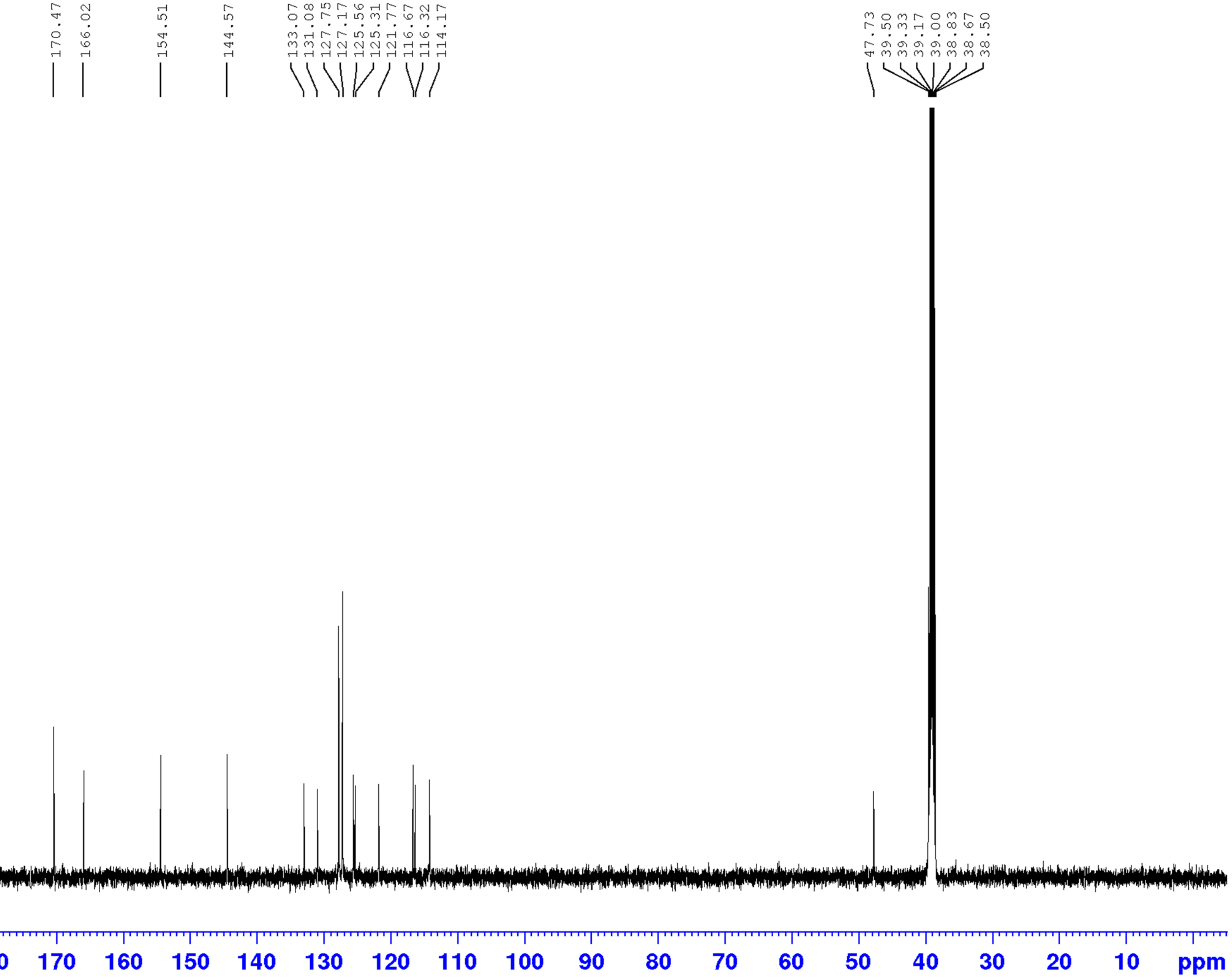

^{1}H-NMR (400 MHz, CD_3OD) of **2-(6-Bromobenzofuran-3-yl)-2-(2-(4-methoxyphenyl)acetamido)acetic acid, 48**

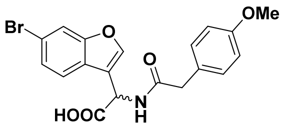

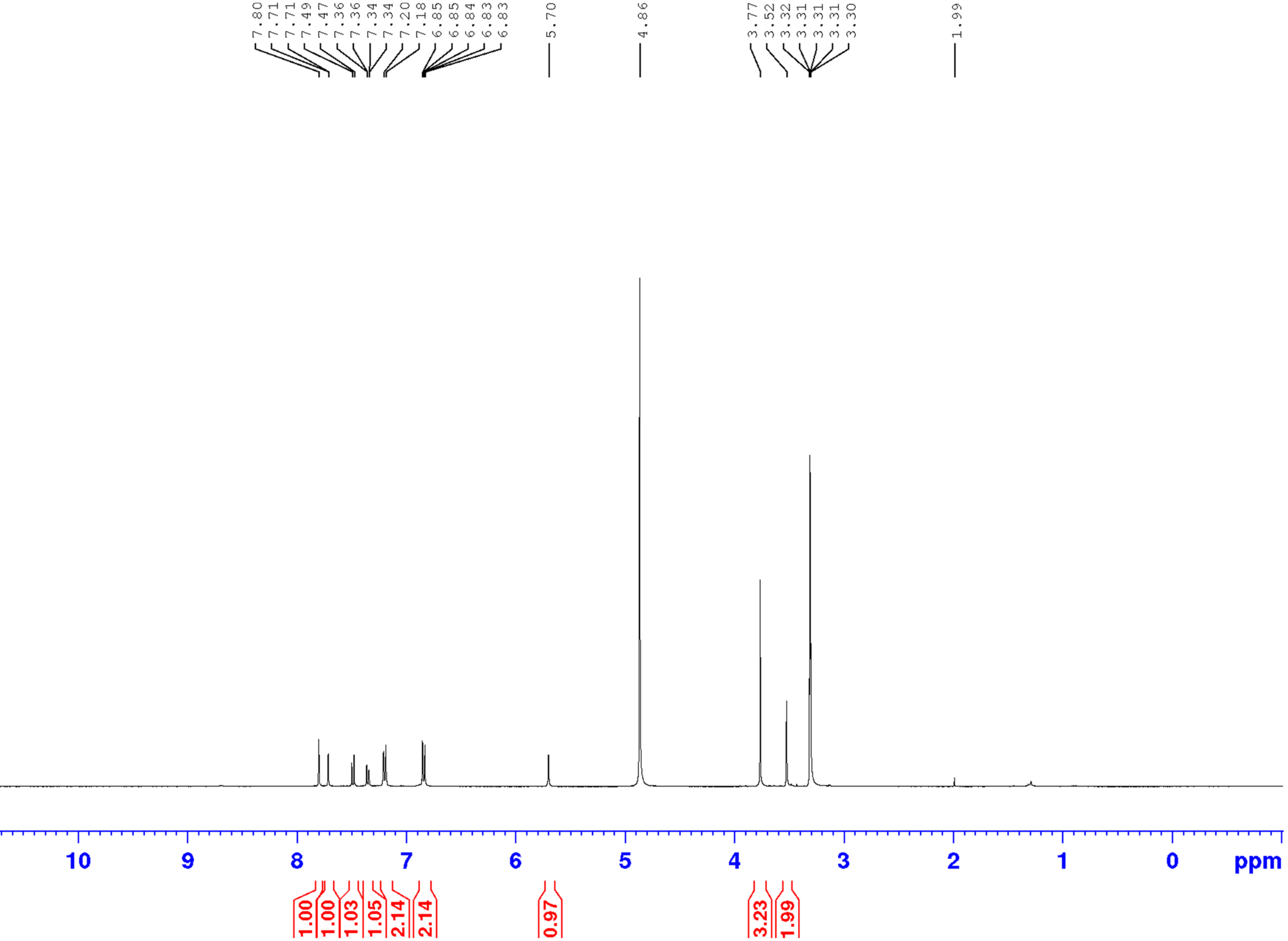

^{1}H-NMR (500 MHz, DMSO-d_6) of **2-(6-Bromobenzofuran-3-yl)-2-(2-(2-fluorophenyl)acetamido)acetic acid, 49**

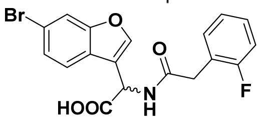

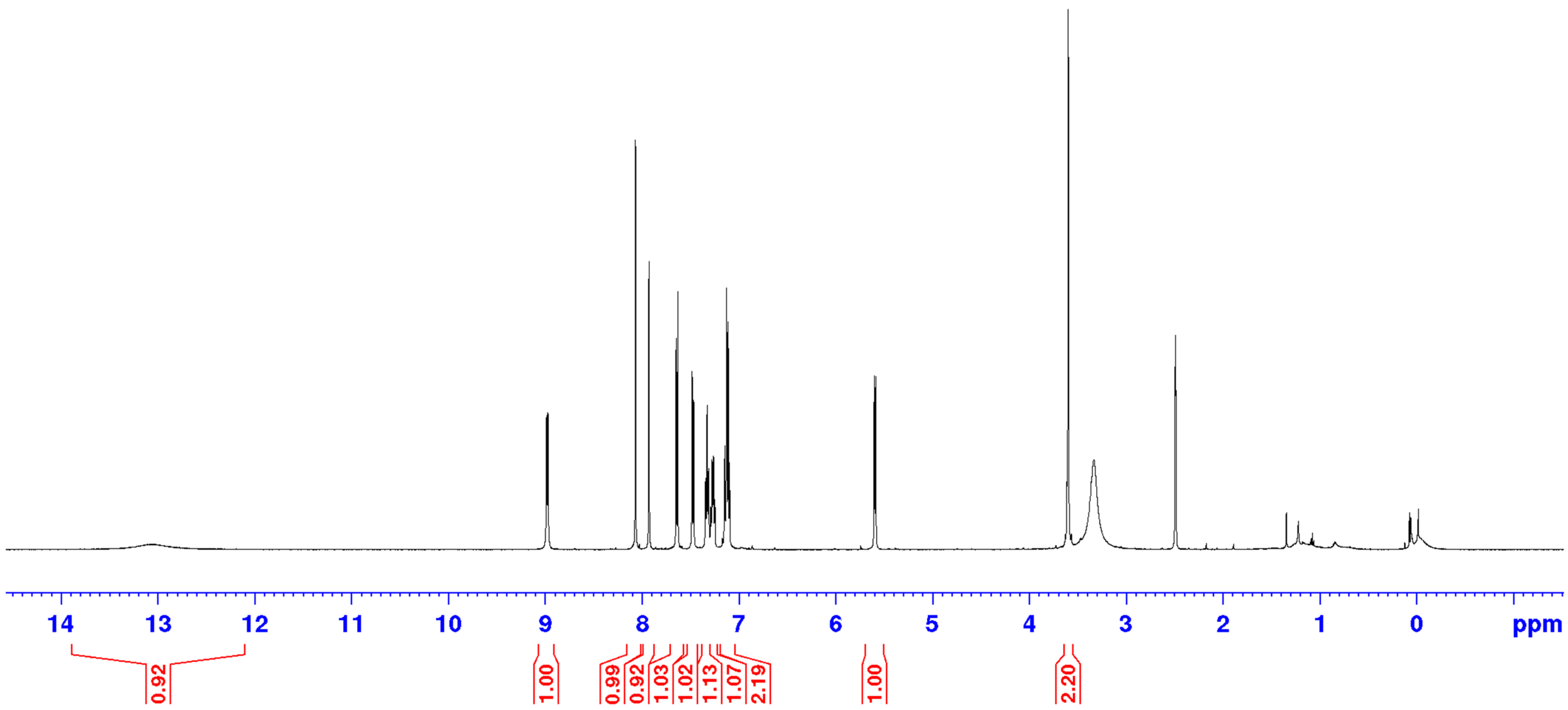

^{13}C-NMR (125 MHz, DMSO-d_6) of **2-(6-Bromobenzofuran-3-yl)-2-(2-(2-fluorophenyl)acetamido)acetic acid, 49**

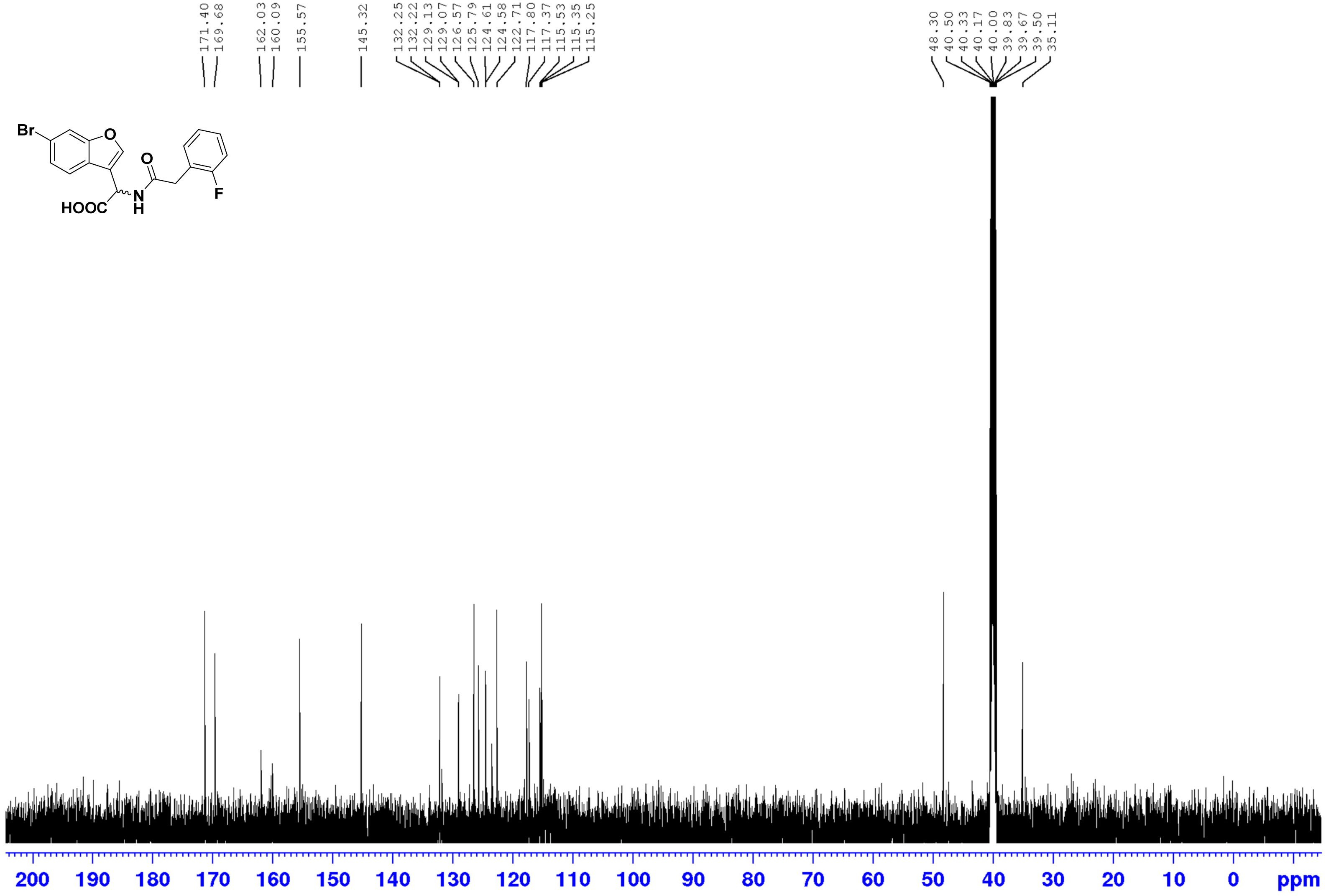

^{1}H-NMR (400 MHz, DMSO-d_6) of **2-(6-Bromobenzofuran-3-yl)-2-(methylsulfonamido)acetic acid, 50**

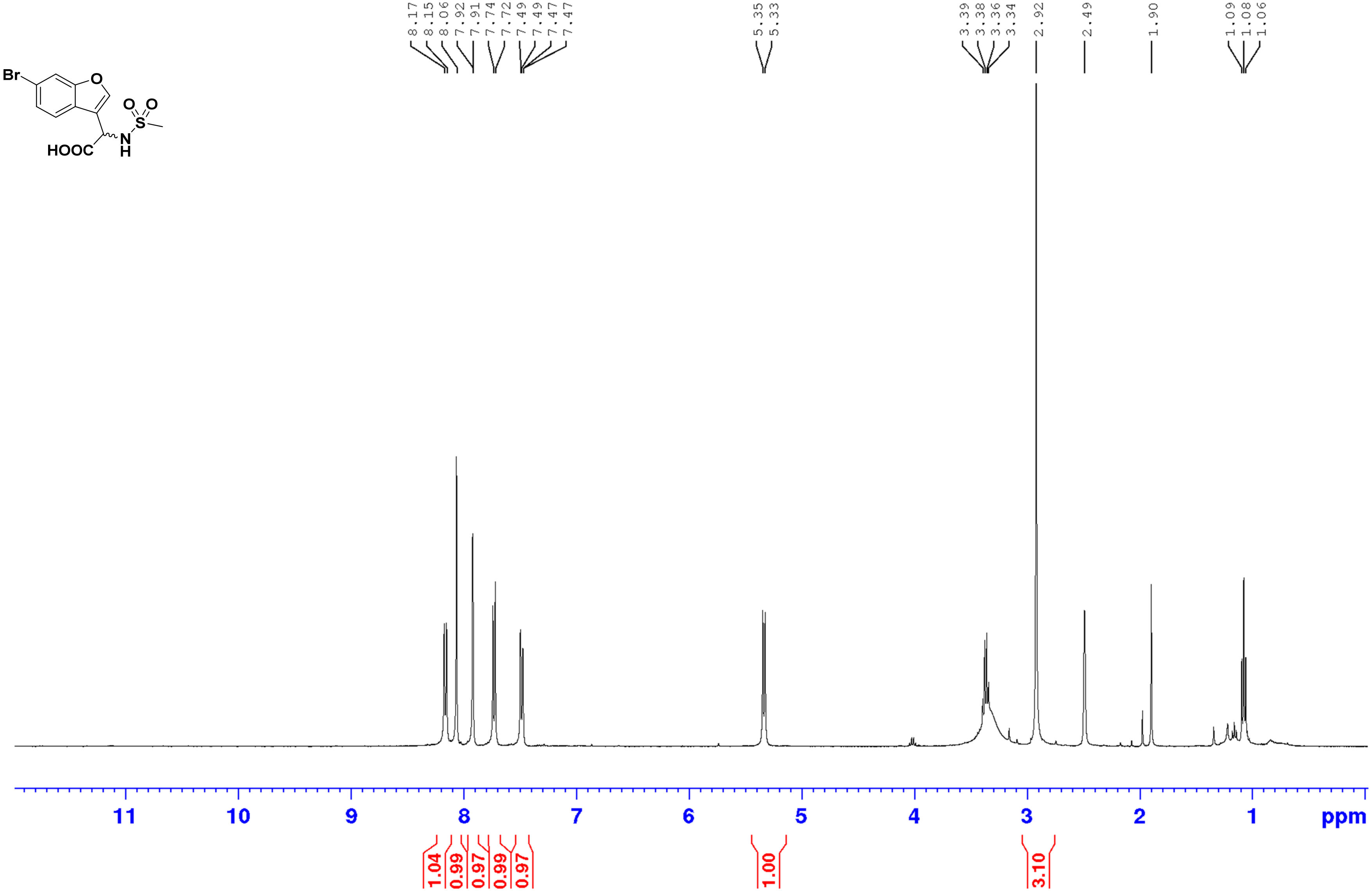

^{13}C-NMR (100 MHz, DMSO-d_6) of **2-(6-Bromobenzofuran-3-yl)-2-(methylsulfonamido)acetic acid, 50**

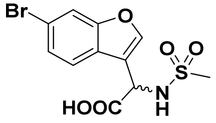

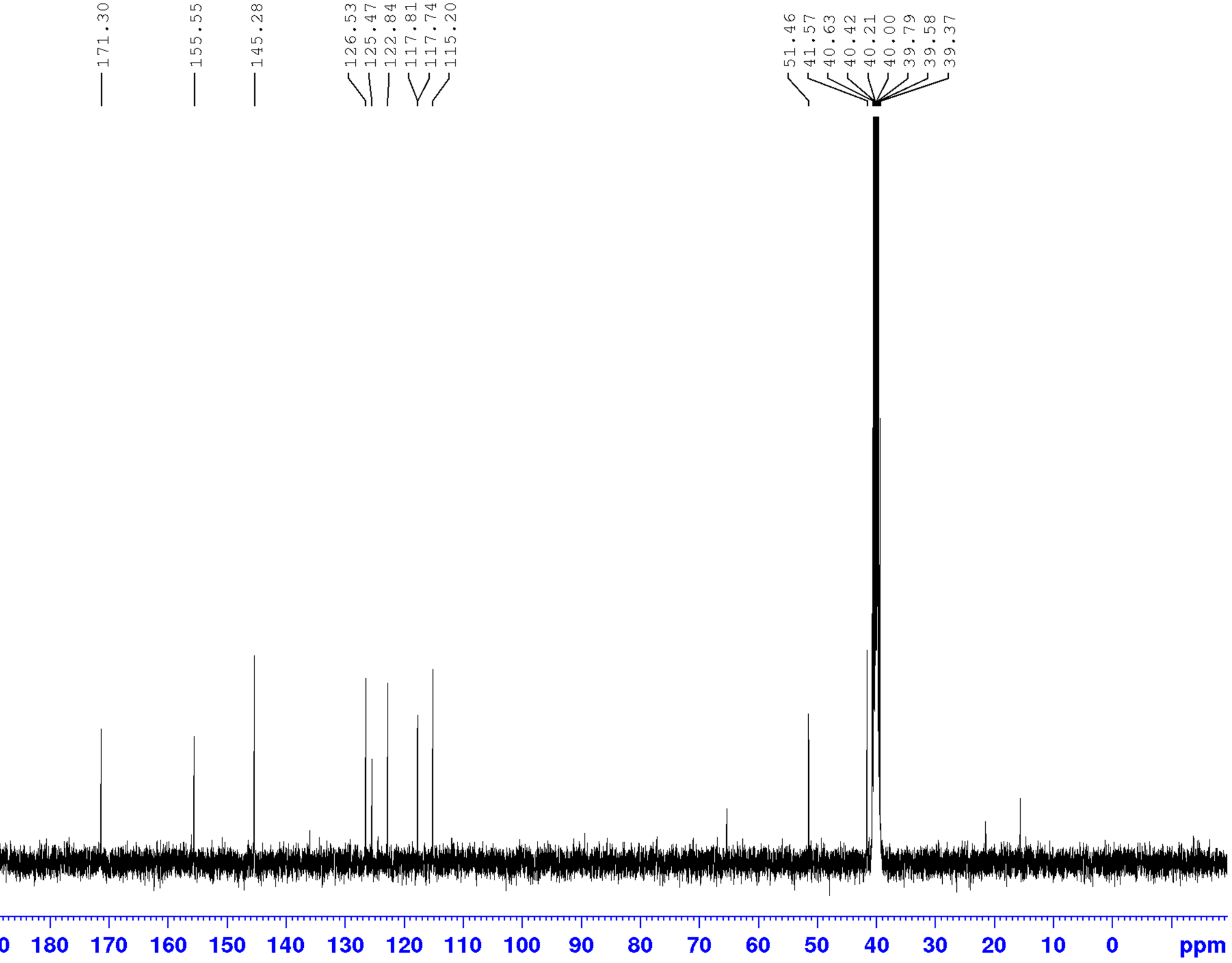

^{1}H-NMR (500 MHz, acetone-d_6) of **2-(6-Bromobenzofuran-3-yl)-2-(phenylsulfonamido)acetic acid, 51**

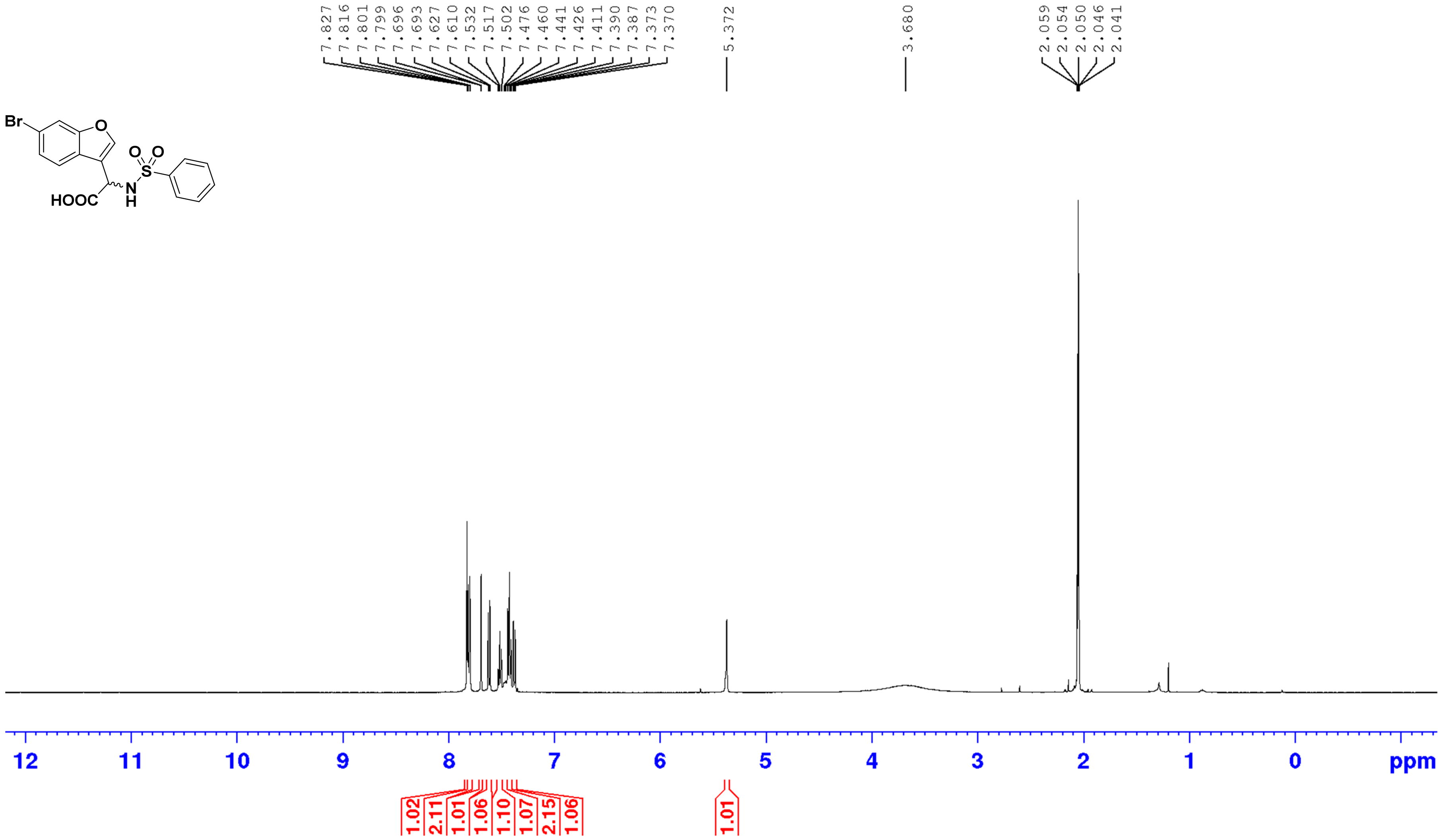

^{13}C-NMR (125 MHz, acetone-d_6) of **2-(6-Bromobenzofuran-3-yl)-2-(phenylsulfonamido)acetic acid, 51**

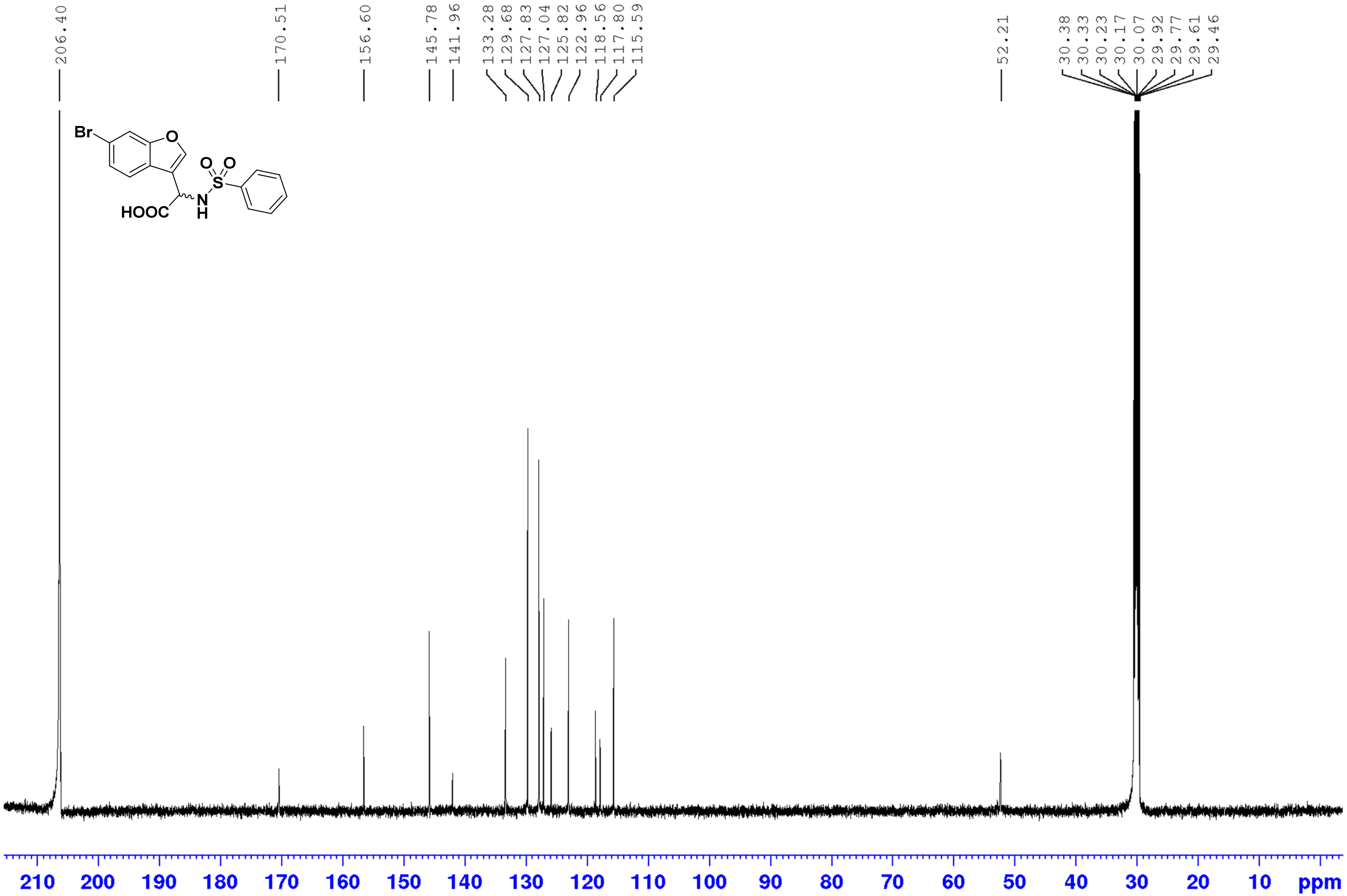

Supplement: Supplementary file 1 [file molecules-24-03756-s001.pdf]
